# Synthesis of a molecularly defined single-active site heterogeneous catalyst for selective oxidation of *N*-heterocycles

**DOI:** 10.1038/s41467-018-03834-4

**Published:** 2018-04-13

**Authors:** Yujing Zhang, Shaofeng Pang, Zhihong Wei, Haijun Jiao, Xingchao Dai, Hongli Wang, Feng Shi

**Affiliations:** 1 State Key Laboratory for Oxo Synthesis and Selective Oxidation, Center for Green Chemistry and Catalysis, Lanzhou Institute of Chemical Physics, Chinese Academy of Sciences, 730000 Lanzhou, China; 2 University of Chinese Academy of Sciences, 100049 Beijing, China; 3 Key Laboratory of Environmental Friendly Composite Materials and Biomass in Universities of Gansu Province, Northwest Minzu University, 730000 Lanzhou, China; 4 Leibniz-Institut für Katalyse e.V. an der Universität Rostock (LIKAT), Albert-Einstein-Str. 29a, 18059 Rostock, Germany

## Abstract

Generally, a homogeneous catalyst exhibits good activity and defined active sites but it is difficult to recycle. Meanwhile, a heterogeneous catalyst can easily be reused but its active site is difficult to reveal. It is interesting to bridge the gap between homogeneous and heterogeneous catalysis via controllable construction of a heterogeneous catalyst containing defined active sites. Here, we report that a molecularly defined, single-active site heterogeneous catalyst has been designed and prepared via the oxidative polymerization of maleimide derivatives. These polymaleimide derivatives can be active catalysts for the selective oxidation of heterocyclic compounds to quinoline and indole via the recycling of –C=O and –C–OH groups, which was confirmed by tracing the reaction with GC-MS using maleimide as the catalyst and by FT-IR analysis with polymaleimide as the catalyst. These results might promote the development of heterogeneous catalysts with molecularly defined single active sites exhibiting a comparable activity to homogeneous catalysts.

## Abstract (web summary)

Synthesis of molecularly defined single-active site catalytic materials is the key to bridging the gap between homogeneous and heterogeneous catalysis. Here, the authors develop such catalysts for selective oxidation of heterocyclic compounds to quinoline and indole via the oxidative polymerization of pyrrole derivatives.

## Introduction

As single-active site catalysts, homogeneous catalysts have the advantage of being well defined on a molecular level and readily soluble in the reaction medium. They are highly accessible to the substrates and often show high catalytic activity and selectivity under mild conditions. However, homogeneous catalysts, i.e., transition metal complexes or organic molecules, often consist of valuable noble metals and/or ligands and face the problem of separation. Thus, despite their intrinsic advantages, homogeneous catalysts are used in less than 20% of the industrially relevant processes because they are not easily recycled. Although heterogeneous catalysts have been extensively used in practical processes due to their easy recoverability and operation, they often possess different catalytically active sites, and testing them on a molecular level is difficult. In fact, various approaches have been implemented in the past decades to combine the advantages of homogeneous catalysis with those of heterogeneous catalysis. Typical examples for such efforts include solid support-immobilized metal complexes, single atom catalysts, organic–inorganic hybrid materials, nanocarbon materials, etc.^1–7^, which are widely applied in catalytic transformation processes. These results help us understand the relationship between homogeneous and heterogeneous catalysis. However, there is still a long pathway toward bridging these catalysis research areas.

**Fig. 1:**
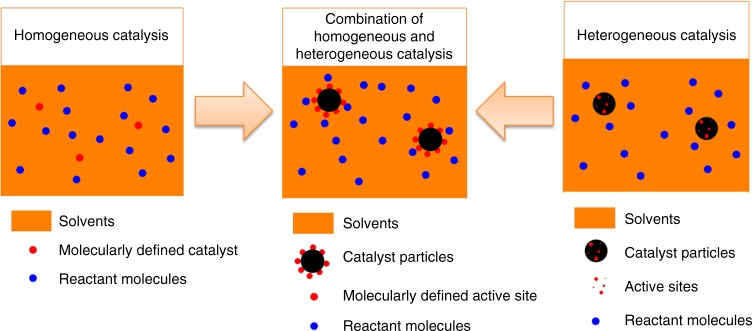
Construction of single-site heterogeneous catalyst. This catalyst combines the advantages of homogeneous catalyst, i.e., molecularly defined active site, and heterogeneous catalyst, i.e., easy to be recovered and reused

Typically, catalysts that combine homogeneous and heterogeneous features should possess the following characteristics^8^. First, the catalyst should be composed of molecularly defined active sites, and second the catalyst should be insoluble in the solution during the reaction in order to build a heterogeneous catalyst system. Therefore, an illustration of this concept is given below (Fig. 1).

Carbon catalysts have been widely applied in the selective oxidation of alkanes^9–13^, alcohols^14–18^, amines^19–22^, thiols and sulfides^5,23^, the nucleophilic addition of alcohol to an epoxide^24^, aldehyde acetalization or esterification^24,25^, Michael addition reactions^26,27^, F-C addition of indoles to α,β-unsaturated ketones^28^ and the synthesis of dipyrromethane^29^, the catalytic reduction of olefin^30,31^ and nitrobenzene^32^, C-heteroatom coupling reactions^7,33,34^ and heterocycles dehydrogenative aromatization^35–37^, etc.

Among the above transformations, active sites on the carbon catalysts toward selective oxidation or catalytic reduction include –C–OH and –C=O. The oxidation or reduction reactions can be realized via the recycling of the –C–OH and –C=O groups. Inspired by these results, in this work, a molecularly defined single-site heterogeneous catalyst has been designed and prepared via the oxidative polymerization of pyrrole derivatives. Catalytic performance exploration showed that these polymaleimide (PMI) derivatives can be active catalysts for the selective oxidation of heterocyclic compounds to quinoline and indole via the recycling of –C=O and –C–OH groups.

## Results

### Exploration of active molecular catalysts

**Fig. 2:**
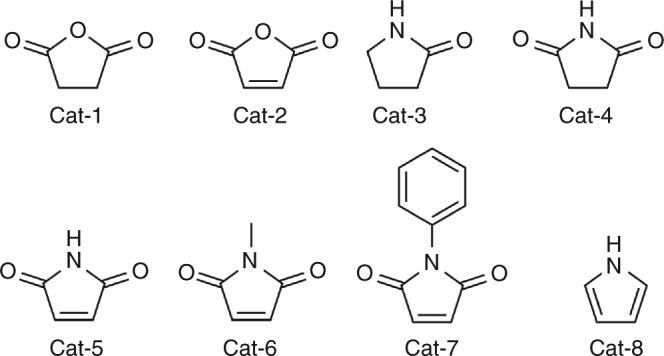
Molecular catalysts involved in the current work. Different molecules containing carbonyl groups were used as potential catalysts for the selective oxidation of *N*-heterocycles. Pyrrole was used as a control catalyst to exclude the contribution of olefin group

These results provide strong inspiration to design molecularly defined heterogeneous catalysts for selective oxidation reactions, and the key to realizing this idea is the development of a method to synthesize molecularly defined heterogeneous catalytic materials. In the initial stage, the following molecules containing carbonyl groups as molecularly defined catalysts were tested (Fig. 2).

The oxidative dehydrogenation of *N*-heterocycles is used to synthesize high-value products, such as quinoline, isoquinoline, quinazoline, and indole derivatives, most of which can behave as bioactive compounds, pharmaceutical molecules and functional materials, and this remains an interesting topic in organic synthesis. Various catalysts, including transitional metal-based^38–59^ and carbon-based catalysts^36,37^, have been studied extensively. Due to the importance of this transformation, it was chosen as the model reaction to test the catalytic performance of these carbonyl group-containing molecules.

As shown in Table 1, no reaction occurred if the selective oxidation of 1,2,3,4-tetrahydroquinoline was performed under catalyst-free conditions (Entry 1). The conversion of 1,2,3,4-tetrahydroquinoline reached 86% with the use of succinic anhydride (Cat-1) as a catalyst; some quinoline *N*-oxide was formed, and the selectivity was only 44% (Entry 2). The conversion of 1,2,3,4-tetrahydroquinoline was >99% if maleic anhydride (Cat-2) was employed (Entry 3). However, only a trace amount of the desired product was observable. Better results were obtained if maleimide derivatives (Cat-3~7) were used as catalysts, and 48–100% conversion of 1,2,3,4-tetrahydroquinoline with 40–71% selectivity was obtained (Entries 4–8). Additionally, the catalytic activity of pyrrole (Cat-8) was tested, and the conversion of 1,2,3,4-tetrahydroquinoline was only 4% (Entry 9). Therefore, the carbonyl group but not the olefin group was responsible for the selective oxidation reaction, which means that these molecules containing carbonyl groups show potential as catalysts for the selective oxidation reaction. To examine the generated byproducts, the reactions were traced by GC-MS, and a significant amount of additional products of 1,2,3,4-tetrahydroquinoline to cat-2, 5, 6, and 7 were observed. For example, ~40% of 1,2,3,4-tetrahydroquinoline was converted into 3-(3,4-dihydroquinolin-1(2 H)-yl) pyrrolidine-2,5-dione if cat-5 was used as the catalyst. In addition, some quinoline *N*-oxide and tar-like materials were also formed as byproducts.

**Table 1:** Catalyst screening and reaction condition optimization with molecular catalysts


Entry	Catalyst	Conversion (%)^a^	Selectivity (%)^a^
1	0	Trace	Trace
2	Cat-1	86	44
3	Cat-2	>99	Trace
4	Cat-3	29	43
5	Cat-4	48	40
6	Cat-5	100	49
7	Cat-6	100	71
8	Cat-7	91	70
9	Cat-8	4	56

Reaction conditions: 0.5 mmol catalyst, 0.5 mmol 1,2,3,4-tetrahydroquinoline, 1 atm O_2_, solvents (1 mL H_2_O + 1 mL CH_3_OH), 120 °C (oven temperature), 300 rps, 24 h

^a^ The conversion and selectivity were determined by GC-FID with the external standard method

### Polycatalysts preparation and catalytic activity test

**Fig. 3:**
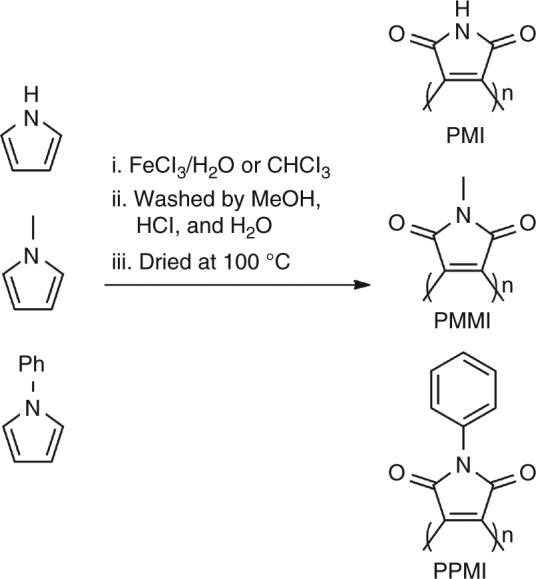
Polycatalysts synthesized in the current work. PMI polymaleimide, PMMI poly-N-Methyl-maleimide, PPMI poly-N-phenyl-maleimide

Based on the above results, we tried to build heterogeneous catalysts with well-defined molecular structures and active sites. Three polymer catalysts with pyrrole, N-methylpyrrole, and N-benzylpyrrole as monomers were prepared via oxidative polymerization with FeC1_3_^60^ (Fig. 3). First, a certain amount of pyrrole derivatives was added to an FeCl_3_ solution, and the mixture was magnetically stirred at room temperature for 4 h to form black powders. Then, the obtained black powders were filtered and washed with methanol, a mixed solution of methanol and 36 wt% HCl, and water to remove the residual iron species. The powders were then dried in an electrothermal constant temperature dry box in air at 100 °C for 12 h. Three polycatalysts were prepared by using this method by varying the monomers. Polymaleic anhydride was not prepared and tested in this work because it decomposes at approximately 100 °C. These polymers were then applied in the selective oxidation of 1,2,3,4-tetrahydroquinoline to test their catalytic performance.

Fortunately, good to excellent results were obtained, and a >99% yield of the desired product was achieved when PMI was used as the catalyst (Table 2, Entries 1–3). Notably, PMI can be easily recovered and reused when it is a catalyst, and a 94% yield was maintained at the 3rd run (Entry 1). This result means that the construction of a heterogeneous catalyst with molecularly defined active sites was realized, and the addition of amine to the catalyst monomer was successfully inhibited. Additionally, PMI can work well under reduced loading (Entries 4–7). For example, a 95% 1,2,3,4-tetrahydroquinoline conversion with 79% quinoline selectivity can be obtained if 20 mg PMA was added as a catalyst. If lower catalyst loadings were employed, the conversion and selectivity were simultaneously decreased. For example, 39% conversion was still obtained if 3 mg of PMA was used, while the selectivity decreased to 24%. The main byproduct was quinoline *N*-oxide. Finally, considering the possible effects of trace amounts of Fe species maintained in the polymer catalysts (0.454 wt%, determined by ICP-AES), the Fe-catalyzed oxidation of 1,2,3,4-tetrahydroquinoline was performed (Entries 9–10) and much lower conversion values of 1,2,3,4-tetrahydroquinoline and selectivity to quinoline were obtained, which show a decreased possibility of Fe ions as active species. However, it is still necessary to further exclude the role of Fe species in the oxidation reactions. Therefore, two control reactions were performed. First, 0.05 mmol acetylacetone was added to shield the Fe species under the same reaction conditions, and a 95% yield of quinoline was obtained. In addition, Fe-free PMI was prepared with ammonium persulfate as the oxidant, and a 91% yield of quinoline was obtained by applying this Fe-free PMI as a catalyst. Based on these results, the contribution from the Fe species to the selective oxidation reaction can be excluded. Notably, after being centrifugally separated, this catalyst can be reused after being washed with MeOH (50 mL × 4) and dried in vacuum at 50 °C for 3 h, and an 89% quinoline yield was still obtained when it was used for a 5th cycle (Entry 1). Therefore, this catalyst exhibits nice reusability.

**Table 2:** Catalyst screening and reaction condition optimization with polycatalysts

Entry	Catalyst/mg	Conversion (%)^a^	Selectivity (%)^a^


Reaction conditions: 0.5 mmol 1,2,3,4-tetrahydroquinoline, 1 atm O_2_, solvents (1 mL H_2_O + 1 mL CH_3_OH), 120 °C (oven temperature), 300 rps, 24 h

^a^ The conversion and selectivity were determined by GC-FID with the external standard method

^b^ The catalyst was used for a 5th cycle

### Selective oxidation of *N*-heterocycles catalyzed by PMI

Having obtained the optimized polycatalyst and reaction conditions, the scope and generality of this catalyst system was subsequently explored. As shown in Table 3, 1,2,3,4-tetrahydroquinoline and its derivatives with methyl substituents at the 6 position were converted to the corresponding quinolines with a 92–95% yield (Entries 1 and 2). Notably, a more sterically hindered functional substrate, such as 2-methyl-1,2,3,4-tetrahydroquinoline, was also well tolerated, and 2-methylquinoline was obtained with a 91% yield (Entry 3). No apparent electronic effects were observed and a 76–96% yield was obtained when the 1,2,3,4-tetrahydroquinoline derivatives bearing electron-donating groups such as –OMe and electron-withdrawing groups such as –F and –Br were used as the starting materials (Entries 4–6). Gratifyingly, this catalyst system is also applicable for substrates with an oxidizable –OH group, and quinolin-6-ol, quinolin-7-ol and quinolin-8-ol were produced with 93, 89, and 85% yields, respectively (Entries 7–9). A disubstituted substrate, such as 6-fluoro-2-methyl-1,2,3,4-tetrahydroquinoline, was also tested, and up to a 90% yield of 6-fluoro-2-methylquinoline was obtained (Entry 10). Apart from the 1,2,3,4-tetrahydroquinoline derivatives, this catalyst system is also active for the selective oxidation of other heterocyclic substrates, such as 1,2,3,4-tetrahydroisoquinolines, indolines, and 1,2,3,4-tetrahydroquinoxaline. Isoquinoline was formed with a 79% yield, although a higher reaction temperature and longer reaction time were necessary (Entry 11). A suitable result was also obtained if a starting material containing an electron-donating –OH group at the 6 position was used, providing an 87% isolated yield of the desired product (Entry 12). In contrast, an electron-withdrawing group such as –Br had no significant influence on the activity of this catalytic system (Entry 13). The steric-hindrance effect can be observed in the case of 1-methyl-1,2,3,4-tetrahydroisoquinoline and 1-phenyl-1,2,3,4-tetrahydroisoquinoline, and lower yields were provided (Entries 14–15). It should be noted that, apart from the formation of dihydroisoquinolines, isoquinolines were observed as the main byproducts when tetrahydroisoquinoline derivatives were applied as the starting materials. Compared with 1,2,3,4-tetrahydroquinolines and 1,2,3,4-tetrahydroisoquinolines, indolines were noticeably better as starting materials in our catalyst system considering the lower reaction temperature, high yield of the desired products, and good functional group tolerance (Entries 16–21). However, the oxidation of the substrate with a strong electron-withdrawing group, such as –NO_2_, required a higher reaction temperature and longer reaction time (Entry 22). Under the optimized reaction conditions, quinoxaline was also synthesized with a 73% yield (Entry 23).

**Table 3:** Selective oxidation of *N*-heterocycles catalyzed by PMI

Reaction conditions: 0.5 mmol heterocyclic compounds, 50 mg of the PMI catalyst, 1 atm O_2_, solvents (1 mL H_2_O + 1 mL CH_3_OH), 120 °C (oven temperature), 300 rps, 24 h

^a^ Isolated yields

^b^ 140 °C (oven temperature), 36 h

^c^ 160 °C (oven temperature), 36 h

^d^ 80 °C (oven temperature), 24 h

^e^ 100 °C (oven temperature), 24 h

In addition, this reaction is easily performed with a gram-scale starting material. For example, a 91% isolated quinoline yield was obtained when 10 mmol (1.33 g) tetrahydroquinoline was used as the starting material under the same reaction conditions.

### Catalyst characterization and reaction mechanism exploration

Next, the structure of the polycatalysts was explored with characterization by different methods. The EA tests showed that the mass content of C, O, N, and H in PMI, PMMI, and PPMI was 52.26:29.34:14.64:3.76, 57.5:24.7:13.08:2.3, and 68.17:16.85:7.8:7.18, respectively. These test results were close to the theoretical numbers of the polymers, which support the formation of the desired structures. To further verify the structure, ^13^C NMR characterizations were performed (Supplementary Fig. 1), and the ^13^C spectra of –C=C– and –CO_2_NH– were observed^61^. These results also support the formation of the desired structure. In addition, the BET surface of the PMI, PMMI, and PPMI catalysts was 22.5, 87.0, and 87.3 m^2^, respectively. Therefore, the enhanced catalytic performance of PMI should not be attributed to the surface area. Unfortunately, it is difficult to obtain the molecular weights of the polymers as they are not soluble in available solvents such as H_2_O, DMF, DMSO, and others^62,63^.

**Fig. 4:**
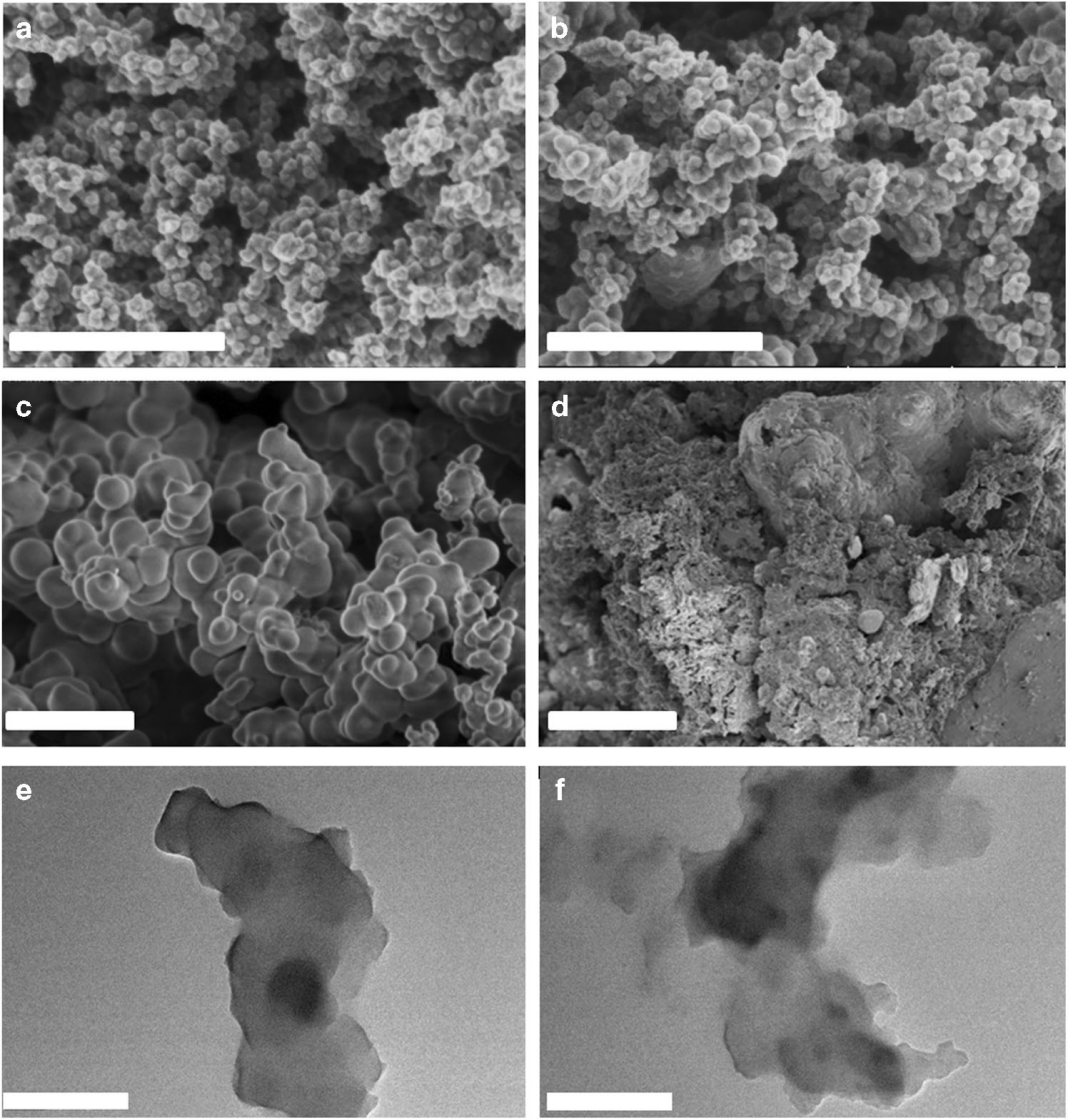
SEM images of catalysts. **a** PMI, **b** PMI used five times, **c** PMMI, and **d** PPMI. **e, f** TEM images of PMI (**e**) and PMI used five times (**f**). The scale bars are1 μm in **a**, **b**, **c**, **d**, and 600 nm in **e** and **f**

The morphology of the polycatalysts was then characterized by scanning electron microscopy (SEM) and TEM (Fig. 3). The results showed that the PMI catalyst is composed of small uniform nanoparticles, while larger particle units and unordered bulk structures were formed in the case of PMMI and PPMI, respectively. To explore the stability of the active PMI catalyst, the sample, after being used five times, was also tested by SEM and TEM. Clearly, only slight aggregation was observed, implying that the polycatalyst is relatively stable during the oxidation reaction (Fig. 4).

The same results can be confirmed by the similar surface components of the PMI catalyst before and after use, which is determined by X-ray photoelectron spectroscopy (XPS) characterization (Supplementary Figs. 2–4). The C, N, and O content on the surface was 80.14, 10.41, and 9.45% (fresh sample) and 82.56, 8, and 9.44% (after use), respectively.

To understand the reaction mechanism well, the variation in the MI catalyst structure during the selective oxidation of 1,2,3,4-tetrahydroquinoline was explored by GC-MS. Clearly, 1,2,3,4-tetrahydroquinoline was completely consumed in 3 h, and the addition product of 1,2,3,4-tetrahydroquinoline and the MI catalyst was observed (Supplementary Fig. 5). In addition, in the early stage of the oxidation reaction, only the addition product (*m*/*z* = 230) was formed because the MI catalyst and the 1,2,3,4-tetrahydroquinoline substrate were abundant in the medium. A new peak corresponding to the addition product (*m*/*z* = 232) appeared when the reaction progressed. The product may form during the recycling of –C=O to –C–OH, and it behaves as the active site of the oxidation reaction. Interestingly, the addition product of 1,2,3,4-tetrahydroquinoline and MI decreased as the reaction progressed, and quinoline was simultaneously generated. Finally, 49% quinoline was obtained, and approximately 50% of the addition product was still maintained. Therefore, the addition product should behave as the intermediate during the reaction, and the recycling between carbonyl and hydroxyl groups might be important in the selective oxidation of tetrahydroquinoline.

Inspired by the above results, the PMI-catalyzed oxidation of 1,2,3,4-tetrahydroquinoline likely proceeds via a similar mechanism as that for the MI catalyst. To verify this proposal, Fourier transform infrared (FT-IR) characterization was performed. As shown in Supplementary Figs. 6–7, PMI has a similar skeletal structure to MI, and the formation of carbonyl groups can be clearly observed at 1647–1782 cm^−1^. Moreover, the FT-IR spectra of the PMI catalyst after use were almost the same as those for the fresh sample, which proved that the PMI catalyst has good stability during the oxidation reaction. Notably, a new peak at 3698 cm^−1^ appeared after being used five times; this peak might be assigned to the –OH stretching bands in PMI, which are generated via the reaction of the –C=O bond with the starting material. From these results, the catalytic recycling of the –C=O and –OH groups can be proposed.

To obtain insight into the reaction mechanism, we attempted to determine whether any hydrogen peroxide was produced during the reaction as it is not common that molecular oxygen can be activated easily under these reaction conditions. A control reaction was performed under the same reaction conditions given in Table 2, Entry 1 without the addition of 1,2,3,4-tetrahydroquinoline. The hydrogen peroxide generated in situ during the oxidation reaction was determined with N,N-diethyl-*p*-phenylenediamine sulfate (DPD) as an indicator because it is easily oxidized to form DPD^+^, which has a strong absorption maximum at 551 nm^64^. Interestingly, the formation of hydrogen peroxide during the reaction was clearly observed, and the hydrogen peroxide concentration in the reaction system is ~1.78 × 10^−4^ mol/L (Supplementary Fig. 8), which might be one of the potential oxidants in the selective oxidation reaction^59^.

**Fig. 5:**
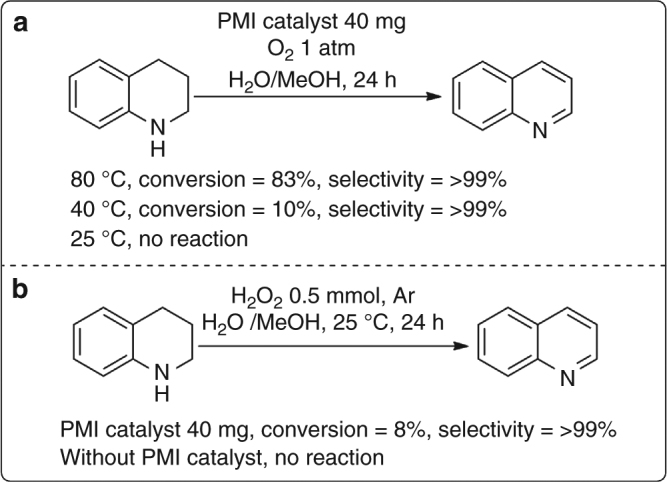
The control experiments with O_2_ or H_2_O_2_ as oxidant. The results of the selective oxidation of 1,2,3,4-tetrahydroquinoline with O_2_ (**a**) or H_2_O_2_ (**b**) as the oxidant at different reaction temperatures

To explore the real role of hydrogen peroxide, some control experiments were performed (Fig. 5). The results of the selective oxidation of 1,2,3,4-tetrahydroquinoline catalyzed by the PMI catalyst at different reaction temperatures (Fig. 5a) showed that our catalyst system has a high selectivity for the formation of quinoline products, although the conversion of 1,2,3,4-tetrahydroquinoline decreased with decreasing reaction temperature. At room temperature, this PMI catalyst was totally inert for the conversion of 1,2,3,4-tetrahydroquinoline, which means that molecular oxygen cannot oxidize 1,2,3,4-tetrahydroquinoline with our catalyst system at room temperature. Interestingly, 8% of the 1,2,3,4-tetrahydroquinoline substrate could be converted into quinoline when oxygen was replaced with hydrogen peroxide (Fig. 5b). In addition, the reaction of 1,2,3,4-tetrahydroquinoline and hydrogen peroxide did not occur in the absence of the PMI catalyst, which suggests that the PMI catalyst is necessary in the transformation of 1,2,3,4-tetrahydroquinoline to the quinoline product. Therefore, hydrogen peroxide has a greater possibility of being the oxidant in this reaction.

Based on the discussions and results obtained above, we carried preliminary B3PW91 computations into the catalytic mechanism (Supplementary Tables 1 and 2). Since gaseous O_2_ has a triplet ground state and H_2_O_2_ and H_2_O have singlet ground states, we computed only the thermodynamic data (ground states) and did not include the kinetic data (transition states) due to the problems of spin crossover between the triplet and singlet potential energy surfaces.

At first, we computed the thermodynamics of the reaction between tetrahydroquinoline and 1*H*-pyrrole-2,5-dione (Supplementary Fig. 9). The computed endergonic thermodynamic data (17.22 kcal/mol) shows that, in general, tetrahydroquinoline cannot be oxidized by 1*H*-pyrrole-2,5-dione to quinolone. The first reaction step is endergonic by 17.42 kcal/mol, and the second reaction step is very slightly exergonic by 0.20 kcal/mol despite the aromatic stabilization from 3,4-dihydroquinoline to quinolone. This transformation is thermodynamically inaccessible, and this agrees with our control experiment.

**Fig. 6:**
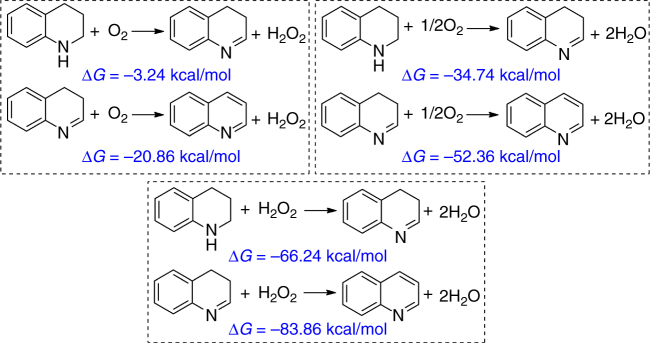
Thermodynamics of tetrahydroquinoline oxidation. The detailed thermodynamics of 1,2,3,4-tetrahydroquinoline oxidation with O_2_ or H_2_O_2_ as the oxidant. The unit is kcal/mol

Next, we computed tetrahydroquinoline oxidation by using molecular and gaseous O_2_. The first reaction is tetrahydroquinoline oxidation by O_2_ resulting in 3,4-dihydroquinoline and H_2_O_2_ and H_2_O (Fig. 6). It shows that tetrahydroquinoline oxidation resulting in H_2_O_2_ as an intermediate is slightly exergonic in the first step (−3.24 kcal/mol) and very exergonic in the second step (−20.86 kcal/mol); the total reaction is thermodynamically accessible. Furthermore, the reaction forming H_2_O as an intermediate is much more exergonic (−34.74 and −52.36 kcal/mol, respectively), also indicating stronger thermodynamic accessibility. In addition, we computed H_2_O_2_ as an oxidation agent, and it is found that the reaction is even more exergonic (−66.24 and −83.86 kcal/mol, respectively) than with O_2_ as an oxidation agent. H_2_O_2_ is a stronger oxidation agent than O_2_, which agrees with our control experiment. Based on these data, we can propose that the first step should be oxidation using molecular O_2_, and the second step can be oxidation using either molecular O_2_ or H_2_O_2_ from the first oxidation step.

In our experiment, we observed the formation of the addition product between tetrahydroquinoline and 1H-pyrrole-2,5-dione resulting in the formation of 3-(3,4-dihydroquinolin-1(2H)-yl)pyrrolidine-2,5-dione, where the S-configuration was used. This step is calculated to be slightly endergonic by 0.65 kcal/mol, indicating a thermodynamic equilibrium and possible reversibility, which agrees with the experimental results.

**Fig. 7:**
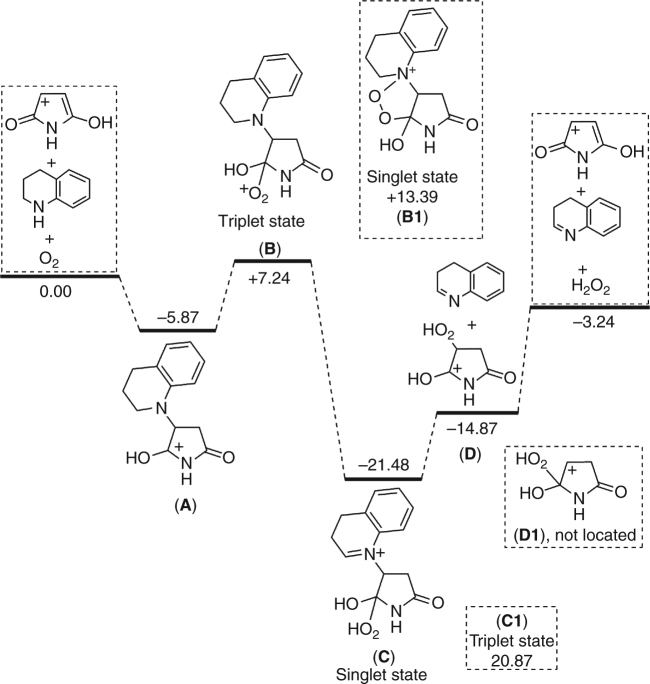
Thermodynamics of the dehydrogenation step. The detailed thermodynamics of 1,2,3,4-tetrahydroquinoline oxidation with O_2_ as the oxidant with maleimide as the model catalyst. The unit is kcal/mol

Finally, we computed the reaction intermediates based on the coupling product (Fig. 7). Since maleimide is acidic with a pKa of 9.46^65^, we computed the protonation of the C=O functional group close to the nitrogen atom of tetrahydroquinoline with the formation of intermediate **A**; this step is slightly exergonic by 5.87 kcal/mol. Starting from intermediate **A**, we computed the O_2_ attack to the positively charged carbon center with the formation of intermediate **B**, which has a triplet ground state, and this step is endergonic by 13.11 kcal/mol. In intermediate **B**, the O_2_ moiety interacts with the carbon center via one O atom. In addition, we also found a corresponding singlet ground state **B1**, in which the second oxygen from O_2_ interacts with the nitrogen atom of tetrahydroquinoline and forms a five-membered ring. It is found that the triplet **B** is more stable than the singlet **B1** by 6.15 kcal/mol. It is interesting to note that the exchange and switch between the triplet **B** and single **B1**, i.e., the optimization of the triplet **B** structure in the singlet state, leads to the five-membered singlet **B1** structure, while the optimization of the five-membered singlet **B1** structure in the triplet state leads to the triplet **B** structure. In addition, the stability of the single **B1** might also explain the formation of quinolone N-oxide (quinoline 1-oxide).

Starting from the triplet **B**, hydrogen abstraction from one C–H bond to form the intermediate **C** is exergonic by 28.72 kcal/mol, and this step is thermodynamically very accessible. In addition, we computed the corresponding triplet **C1**, which is much less stable than the singlet **C** by 42.35 kcal/mol, indicating that the singlet C should be the sole intermediate. Since the triplet **B** is more stable than the singlet **B1** and the singlet **C** is more stable than the triplet **C1**, spin crossover from the triplet **B** to the singlet **C** must occur. Indeed, we tried to locate the cross-point as well as the transition state for C–H abstraction but failed.

Starting from the singlet **C**, we computed the formation of the first dehydrogenation product 1,2-dihydroquinoline, and this step is endergonic by 6.61 kcal/mol. Notably, the proposed **D1** structure cannot be located, and optimization causes **D1** to directly form structure **D** via a shift in the OOH group. Starting from **D**, the elimination of H_2_O_2_ regenerates the catalyst and closes the catalytic cycle, and this step is endergonic by 11.63 kcal/mol. In total, the first dehydrogenation step is exergonic by 3.24 kcal/mol. Finally, 1,2-dihydroquinoline can be easily oxidized to quinoline, as shown by the thermodynamic data in Fig. 6, with or without a catalyst, and this is because unsaturated heterocycles can be easily aromatized under catalyst-free conditions by using only molecular O_2_ under mild conditions^35^.

In summary, PMI as a molecularly defined single-active site heterogeneous catalyst for the oxidative dehydrogenation of *N*-heterocycles was developed successfully. A series of 1,2,3,4-tetrahydroquinoline and indoline derivatives was converted into value-added quinoline, isoquinoline, quinoxaline, and indole derivatives with excellent selectivity, which was ascribed to the trace amount of hydrogen peroxide continuously generated in situ during the selective oxidation. Exploration of the mechanism showed that the –C=O group in the polymer catalyst can be reduced to –OH during the reaction, which supports the catalytic cycles between –C=O and –OH groups. The present results might promote the development of active catalysts to combine the advantages of homogeneous and heterogeneous catalysts.

## Methods

### General

The XPS measurements were performed using a Thermo Scientific ESCALAB 250 instrument with a dual Mg/Al anode X-ray source, a hemispherical capacitor analyzer and a 5 keV Ar^+^ ion gun. All the spectra were recorded using non-monochromatic Mg Kα (1253.6 eV) radiation. FT-IR spectroscopic characterizations were performed on a Nicolet 5700 spectrometer. The sample was prepared by mixing 0.5 mg of carbon material with 100 mg of KBr. SEM was performed with a JEOL JSM-6701F equipped with a cold field emission gun. TEM images were obtained on a Tecnai G2 F30 S-Twin operating at 300 kV. For the prepared catalysts, the particle dispersion was diluted by ethanol, and then 10 µL of the dispersion was cast on the TEM grids with a micro-pipet. Nitrogen adsorption-desorption isotherms were measured at 77 K using an American Quantachrome iQ_2_ automated gas sorption analyzer. The pore-size distribution was calculated by the Barrett–Joyner–Halenda method from the desorption isotherm. Mass spectra were in general recorded on an HP 6890/5973 GC-MS. The Fe content in the catalyst was measured by inductively coupled plasma-atomic emission spectrometry (ICP-AES) using an Iris advantage Thermo Jarrel Ash device. Elemental analysis (C, N, O, and H) of the samples was carried out on a Vario EL microanalyzer. The concentration of H_2_O_2_ was examined with an Agilent 8453 UV-vis spectrophotometer. NMR spectra were measured using a Varian NMR system at 400.1 MHz (^1^H) and 100.6 MHz (^13^C). All spectra were recorded in CDCl_3_ or CD_3_OD, and chemical shifts (δ) were reported in ppm relative to tetramethylsilane referenced to the residual solvent peaks. ^13^C solid state NMR spectra were measured using a Bruker Advance II WB 400 MHz NMR Spectrometer with a 4 mm double-resonance (HX) probe. The optimum CP time (1 ms) was determined as the time for which the maximum ^13^C signal was obtained for selected samples. All calculations were performed by using the Gaussian 16 program. Structural optimizations were carried out at the B3PW91^66^ level of density functional theory with the all-electron TZVP^67^ basis set in the gas phase. All optimized structures were characterized either as energy minimums without imaginary frequencies or transition states with only one imaginary mode by frequency calculations, and the imaginary model is used to connect the initial and the final states. The thermal corrections to the Gibbs free energy at 298 K from the frequency analysis were added to the total electronic energy, and the resulting Gibbs free energies were used for discussion and comparison. For NMR spectra of the compounds in this article, see Supplementary Figs. 10–51.

### Typical procedure for polycatalyst preparation

PMI was synthesized in a closed 500 mL three-necked round-bottomed flask to prevent pyrrole evaporation. First, 3 g of pyrrole was added to an aqueous solution of FeCl_3_ (0.5 M, 200 mL), and the mixture was magnetically stirred at room temperature for 4 h to form a black powder. Then, the obtained black powder was filtered, washed with methanol (200 mL), a mixed solution of methanol and 36 wt% HCl (V/V = 1:1) (200 mL × 5), and water (2.5 L) to remove the residual iron species, and it was dried in an electrothermal constant-temperature dry box in air at 100 °C for 12 h. Finally, the obtained black powder was denoted as PMI, and its yield was 93%.

PMMI was synthesized in a closed 500 mL three-necked round-bottomed flask to prevent N-methylpyrrole evaporation. First, 3.68 g of N-methylpyrrole was added to an aqueous solution of FeCl_3_ (0.5 M, 200 mL), and the mixture was magnetically stirred at room temperature for 4 h to form a black powder. Then, the obtained black powder was filtered, washed with methanol (200 mL), a mixed solution of methanol and 36 wt% HCl (V/V = 1:1) (200 mL × 5), and water (2.5 L) to remove the residual iron species. It was then dried in an electrothermal constant-temperature dry box in air at 100 °C for 12 h. Finally, the obtained black powder was denoted as PMI, and its yield was 96%.

PPMI was synthesized in a closed 500 mL three-necked round-bottomed flask to prevent N-phenylpyrrole evaporation. First, 5.6 g of N-phenylpyrrole was added to a CHCl_3_ solution of FeCl_3_ (0.5 M, 200 mL), and the mixture was magnetically stirred at room temperature for 4 h to form a black powder. Then, the obtained black powder was filtered, washed with methanol (200 mL), a mixed solution of methanol and 36 wt% HCl (V/V = 1:1) (200 mL × 5), and water (2.5 L) to remove the residual iron species, and it was dried in an electrothermal constant-temperature dry box in air at 100 °C for 12 h. Finally, the obtained black powder was denoted as PMI, and its yield was 71%.

### Typical procedure for selective oxidation of *N*-heterocycles

First, 0.5 mmol *N*-heterocycles, 50 mg of PMI catalyst, and 2 mL of solvents (1 mL H_2_O + 1 mL CH_3_OH) were added to a 38 mL glass pressure tube. The pressure tube was exchanged three times with O_2_ and held at 120 °C for 24 h under 300 rps magnetic stirring. After completion of the reaction, the pressure tube was cooled to room temperature, and the crude reaction mixture was purified by column chromatography. The PMI catalyst was separated from the reaction mixture by centrifugation, washed with MeOH (50 mL × 4) and dried in vacuum at 50  °C for 3 h. It was then reused for the selective oxidation of the N-heterocycles.

### Method for H_2_O_2_ determination

H_2_O_2_ was determined with the aid of Fe_3_O_4_ magnetic nanoparticles as a peroxidase mimetic and DPD as a substrate, and the oxidation of DPD was performed at 30 °C. The oxidation product (DPD^+^) of DPD was examined with an Agilent 8453 UV-vis spectrophotometer^64^. The standard curve was recorded in a linear range of 5.0 × 10^−6^ to 5.0 × 10^−4^.

### Procedure for the determination of in situ generated H_2_O_2_

First, 40 mg of the PMI catalyst and 2 mL of solvents (1 mL H_2_O + 1 mL CH_3_OH) were added to a 38 mL glass pressure tube. The pressure tube was exchanged three times with O_2_ and maintained at 120 °C for 10 h under 300 rps magnetic stirring. After the completion of the reaction, the pressure tube was cooled to room temperature, and the reaction mixture was centrifuged. Then, 1.0 mL of the supernatant was added to the mixture solution containing 0.2 mL of NaOAc-HOAc buffer (pH 4.0), 0.5 mL of H_2_O, 0.5 mL of CH_3_OH, 0.4 mL of 1.25 × 10^−2^ mol L^−1^ (DPD), and 0.25 mL of Fe_3_O_4_ MNPs dispersion, and the mixed solution was reacted at 30 °C for 20 min. After the Fe_3_O_4_ MNPs were removed by using a magnet, the supernatant solution was examined with an Agilent 8453 UV-vis spectrophotometer.

### Data availability

All data are available from the authors upon reasonable request.

## Electronic supplementary material

Supplementary Information
