# Supplementary material for: Synthesis of a molecularly defined single-active site heterogeneous catalyst for selective oxidation of N-heterocycles
Source: Nat Commun. 2018 Apr 13;9:1465. doi: 10.1038/s41467-018-03834-4 (PMC5899140; doi:10.1038/s41467-018-03834-4)
Supplement: Supplementary file 1 — Supplementary Information [file 41467_2018_3834_MOESM1_ESM.docx]

**Synthesis of a Molecularly Defined Single Active Site Heterogeneous Catalyst for Selective Oxidation of *N*-Heterocycles**

Zhang et.al

**I. Characterization results of the catalysts**

**
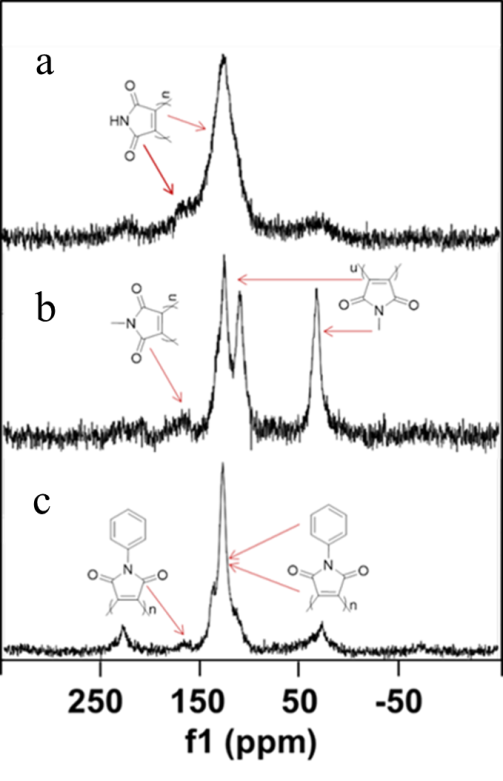
**

**Supplementary Figure 1** ^13^C NMR spectra of PMMI (a), PPMI (b) and PPMI (c).

**
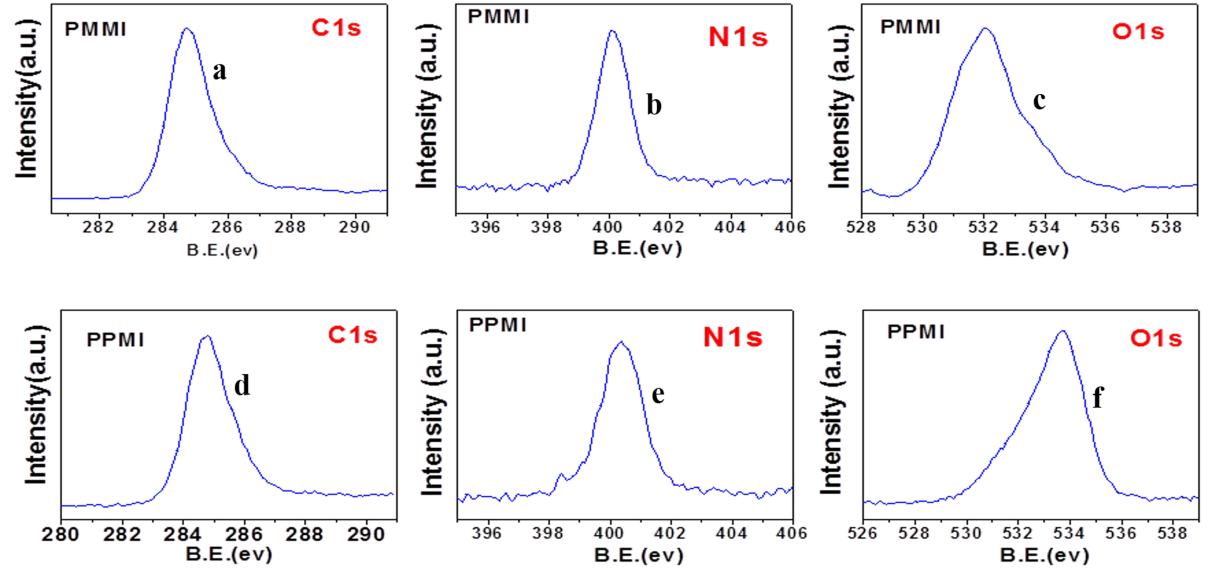
**

**Supplementary Figure 2** XPS spectra of PMMI and PPMI. (a) C1s of PMMI, (b) N1s of PMMI, (c) O1s of PMMI, (d) C1s of PPMI, (e) N1s of PPMI and (f) O1s of PPMI.

**
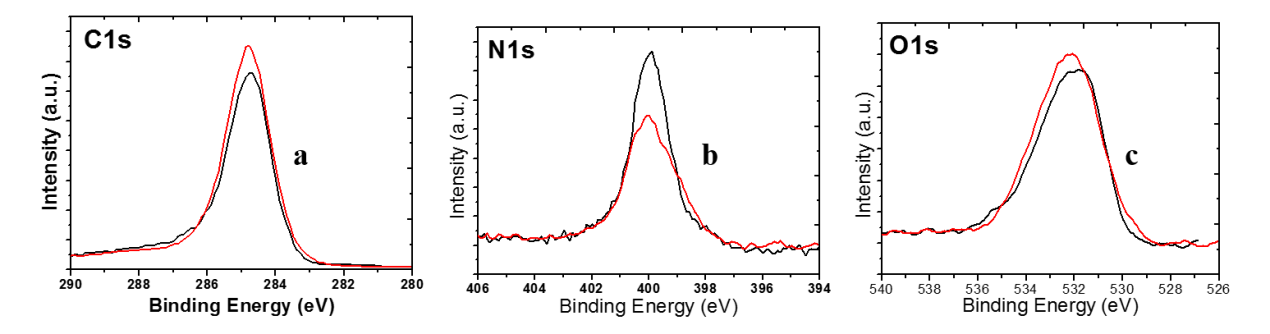
**

**Supplementary Figure 3** XPS spectra of PMI before use (black) and after use (red). (a) C1s, (b) N1s and (c) O1s.


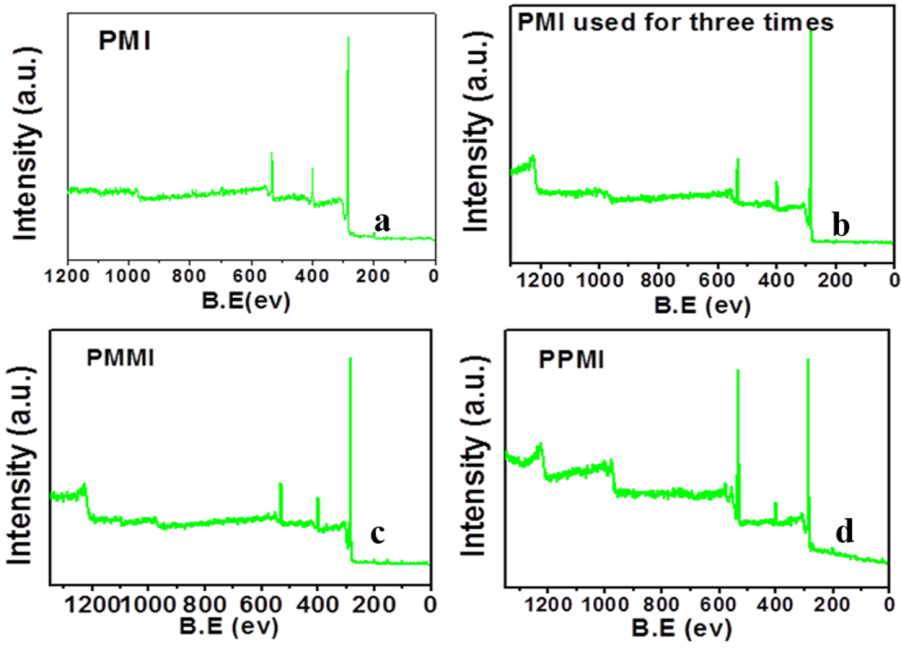


**Supplementary Figure 4** XPS survey scans of PMI (a), PMI used three times (b), PMMI (c) and PPMI (d).

**
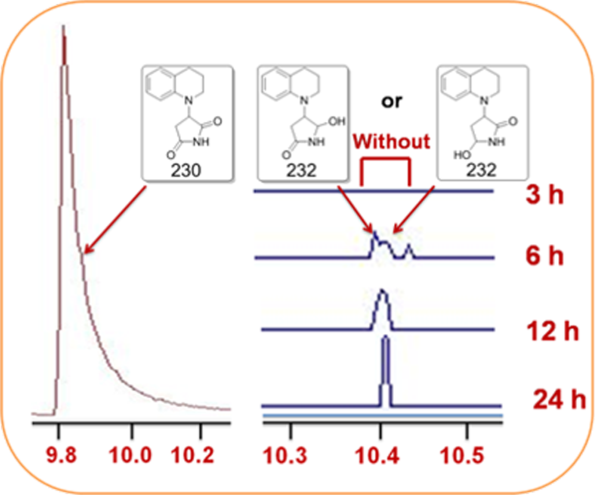
**

**Supplementary Figure 5** Ion selective MS of the addition products of tetrahydroquinoline and maleimide (m/z = 230 and 232)


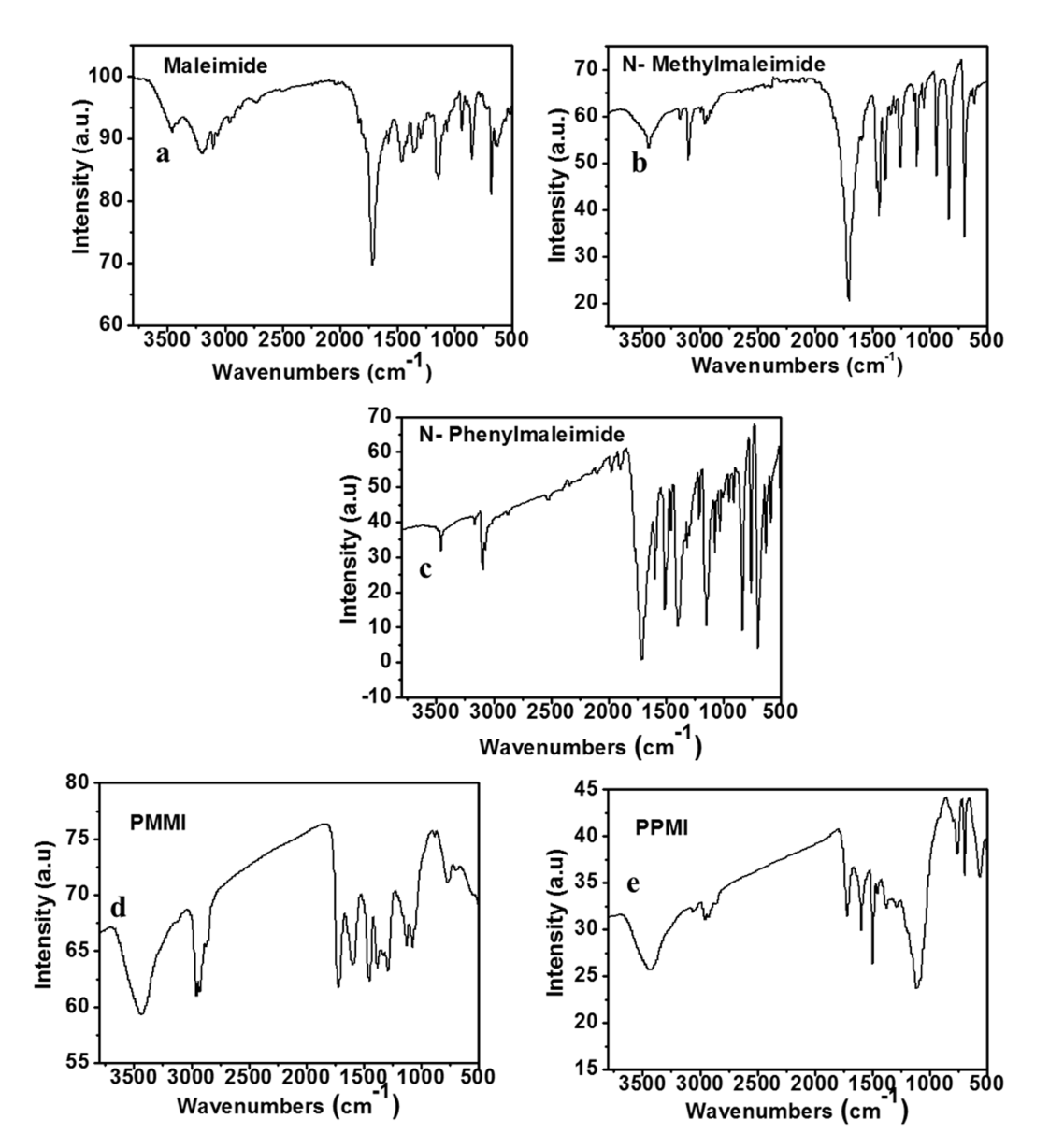


**Supplementary Figure 6** FT-IR spectra of maleimide (a), N-methylmaleimide (b), N-phenylmaleimide (c), PPMI (d) and PPMI (e).


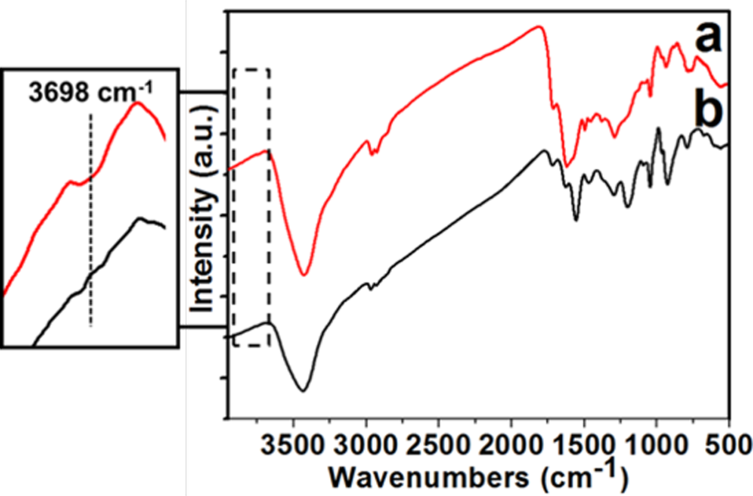


**Supplementary Figure 7** FT-IR spectra of fresh PMI (a) and PMI after being used for 5 times (b).


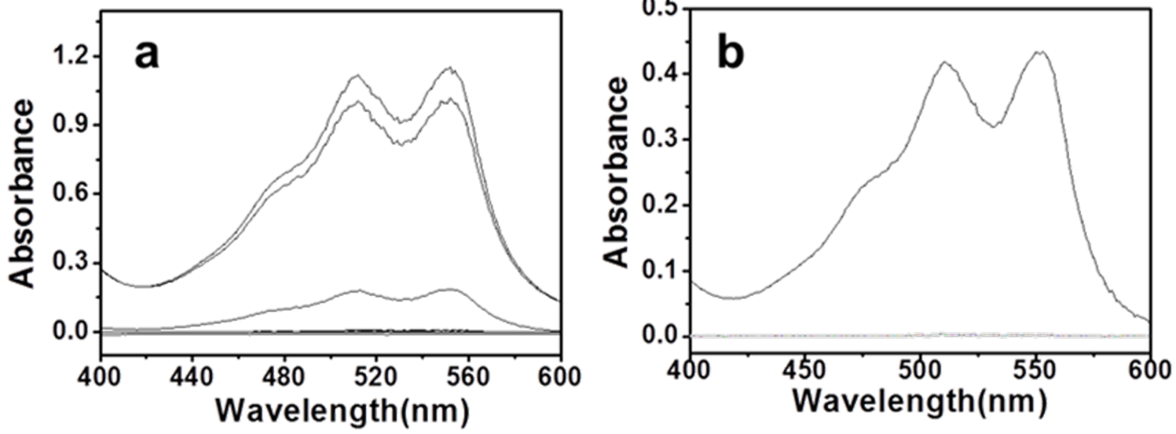


**Supplementary Figure 8** Uv-Vis absorption spectra of the standard solutions (a) and the control reaction (b).

**

**

**Supplementary Figure 9** B3PW91 thermodynamics between tetrahydroquinoline and 1H-pyrrole-2,5-dione.

**II. DFT Computational details**

Supplementary Table 1 B3PW91 optimized Cartesian Coordinates.

|  |  |
| --- | --- |
| H,0,-1.5414882137,0.142086892,-2.3838236648  C,0,-1.5373951148,0.1252319116,-1.2975050354  C,0,-1.5155529953,0.0713367771,1.4601107588  C,0,-0.3136432162,0.0390003281,-0.6417350468  C,0,-2.7398789001,0.1926153073,-0.6074305415  C,0,-2.7184619613,0.1671578831,0.7827730901  C,0,-0.3010809256,0.0060966043,0.7643672681  H,0,-3.6783145314,0.2623832671,-1.1446489206  H,0,-3.6446536317,0.2181004701,1.3449592637  H,0,-1.5049431182,0.0396451563,2.5457920892  N,0,0.8898404982,-0.1406781846,1.4596413833  H,0,0.8504888245,0.1251185499,2.4302146763  C,0,0.9838830923,-0.0035127381,-1.4131206219  H,0,1.2433814931,1.0099048358,-1.7441366187  H,0,0.8528580171,-0.5956853235,-2.3234906463  C,0,2.1291975859,-0.5661834058,-0.5764324338  H,0,3.0860358596,-0.4107184928,-1.0811970178  H,0,2.0008633898,-1.6441304254,-0.439910684  C,0,2.1514241991,0.1004909023,0.7906536111  H,0,2.3547800572,1.1767347048,0.6677852826  H,0,2.9526595404,-0.3149950205,1.4071337544 | H,0,-1.4181628763,0.033271906,-2.2371842193  C,0,-1.381110692,0.0824584015,-1.1529380045  C,0,-1.2937985829,0.2140963149,1.6164145788  C,0,-0.1484757175,0.1233527068,-0.5152510477  C,0,-2.5633147834,0.0953157904,-0.4203770803  C,0,-2.5184289256,0.1587672879,0.9671665333  C,0,-0.1075949789,0.1917992065,0.8861757276  H,0,-3.5175888703,0.0565302618,-0.9338141534  H,0,-3.437501219,0.1706996459,1.5422384025  H,0,-1.2286590002,0.2744181758,2.6966437357  C,0,1.1604422644,0.1421781086,-1.2555491729  H,0,1.4541692161,1.1851491698,-1.4343342237  H,0,1.0576142466,-0.3276217265,-2.2361510253  C,0,2.2390206373,-0.5475815,-0.4275010088  H,0,3.2355800904,-0.3736682369,-0.8394197696  H,0,2.0844766647,-1.636028346,-0.4302432087  N,0,1.1065592293,0.2626732357,1.6018374196  C,0,2.1754652174,-0.0742073523,0.99841665  H,0,3.101308028,-0.03160305,1.5738698666 |
|  |  |
| H,0,1.8194266137,-2.2196592073,-0.9069768218  C,0,0.8601051426,-1.7184499892,-0.9924897264  C,0,-1.5798779827,-0.4147082336,-1.1908110396  C,0,0.3602880048,-1.0415819737,0.1165998376  C,0,0.1592924012,-1.7587588981,-2.1905940492  C,0,-1.0609014814,-1.0987551148,-2.2813569703  C,0,-0.8823274544,-0.3787568024,0.0179779402  H,0,0.5605733237,-2.2931849815,-3.0432644901  H,0,-1.6205831137,-1.1176025,-3.2102214854  H,0,-2.5326148231,0.0994591806,-1.2749192337  C,0,1.0782464033,-0.9439886171,1.3774176536  H,0,2.1043568934,-1.2972319853,1.4102035139  C,0,0.5031406545,-0.4335496454,2.4680079558  H,0,1.0373272891,-0.3740471848,3.409745518  N,0,-1.3330273585,0.3541056391,1.0977501782  H,0,-2.2699832978,0.713212754,1.0090356538  C,0,-0.928705554,0.0129998123,2.4516694104  H,0,-1.5652960092,-0.7792246815,2.8907345531  H,0,-1.0567696445,0.8976894337,3.0829404901 | H,0,2.3579309285,0.,-1.1772589665  C,0,1.2726554863,0.,-1.1647413209  C,0,-1.5274076198,0.,-1.1224980564  C,0,0.6020567449,0.,0.0798515712  C,0,0.5616034682,0.,-2.3358821966  C,0,-0.8491893203,0.,-2.3130994143  C,0,-0.8228910793,0.,0.1029635752  H,0,1.0814940694,0.,-3.2872743807  H,0,-1.3974900427,0.,-3.2485822652  H,0,-2.6101245648,0.,-1.0808353666  N,0,-1.5341945314,0.,1.2619181602  C,0,-0.8672797233,0.,2.3892250774  H,0,-1.4614228457,0.,3.300409868  C,0,1.273350493,0.,1.3226554278  H,0,2.3586218793,0.,1.3440248681  C,0,0.5421627507,0.,2.4783302426  H,0,1.0201323752,0.,3.4508202689 |
|   C,0,-0.2243212043,-0.0718304834,1.0528258025  H,0,-0.4161973756,0.8174340998,1.6425410552  C,0,0.3879876905,-1.2150962334,1.692676974  H,0,0.6431119176,-1.1440875453,2.7447134223  C,0,-0.5384599131,-0.1022416584,-0.2569094571  H,0,-0.9748258624,0.7624880443,-0.7470679286  C,0,0.6328463276,-2.3545066776,1.0167753768  H,0,1.0678172858,-3.2257343188,1.4920388245  C,0,-0.2594363638,-1.2733508644,-1.0589604908  C,0,0.2573923799,-2.4917071531,-0.3806356706  C,0,-0.3479834388,-1.287758862,-2.4047414497  H,0,-0.6465051363,-0.3921567255,-2.9431195398  N,0,0.3554207193,-3.6441878453,-0.9458136627  C,0,0.0743299675,-2.5038033218,-3.1599198895  H,0,1.1373666065,-2.4010876905,-3.4305732981  H,0,-0.4705776606,-2.5955304496,-4.1033139358  C,0,-0.1480909026,-3.7437548729,-2.3053652971  H,0,0.3004067673,-4.6213996227,-2.7757782493  H,0,-1.2291633044,-3.9478684194,-2.2373026862 | O2-Triplet  O,0,0.,0.,0.834371757  O,0,0.,0.,-0.366371757  H2O  H,0,0.,0.0004967663,-0.0000732467  O,0,0.,-0.0007050158,0.9605449511  H,0,0.,0.9274962495,1.207994294  H2O2  O,0,-0.1456779207,0.1056603005,0.9633472096  H,0,0.381594754,0.8552482316,1.2683044737  O,0,-0.0250422265,0.2669220776,-0.4653702631  H,0,-0.9537432531,0.3758907549,-0.7073159253 |
|  |  |
| H,0,0.0600533118,-0.0059137149,-0.0776568862  N,0,0.0168285638,-0.0023054076,0.9289611921  C,0,0.0198285196,1.151379403,1.7056390062  C,0,-0.0530931987,-1.1504037324,1.7107586419  C,0,-0.0564272114,0.6704189513,3.1239744201  H,0,-0.0706066498,1.3576552268,3.9574816874  C,0,-0.0985528709,-0.6592827456,3.1269319482  H,0,-0.1560866789,-1.3405325564,3.963483008  O,0,-0.0718075578,-2.2861628074,1.3122260867  O,0,0.0729859789,2.2842632434,1.3020605258 | H,0,0.5811406402,-0.3838932193,-2.0052964948  N,0,0.2441175745,-0.313638795,-1.059272378  C,0,0.0189236745,-1.4369489274,-0.2931254183  C,0,0.0113341103,0.3888634224,1.1103049708  H,0,-0.0644177725,1.0696634743,1.947697829  C,0,-0.1803362854,-0.9254116694,1.0978904944  H,0,-0.4352126856,-1.5802340144,1.9186526556  O,0,-0.0053808673,-2.5826946474,-0.6808359328  C,0,0.3449365823,0.8933860407,-0.276328982  H,0,1.3575403293,1.3098305411,-0.3096196084  O,0,-0.4813657137,1.9408879116,-0.7252832165  H,0,-1.3912796924,1.6201898829,-0.7247839718 |
|  |  |
| C,0,0.0122772827,-1.2584898236,1.9639661315  O,0,0.0633357941,-1.3425085456,3.1413571182  N,0,-0.0105467461,-2.4007646352,1.0604644553  H,0,0.0152458867,-3.3699233286,1.3589659701  O,0,-0.0996730817,-2.7858616935,-1.1647577638  H,0,-0.1423677295,-2.3536744744,-2.0335729909  C,0,-0.0423971197,-0.0678210428,1.0550833502  H,0,-0.0402039537,0.9458284768,1.4308704149  C,0,-0.0901671544,-0.4946904598,-0.2080754503  H,0,-0.1366576119,0.0873951012,-1.1184956583  C,0,-0.0689111525,-1.9657458865,-0.1770279811 | H,0,3.4661189904,1.4919179897,-1.9962678456  C,0,2.7963916296,1.5018594539,-1.1413578023  C,0,1.0569148747,1.5304648248,0.9968773876  C,0,1.7534964781,0.5873160697,-1.1140854551  C,0,3.0060148568,2.4122666979,-0.1130313472  C,0,2.1219405837,2.4187194963,0.9545644501  C,0,0.8620147068,0.5829215781,-0.0195467602  H,0,3.8342429025,3.1094077611,-0.1533599538  H,0,2.2445357341,3.1318811604,1.7624389906  H,0,0.3660049579,1.5997570936,1.827786116  N,0,-0.1788404143,-0.3396644126,0.0246431924  C,0,1.52753752,-0.380006309,-2.2406993927  H,0,2.0052500085,-1.3430285165,-2.0206149642  H,0,1.988157064,-0.0005675479,-3.1559392132  C,0,-0.5631412197,-1.1325152015,-1.1393213593  H,0,-1.6583728361,-1.1368624619,-1.2148050226  H,0,-0.2479766913,-2.1724379696,-0.9971928624  C,0,0.0310920458,-0.5927933975,-2.4263883545  H,0,-0.176026595,-1.3018102207,-3.2315731465  H,0,-0.442041564,0.3569706005,-2.6942527146  C,0,-0.9568085666,-0.521807903,1.2120941916  H,0,-0.3505707017,-0.2202172873,2.0708767532  C,0,-2.3255842665,0.1854370204,1.2773169564  H,0,-2.3247574156,1.1148869884,1.8464347018  H,0,-2.6939806901,0.4186937651,0.2749895142  C,0,-1.2870256026,-1.9971858581,1.513907687  O,0,-0.5586631973,-2.9496105713,1.4251328378  N,0,-2.5953061543,-2.0269108444,1.9636256386  H,0,-3.0296260707,-2.8719903421,2.3084112004  C,0,-3.271596509,-0.8147435709,1.9092237432  O,0,-4.3993937512,-0.6403480866,2.2901129934 |
|  |  |
| H,0,-1.3929967779,0.1209776001,-3.9076754289  C,0,-1.0748702677,0.7198852437,-3.0611272248  C,0,-0.2450229255,2.2629354672,-0.9200473099  C,0,-0.3600729311,0.0973551962,-2.0333473596  C,0,-1.3723649326,2.0705154421,-3.025958075  C,0,-0.9476084187,2.8517844192,-1.9557071052  C,0,0.0243266998,0.898617023,-0.9652980664  H,0,-1.9273278313,2.5217140645,-3.8397580381  H,0,-1.1568537203,3.9140122766,-1.9304039023  H,0,0.1037114544,2.86950208,-0.0896618683  C,0,0.0230495131,-1.3534127077,-2.1448546338  H,0,0.8278521171,-1.436645463,-2.8844234483  H,0,-0.8158546442,-1.9256237678,-2.5463588131  C,0,0.4798011241,-1.9423570191,-0.8199867048  H,0,1.0054582118,-2.8860924251,-0.9797685551  H,0,-0.3725190272,-2.1643175374,-0.1764831953  N,0,0.7751501729,0.3314635147,0.1818259699  H,0,1.549724931,0.9818131782,0.383834711  C,0,1.4249646713,-0.9997154037,-0.1091992766  H,0,2.2979772001,-0.7775009655,-0.7260811985  H,0,1.7701380013,-1.3982755532,0.8444826483  C,0,0.004727312,0.3715358359,1.5052756821  H,0,-0.576938029,1.2958821069,1.4562142062  C,0,-0.7998923064,-0.7865949113,1.9656485817  H,0,-1.7290642448,-1.1200042762,1.5312458384  C,0,-0.3654025962,-1.0853022318,3.2074817781  C,0,1.096017274,0.522885845,2.58806994  O,0,2.0826428676,1.2062711763,2.4681188032  N,0,0.7375999579,-0.3180074038,3.5955419802  H,0,1.2846824077,-0.4896645845,4.427640517  O,0,-0.7936073948,-1.9250739974,4.1342506586  H,0,-1.5574279766,-2.428562011,3.8265087323 | H,0,3.4898426509,1.126548941,-1.9835838472  C,0,2.869763264,1.207687495,-1.0966551301  C,0,1.2855437847,1.4073625456,1.1460434031  C,0,1.654900737,0.5305476611,-1.0798334675  C,0,3.3017494413,1.9792125848,-0.0293697686  C,0,2.5002203185,2.0730549223,1.0972471053  C,0,0.83410369,0.6473674375,0.0595117963  H,0,4.2535554411,2.4938935224,-0.0746899175  H,0,2.8199599541,2.6556071048,1.9530706631  H,0,0.7178477753,1.4802572542,2.0647386253  N,0,-0.4410057528,0.0289317776,0.0507596447  C,0,1.2576889931,-0.3012976214,-2.2736031424  H,0,1.8353698839,-1.2332142536,-2.2646352997  H,0,1.5398347836,0.2193088899,-3.1913377595  C,0,-0.6586574091,-1.0617701383,-0.8982427966  H,0,-1.719092952,-1.3211458205,-0.9051829381  H,0,-0.0984940147,-1.9625816825,-0.6039156935  C,0,-0.226602998,-0.6262317211,-2.2871212117  H,0,-0.4445796211,-1.4209623778,-3.0035331027  H,0,-0.8112058973,0.249965351,-2.5806759805  C,0,-1.3040077185,0.1268566726,1.1903394389  H,0,-1.0521297995,1.027143422,1.7492565257  C,0,-2.8131449521,0.0802260039,0.8915340468  H,0,-3.3550920506,0.8332288098,1.4700100566  H,0,-3.0536729554,0.2480707173,-0.1569274659  N,0,-2.1709662112,-1.8731740776,2.0675815468  H,0,-2.2592448943,-2.7936569776,2.4885390481  C,0,-3.294559569,-1.2754484723,1.3401446746  O,0,-4.326295201,-1.8385371991,1.2149365687  C,0,-1.1129061763,-1.0931342579,2.0701720751  O,0,0.0248291215,-1.3408326869,2.6267604712  H,0,0.097583334,-2.2096428262,3.0544358311 |
|   Singlet State |   Triplet state |
| H,0,3.1068851734,1.7575670478,-2.0509008765  C,0,2.6232416145,1.4701594215,-1.1238678126  C,0,1.4352695375,0.6892002758,1.2526690543  C,0,1.5297598755,0.6039487634,-1.1855798109  C,0,3.112667802,1.9394945925,0.0812574258  C,0,2.5286133428,1.5321492708,1.2771927114  C,0,0.9216900461,0.2688864234,0.0256098685  H,0,3.967283636,2.6054971109,0.0935483847  H,0,2.9310775545,1.8610869917,2.2271385155  H,0,1.0091166698,0.3380645261,2.1844415016  N,0,-0.2531548501,-0.6314454972,0.047489974  C,0,1.0822603325,0.0507047739,-2.5086905327  H,0,1.9581165479,-0.1368631761,-3.1320663591  H,0,0.4826498376,0.8012914864,-3.0379082403  C,0,-0.8183841175,-0.9449897246,-1.3094508016  H,0,-1.3948157731,-0.0675206609,-1.6073800781  H,0,-1.4903476754,-1.7894639565,-1.1602256485  C,0,0.2723217001,-1.2175706443,-2.31406718  H,0,0.9003665913,-2.0457807773,-1.9782331491  H,0,-0.2034602908,-1.5310585146,-3.2452801535  C,0,-1.3282643092,-0.2530810129,1.0860277989  H,0,-0.9419204082,0.5998070735,1.6301095439  C,0,-2.766941277,-0.0283759535,0.6396827751  H,0,-3.0801123563,1.0068949259,0.7689544563  H,0,-2.9904211993,-0.307798124,-0.3873078043  N,0,-2.7365309121,-1.7438235684,2.2456046893  H,0,-3.0733928511,-2.4037772339,2.9321164588  C,0,-3.6106889341,-0.9391108326,1.5370433403  O,0,-4.8027514588,-0.9628290704,1.6070632086  C,0,-1.3743416901,-1.4977374853,2.0099731816  O,0,-0.8567049903,-2.5369491493,1.1418184856  O,0,-0.5757083694,-1.3063929655,3.1079221188  H,0,-0.4566983264,-2.1320885689,3.5957500055  O,0,0.2842113276,-1.9181681671,0.5971220489 | H,0,3.3838276795,1.5021739442,-1.9048022548  C,0,2.7622598886,1.3439171209,-1.030802431  C,0,1.210611965,0.930355533,1.2525329284  C,0,1.5811068983,0.6436633446,-1.1739542127  C,0,3.1736064589,1.8383753218,0.2036598827  C,0,2.3928397433,1.6123352224,1.3473534761  C,0,0.7542003323,0.4474272326,-0.0111732951  H,0,4.1064129311,2.3839959498,0.2840441775  H,0,2.7301921253,1.9678080334,2.3131995372  H,0,0.6544563346,0.7196470945,2.1541118615  N,0,-0.4451072431,-0.1798426876,-0.1321818614  C,0,1.1566003508,0.1042577507,-2.5019159365  H,0,2.037227134,-0.1115760172,-3.1090601091  H,0,0.5896304552,0.8745913038,-3.0417961888  C,0,-0.8700037035,-0.7868008532,-1.4073652034  H,0,-1.5395502319,-0.0732388674,-1.902751281  H,0,-1.4488880297,-1.6764821635,-1.1635340842  C,0,0.2957892881,-1.1298079396,-2.3051417009  H,0,0.8817745457,-1.9413813556,-1.8648198371  H,0,-0.0978879528,-1.4935918722,-3.255401035  C,0,-1.4060351863,-0.2061725517,0.9682338408  H,0,-1.1836283532,0.6346784866,1.6219312828  C,0,-2.8886822405,-0.1247836215,0.6034823264  H,0,-3.2572653465,0.8956410233,0.5097836586  H,0,-3.1540874935,-0.6682680886,-0.3037014783  N,0,-2.5924754556,-1.5543856553,2.4319313971  H,0,-2.7994975548,-2.1307067197,3.2365716349  C,0,-3.5734523085,-0.835683988,1.7679858599  O,0,-4.7328228284,-0.797374093,2.0574813696  C,0,-1.3071009466,-1.4583378049,1.9019895839  O,0,-1.0824554828,-2.6938591796,0.9752445938  O,0,-0.3012446621,-1.4474352198,2.7958630405  H,0,0.3077039264,-2.1811316902,2.5341444494  O,0,0.0528363624,-3.2780794931,1.1964332083 |
|   Singlet state |   Triplet state |
| H,0,3.7260576546,0.9167190118,-1.6541475037  C,0,2.9479857236,1.0961231868,-0.9206990615  C,0,0.9494650133,1.5793255064,0.9479066655  C,0,1.7415798023,0.4206481162,-1.0391105908  C,0,3.1626420406,2.0009837543,0.1106684848  C,0,2.1578350731,2.2531262938,1.0342726005  C,0,0.7565421662,0.6529943261,-0.0720076164  H,0,4.1101301094,2.5211943427,0.1835503814  H,0,2.307896917,2.9789725234,1.8237792184  H,0,0.1712116036,1.8155075559,1.6617664809  N,0,-0.4890192659,-0.0718804146,-0.1931235768  C,0,1.4525967411,-0.5368072099,-2.1581701902  H,0,1.673981591,-1.563666194,-1.8456399723  H,0,2.0838061515,-0.3216641336,-3.02017381  C,0,-0.8622401556,-0.5404673377,-1.3381035016  C,0,-0.0189685257,-0.4174408881,-2.543311096  H,0,-0.3270809494,-1.1660134137,-3.2742592874  H,0,-0.2223850173,0.5656591401,-2.9961057169  C,0,-1.2832617501,-0.3587602691,1.0235091492  H,0,-0.8243798987,0.2051570931,1.8274355376  C,0,-2.7751247017,-0.066358977,0.9856066113  H,0,-3.0315261894,0.9587461459,1.2501834518  H,0,-3.2408194767,-0.2905793917,0.0231856017  N,0,-2.3354545112,-1.9396143475,2.3312597081  H,0,-2.5468509308,-2.7937231696,2.8302327747  C,0,-3.3449719229,-1.0483348222,2.0117801721  O,0,-4.4567700681,-1.0539918647,2.4569444009  C,0,-1.1811658602,-1.8502554673,1.4957625339  O,0,-1.2600678385,-2.6301315823,0.3227700081  O,0,0.0001489002,-2.158800351,2.1328981361  H,0,-0.1036272917,-2.0647337384,3.0862981864  O,0,-1.3468540672,-4.0103009332,0.7169743825  H,0,-2.1715396369,-4.2672330485,0.2778100313  H,0,-1.8122651294,-1.0577693424,-1.370442994 | H,0,3.41209713,1.4875235017,-1.9626278922  C,0,2.8090934073,1.2934402341,-1.0828481289  C,0,1.286975615,0.8349234433,1.2200517392  C,0,1.635551959,0.5776560406,-1.2238389598  C,0,3.2315295148,1.7673060272,0.1550190144  C,0,2.4639206624,1.5226875863,1.3085375447  C,0,0.8290281916,0.3599542162,-0.0448336453  H,0,4.1597641507,2.3211145292,0.2322517471  H,0,2.8127562039,1.8700317934,2.2733156462  H,0,0.7349570882,0.5977862451,2.1182606754  N,0,-0.3647413149,-0.2711961914,-0.1697149922  C,0,1.2133957777,0.0668535658,-2.5659259542  H,0,1.728300947,-0.882783697,-2.7545049915  H,0,1.5357066334,0.7605392903,-3.34537249  C,0,-0.6929806578,-0.9072522199,-1.4110305885  C,0,-0.3057182466,-0.1800360435,-2.6316390215  H,0,-0.5660124003,-0.7568324969,-3.5183593667  H,0,-0.8263497587,0.7827006197,-2.6941119511  C,0,-1.3638472016,-0.3018185932,0.8963422388  H,0,-1.1740538818,0.5385014654,1.5614473937  C,0,-2.8135675555,-0.2420291186,0.4226609819  H,0,-3.1814139194,0.7705397505,0.2664949808  H,0,-2.9747380021,-0.81391346,-0.4946980105  N,0,-2.655830832,-1.6528304845,2.2771499101  H,0,-2.900242282,-2.0825870083,3.1574281126  C,0,-3.5763656189,-0.9201920193,1.5578795042  O,0,-4.7498557286,-0.8349832688,1.788120415  C,0,-1.3101366679,-1.5617612435,1.8144162246  O,0,-0.942868992,-2.6456110442,0.9695028441  O,0,-0.3879473843,-1.3901281433,2.8253790909  H,0,-0.1344408199,-2.2611885403,3.163277021  O,0,-0.8521415158,-3.8111945383,1.801243256  H,0,-1.6528371447,-4.2972796079,1.5511720531  H,0,-0.8194810563,-1.9813104903,-1.3511449011 |
|  |  |
| C,0,-0.149202422,-1.148905925,1.8984161248  O,0,-0.3581492179,-1.2349042702,3.0549261731  N,0,-0.4822287086,-2.2224338278,0.9541421195  H,0,-0.8229567097,-3.1318535133,1.2583462607  C,0,-0.2143194125,-1.8914592863,-0.2774591691  O,0,-0.2909532747,-2.7044978924,-1.2593491871  H,0,0.0446633644,-2.262975026,-2.0773464556  O,0,1.371637214,-0.3213989886,-1.1638797638  O,0,0.913666712,-0.6668025459,-2.5005070152  H,0,1.6834581814,-0.4136550995,-3.034836752  C,0,0.2157414202,-0.4380455633,-0.3780501443  C,0,0.4922833457,-0.0535484909,1.0709655433  H,0,0.1034850615,0.9231492066,1.3533769075  H,0,1.568778601,-0.0640476512,1.2711827095  H,0,-0.607842255,0.1408087738,-0.8167696513 | C,0,0.1387463807,-1.235559242,1.8458083078  O,0,0.1900365411,-1.4439585324,3.0273359716  N,0,0.2533109063,-2.0875933113,0.7814395911  H,0,0.387299518,-3.0856433947,0.8958608024  C,0,0.1650370128,-1.4955338118,-0.5179288265  O,0,-0.9428551514,-1.9360428779,-1.1865017132  H,0,-0.821143844,-1.7683544982,-2.1345181655  O,0,1.3741452849,-1.8091279071,-1.1585362287  O,0,1.2630632093,-1.181003167,-2.4585156097  H,0,1.859313958,-1.7440168727,-2.9767807754  C,0,0.0746376166,0.039349543,-0.1798649947  H,0,1.0866452263,0.486711069,-0.3576719752  H,0,-0.5938205804,0.6730842121,-0.7768999172  C,0,-0.0973746138,0.1053935575,1.2178489748  H,0,-0.3730553485,0.9766565864,1.8111034964 |
|  |  |
| H,0,-1.5414882137,0.142086892,-2.3838236648  C,0,-1.5373951148,0.1252319116,-1.2975050354  C,0,-1.5155529953,0.0713367771,1.4601107588  C,0,-0.3136432162,0.0390003281,-0.6417350468  C,0,-2.7398789001,0.1926153073,-0.6074305415  C,0,-2.7184619613,0.1671578831,0.7827730901  C,0,-0.3010809256,0.0060966043,0.7643672681  H,0,-3.6783145314,0.2623832671,-1.1446489206  H,0,-3.6446536317,0.2181004701,1.3449592637  H,0,-1.5049431182,0.0396451563,2.5457920892  N,0,0.8898404982,-0.1406781846,1.4596413833  H,0,0.8504888245,0.1251185499,2.4302146763  C,0,0.9838830923,-0.0035127381,-1.4131206219  H,0,1.2433814931,1.0099048358,-1.7441366187  H,0,0.8528580171,-0.5956853235,-2.3234906463  C,0,2.1291975859,-0.5661834058,-0.5764324338  H,0,3.0860358596,-0.4107184928,-1.0811970178  H,0,2.0008633898,-1.6441304254,-0.439910684  C,0,2.1514241991,0.1004909023,0.7906536111  H,0,2.3547800572,1.1767347048,0.6677852826  H,0,2.9526595404,-0.3149950205,1.4071337544 | H,0,-1.4181628763,0.033271906,-2.2371842193  C,0,-1.381110692,0.0824584015,-1.1529380045  C,0,-1.2937985829,0.2140963149,1.6164145788  C,0,-0.1484757175,0.1233527068,-0.5152510477  C,0,-2.5633147834,0.0953157904,-0.4203770803  C,0,-2.5184289256,0.1587672879,0.9671665333  C,0,-0.1075949789,0.1917992065,0.8861757276  H,0,-3.5175888703,0.0565302618,-0.9338141534  H,0,-3.437501219,0.1706996459,1.5422384025  H,0,-1.2286590002,0.2744181758,2.6966437357  C,0,1.1604422644,0.1421781086,-1.2555491729  H,0,1.4541692161,1.1851491698,-1.4343342237  H,0,1.0576142466,-0.3276217265,-2.2361510253  C,0,2.2390206373,-0.5475815,-0.4275010088  H,0,3.2355800904,-0.3736682369,-0.8394197696  H,0,2.0844766647,-1.636028346,-0.4302432087  N,0,1.1065592293,0.2626732357,1.6018374196  C,0,2.1754652174,-0.0742073523,0.99841665  H,0,3.101308028,-0.03160305,1.5738698666 |
|  |  |
| H,0,1.8194266137,-2.2196592073,-0.9069768218  C,0,0.8601051426,-1.7184499892,-0.9924897264  C,0,-1.5798779827,-0.4147082336,-1.1908110396  C,0,0.3602880048,-1.0415819737,0.1165998376  C,0,0.1592924012,-1.7587588981,-2.1905940492  C,0,-1.0609014814,-1.0987551148,-2.2813569703  C,0,-0.8823274544,-0.3787568024,0.0179779402  H,0,0.5605733237,-2.2931849815,-3.0432644901  H,0,-1.6205831137,-1.1176025,-3.2102214854  H,0,-2.5326148231,0.0994591806,-1.2749192337  C,0,1.0782464033,-0.9439886171,1.3774176536  H,0,2.1043568934,-1.2972319853,1.4102035139  C,0,0.5031406545,-0.4335496454,2.4680079558  H,0,1.0373272891,-0.3740471848,3.409745518  N,0,-1.3330273585,0.3541056391,1.0977501782  H,0,-2.2699832978,0.713212754,1.0090356538  C,0,-0.928705554,0.0129998123,2.4516694104  H,0,-1.5652960092,-0.7792246815,2.8907345531  H,0,-1.0567696445,0.8976894337,3.0829404901 | H,0,2.3579309285,0.,-1.1772589665  C,0,1.2726554863,0.,-1.1647413209  C,0,-1.5274076198,0.,-1.1224980564  C,0,0.6020567449,0.,0.0798515712  C,0,0.5616034682,0.,-2.3358821966  C,0,-0.8491893203,0.,-2.3130994143  C,0,-0.8228910793,0.,0.1029635752  H,0,1.0814940694,0.,-3.2872743807  H,0,-1.3974900427,0.,-3.2485822652  H,0,-2.6101245648,0.,-1.0808353666  N,0,-1.5341945314,0.,1.2619181602  C,0,-0.8672797233,0.,2.3892250774  H,0,-1.4614228457,0.,3.300409868  C,0,1.273350493,0.,1.3226554278  H,0,2.3586218793,0.,1.3440248681  C,0,0.5421627507,0.,2.4783302426  H,0,1.0201323752,0.,3.4508202689 |
|   C,0,-0.2243212043,-0.0718304834,1.0528258025  H,0,-0.4161973756,0.8174340998,1.6425410552  C,0,0.3879876905,-1.2150962334,1.692676974  H,0,0.6431119176,-1.1440875453,2.7447134223  C,0,-0.5384599131,-0.1022416584,-0.2569094571  H,0,-0.9748258624,0.7624880443,-0.7470679286  C,0,0.6328463276,-2.3545066776,1.0167753768  H,0,1.0678172858,-3.2257343188,1.4920388245  C,0,-0.2594363638,-1.2733508644,-1.0589604908  C,0,0.2573923799,-2.4917071531,-0.3806356706  C,0,-0.3479834388,-1.287758862,-2.4047414497  H,0,-0.6465051363,-0.3921567255,-2.9431195398  N,0,0.3554207193,-3.6441878453,-0.9458136627  C,0,0.0743299675,-2.5038033218,-3.1599198895  H,0,1.1373666065,-2.4010876905,-3.4305732981  H,0,-0.4705776606,-2.5955304496,-4.1033139358  C,0,-0.1480909026,-3.7437548729,-2.3053652971  H,0,0.3004067673,-4.6213996227,-2.7757782493  H,0,-1.2291633044,-3.9478684194,-2.2373026862 | O2-Triplet  O,0,0.,0.,0.834371757  O,0,0.,0.,-0.366371757  H2O  H,0,0.,0.0004967663,-0.0000732467  O,0,0.,-0.0007050158,0.9605449511  H,0,0.,0.9274962495,1.207994294  H2O2  O,0,-0.1456779207,0.1056603005,0.9633472096  H,0,0.381594754,0.8552482316,1.2683044737  O,0,-0.0250422265,0.2669220776,-0.4653702631  H,0,-0.9537432531,0.3758907549,-0.7073159253 |
|  |  |
| H,0,0.0600533118,-0.0059137149,-0.0776568862  N,0,0.0168285638,-0.0023054076,0.9289611921  C,0,0.0198285196,1.151379403,1.7056390062  C,0,-0.0530931987,-1.1504037324,1.7107586419  C,0,-0.0564272114,0.6704189513,3.1239744201  H,0,-0.0706066498,1.3576552268,3.9574816874  C,0,-0.0985528709,-0.6592827456,3.1269319482  H,0,-0.1560866789,-1.3405325564,3.963483008  O,0,-0.0718075578,-2.2861628074,1.3122260867  O,0,0.0729859789,2.2842632434,1.3020605258 | H,0,0.5811406402,-0.3838932193,-2.0052964948  N,0,0.2441175745,-0.313638795,-1.059272378  C,0,0.0189236745,-1.4369489274,-0.2931254183  C,0,0.0113341103,0.3888634224,1.1103049708  H,0,-0.0644177725,1.0696634743,1.947697829  C,0,-0.1803362854,-0.9254116694,1.0978904944  H,0,-0.4352126856,-1.5802340144,1.9186526556  O,0,-0.0053808673,-2.5826946474,-0.6808359328  C,0,0.3449365823,0.8933860407,-0.276328982  H,0,1.3575403293,1.3098305411,-0.3096196084  O,0,-0.4813657137,1.9408879116,-0.7252832165  H,0,-1.3912796924,1.6201898829,-0.7247839718 |
|  |  |
| C,0,0.0122772827,-1.2584898236,1.9639661315  O,0,0.0633357941,-1.3425085456,3.1413571182  N,0,-0.0105467461,-2.4007646352,1.0604644553  H,0,0.0152458867,-3.3699233286,1.3589659701  O,0,-0.0996730817,-2.7858616935,-1.1647577638  H,0,-0.1423677295,-2.3536744744,-2.0335729909  C,0,-0.0423971197,-0.0678210428,1.0550833502  H,0,-0.0402039537,0.9458284768,1.4308704149  C,0,-0.0901671544,-0.4946904598,-0.2080754503  H,0,-0.1366576119,0.0873951012,-1.1184956583  C,0,-0.0689111525,-1.9657458865,-0.1770279811 | H,0,3.4661189904,1.4919179897,-1.9962678456  C,0,2.7963916296,1.5018594539,-1.1413578023  C,0,1.0569148747,1.5304648248,0.9968773876  C,0,1.7534964781,0.5873160697,-1.1140854551  C,0,3.0060148568,2.4122666979,-0.1130313472  C,0,2.1219405837,2.4187194963,0.9545644501  C,0,0.8620147068,0.5829215781,-0.0195467602  H,0,3.8342429025,3.1094077611,-0.1533599538  H,0,2.2445357341,3.1318811604,1.7624389906  H,0,0.3660049579,1.5997570936,1.827786116  N,0,-0.1788404143,-0.3396644126,0.0246431924  C,0,1.52753752,-0.380006309,-2.2406993927  H,0,2.0052500085,-1.3430285165,-2.0206149642  H,0,1.988157064,-0.0005675479,-3.1559392132  C,0,-0.5631412197,-1.1325152015,-1.1393213593  H,0,-1.6583728361,-1.1368624619,-1.2148050226  H,0,-0.2479766913,-2.1724379696,-0.9971928624  C,0,0.0310920458,-0.5927933975,-2.4263883545  H,0,-0.176026595,-1.3018102207,-3.2315731465  H,0,-0.442041564,0.3569706005,-2.6942527146  C,0,-0.9568085666,-0.521807903,1.2120941916  H,0,-0.3505707017,-0.2202172873,2.0708767532  C,0,-2.3255842665,0.1854370204,1.2773169564  H,0,-2.3247574156,1.1148869884,1.8464347018  H,0,-2.6939806901,0.4186937651,0.2749895142  C,0,-1.2870256026,-1.9971858581,1.513907687  O,0,-0.5586631973,-2.9496105713,1.4251328378  N,0,-2.5953061543,-2.0269108444,1.9636256386  H,0,-3.0296260707,-2.8719903421,2.3084112004  C,0,-3.271596509,-0.8147435709,1.9092237432  O,0,-4.3993937512,-0.6403480866,2.2901129934 |
|  |  |
| H,0,-1.3929967779,0.1209776001,-3.9076754289  C,0,-1.0748702677,0.7198852437,-3.0611272248  C,0,-0.2450229255,2.2629354672,-0.9200473099  C,0,-0.3600729311,0.0973551962,-2.0333473596  C,0,-1.3723649326,2.0705154421,-3.025958075  C,0,-0.9476084187,2.8517844192,-1.9557071052  C,0,0.0243266998,0.898617023,-0.9652980664  H,0,-1.9273278313,2.5217140645,-3.8397580381  H,0,-1.1568537203,3.9140122766,-1.9304039023  H,0,0.1037114544,2.86950208,-0.0896618683  C,0,0.0230495131,-1.3534127077,-2.1448546338  H,0,0.8278521171,-1.436645463,-2.8844234483  H,0,-0.8158546442,-1.9256237678,-2.5463588131  C,0,0.4798011241,-1.9423570191,-0.8199867048  H,0,1.0054582118,-2.8860924251,-0.9797685551  H,0,-0.3725190272,-2.1643175374,-0.1764831953  N,0,0.7751501729,0.3314635147,0.1818259699  H,0,1.549724931,0.9818131782,0.383834711  C,0,1.4249646713,-0.9997154037,-0.1091992766  H,0,2.2979772001,-0.7775009655,-0.7260811985  H,0,1.7701380013,-1.3982755532,0.8444826483  C,0,0.004727312,0.3715358359,1.5052756821  H,0,-0.576938029,1.2958821069,1.4562142062  C,0,-0.7998923064,-0.7865949113,1.9656485817  H,0,-1.7290642448,-1.1200042762,1.5312458384  C,0,-0.3654025962,-1.0853022318,3.2074817781  C,0,1.096017274,0.522885845,2.58806994  O,0,2.0826428676,1.2062711763,2.4681188032  N,0,0.7375999579,-0.3180074038,3.5955419802  H,0,1.2846824077,-0.4896645845,4.427640517  O,0,-0.7936073948,-1.9250739974,4.1342506586  H,0,-1.5574279766,-2.428562011,3.8265087323 | H,0,3.4898426509,1.126548941,-1.9835838472  C,0,2.869763264,1.207687495,-1.0966551301  C,0,1.2855437847,1.4073625456,1.1460434031  C,0,1.654900737,0.5305476611,-1.0798334675  C,0,3.3017494413,1.9792125848,-0.0293697686  C,0,2.5002203185,2.0730549223,1.0972471053  C,0,0.83410369,0.6473674375,0.0595117963  H,0,4.2535554411,2.4938935224,-0.0746899175  H,0,2.8199599541,2.6556071048,1.9530706631  H,0,0.7178477753,1.4802572542,2.0647386253  N,0,-0.4410057528,0.0289317776,0.0507596447  C,0,1.2576889931,-0.3012976214,-2.2736031424  H,0,1.8353698839,-1.2332142536,-2.2646352997  H,0,1.5398347836,0.2193088899,-3.1913377595  C,0,-0.6586574091,-1.0617701383,-0.8982427966  H,0,-1.719092952,-1.3211458205,-0.9051829381  H,0,-0.0984940147,-1.9625816825,-0.6039156935  C,0,-0.226602998,-0.6262317211,-2.2871212117  H,0,-0.4445796211,-1.4209623778,-3.0035331027  H,0,-0.8112058973,0.249965351,-2.5806759805  C,0,-1.3040077185,0.1268566726,1.1903394389  H,0,-1.0521297995,1.027143422,1.7492565257  C,0,-2.8131449521,0.0802260039,0.8915340468  H,0,-3.3550920506,0.8332288098,1.4700100566  H,0,-3.0536729554,0.2480707173,-0.1569274659  N,0,-2.1709662112,-1.8731740776,2.0675815468  H,0,-2.2592448943,-2.7936569776,2.4885390481  C,0,-3.294559569,-1.2754484723,1.3401446746  O,0,-4.326295201,-1.8385371991,1.2149365687  C,0,-1.1129061763,-1.0931342579,2.0701720751  O,0,0.0248291215,-1.3408326869,2.6267604712  H,0,0.097583334,-2.2096428262,3.0544358311 |
|   Singlet State |   Triplet state |
| H,0,3.1068851734,1.7575670478,-2.0509008765  C,0,2.6232416145,1.4701594215,-1.1238678126  C,0,1.4352695375,0.6892002758,1.2526690543  C,0,1.5297598755,0.6039487634,-1.1855798109  C,0,3.112667802,1.9394945925,0.0812574258  C,0,2.5286133428,1.5321492708,1.2771927114  C,0,0.9216900461,0.2688864234,0.0256098685  H,0,3.967283636,2.6054971109,0.0935483847  H,0,2.9310775545,1.8610869917,2.2271385155  H,0,1.0091166698,0.3380645261,2.1844415016  N,0,-0.2531548501,-0.6314454972,0.047489974  C,0,1.0822603325,0.0507047739,-2.5086905327  H,0,1.9581165479,-0.1368631761,-3.1320663591  H,0,0.4826498376,0.8012914864,-3.0379082403  C,0,-0.8183841175,-0.9449897246,-1.3094508016  H,0,-1.3948157731,-0.0675206609,-1.6073800781  H,0,-1.4903476754,-1.7894639565,-1.1602256485  C,0,0.2723217001,-1.2175706443,-2.31406718  H,0,0.9003665913,-2.0457807773,-1.9782331491  H,0,-0.2034602908,-1.5310585146,-3.2452801535  C,0,-1.3282643092,-0.2530810129,1.0860277989  H,0,-0.9419204082,0.5998070735,1.6301095439  C,0,-2.766941277,-0.0283759535,0.6396827751  H,0,-3.0801123563,1.0068949259,0.7689544563  H,0,-2.9904211993,-0.307798124,-0.3873078043  N,0,-2.7365309121,-1.7438235684,2.2456046893  H,0,-3.0733928511,-2.4037772339,2.9321164588  C,0,-3.6106889341,-0.9391108326,1.5370433403  O,0,-4.8027514588,-0.9628290704,1.6070632086  C,0,-1.3743416901,-1.4977374853,2.0099731816  O,0,-0.8567049903,-2.5369491493,1.1418184856  O,0,-0.5757083694,-1.3063929655,3.1079221188  H,0,-0.4566983264,-2.1320885689,3.5957500055  O,0,0.2842113276,-1.9181681671,0.5971220489 | H,0,3.3838276795,1.5021739442,-1.9048022548  C,0,2.7622598886,1.3439171209,-1.030802431  C,0,1.210611965,0.930355533,1.2525329284  C,0,1.5811068983,0.6436633446,-1.1739542127  C,0,3.1736064589,1.8383753218,0.2036598827  C,0,2.3928397433,1.6123352224,1.3473534761  C,0,0.7542003323,0.4474272326,-0.0111732951  H,0,4.1064129311,2.3839959498,0.2840441775  H,0,2.7301921253,1.9678080334,2.3131995372  H,0,0.6544563346,0.7196470945,2.1541118615  N,0,-0.4451072431,-0.1798426876,-0.1321818614  C,0,1.1566003508,0.1042577507,-2.5019159365  H,0,2.037227134,-0.1115760172,-3.1090601091  H,0,0.5896304552,0.8745913038,-3.0417961888  C,0,-0.8700037035,-0.7868008532,-1.4073652034  H,0,-1.5395502319,-0.0732388674,-1.902751281  H,0,-1.4488880297,-1.6764821635,-1.1635340842  C,0,0.2957892881,-1.1298079396,-2.3051417009  H,0,0.8817745457,-1.9413813556,-1.8648198371  H,0,-0.0978879528,-1.4935918722,-3.255401035  C,0,-1.4060351863,-0.2061725517,0.9682338408  H,0,-1.1836283532,0.6346784866,1.6219312828  C,0,-2.8886822405,-0.1247836215,0.6034823264  H,0,-3.2572653465,0.8956410233,0.5097836586  H,0,-3.1540874935,-0.6682680886,-0.3037014783  N,0,-2.5924754556,-1.5543856553,2.4319313971  H,0,-2.7994975548,-2.1307067197,3.2365716349  C,0,-3.5734523085,-0.835683988,1.7679858599  O,0,-4.7328228284,-0.797374093,2.0574813696  C,0,-1.3071009466,-1.4583378049,1.9019895839  O,0,-1.0824554828,-2.6938591796,0.9752445938  O,0,-0.3012446621,-1.4474352198,2.7958630405  H,0,0.3077039264,-2.1811316902,2.5341444494  O,0,0.0528363624,-3.2780794931,1.1964332083 |
|   Singlet state |   Triplet state |
| H,0,3.7260576546,0.9167190118,-1.6541475037  C,0,2.9479857236,1.0961231868,-0.9206990615  C,0,0.9494650133,1.5793255064,0.9479066655  C,0,1.7415798023,0.4206481162,-1.0391105908  C,0,3.1626420406,2.0009837543,0.1106684848  C,0,2.1578350731,2.2531262938,1.0342726005  C,0,0.7565421662,0.6529943261,-0.0720076164  H,0,4.1101301094,2.5211943427,0.1835503814  H,0,2.307896917,2.9789725234,1.8237792184  H,0,0.1712116036,1.8155075559,1.6617664809  N,0,-0.4890192659,-0.0718804146,-0.1931235768  C,0,1.4525967411,-0.5368072099,-2.1581701902  H,0,1.673981591,-1.563666194,-1.8456399723  H,0,2.0838061515,-0.3216641336,-3.02017381  C,0,-0.8622401556,-0.5404673377,-1.3381035016  C,0,-0.0189685257,-0.4174408881,-2.543311096  H,0,-0.3270809494,-1.1660134137,-3.2742592874  H,0,-0.2223850173,0.5656591401,-2.9961057169  C,0,-1.2832617501,-0.3587602691,1.0235091492  H,0,-0.8243798987,0.2051570931,1.8274355376  C,0,-2.7751247017,-0.066358977,0.9856066113  H,0,-3.0315261894,0.9587461459,1.2501834518  H,0,-3.2408194767,-0.2905793917,0.0231856017  N,0,-2.3354545112,-1.9396143475,2.3312597081  H,0,-2.5468509308,-2.7937231696,2.8302327747  C,0,-3.3449719229,-1.0483348222,2.0117801721  O,0,-4.4567700681,-1.0539918647,2.4569444009  C,0,-1.1811658602,-1.8502554673,1.4957625339  O,0,-1.2600678385,-2.6301315823,0.3227700081  O,0,0.0001489002,-2.158800351,2.1328981361  H,0,-0.1036272917,-2.0647337384,3.0862981864  O,0,-1.3468540672,-4.0103009332,0.7169743825  H,0,-2.1715396369,-4.2672330485,0.2778100313  H,0,-1.8122651294,-1.0577693424,-1.370442994 | H,0,3.41209713,1.4875235017,-1.9626278922  C,0,2.8090934073,1.2934402341,-1.0828481289  C,0,1.286975615,0.8349234433,1.2200517392  C,0,1.635551959,0.5776560406,-1.2238389598  C,0,3.2315295148,1.7673060272,0.1550190144  C,0,2.4639206624,1.5226875863,1.3085375447  C,0,0.8290281916,0.3599542162,-0.0448336453  H,0,4.1597641507,2.3211145292,0.2322517471  H,0,2.8127562039,1.8700317934,2.2733156462  H,0,0.7349570882,0.5977862451,2.1182606754  N,0,-0.3647413149,-0.2711961914,-0.1697149922  C,0,1.2133957777,0.0668535658,-2.5659259542  H,0,1.728300947,-0.882783697,-2.7545049915  H,0,1.5357066334,0.7605392903,-3.34537249  C,0,-0.6929806578,-0.9072522199,-1.4110305885  C,0,-0.3057182466,-0.1800360435,-2.6316390215  H,0,-0.5660124003,-0.7568324969,-3.5183593667  H,0,-0.8263497587,0.7827006197,-2.6941119511  C,0,-1.3638472016,-0.3018185932,0.8963422388  H,0,-1.1740538818,0.5385014654,1.5614473937  C,0,-2.8135675555,-0.2420291186,0.4226609819  H,0,-3.1814139194,0.7705397505,0.2664949808  H,0,-2.9747380021,-0.81391346,-0.4946980105  N,0,-2.655830832,-1.6528304845,2.2771499101  H,0,-2.900242282,-2.0825870083,3.1574281126  C,0,-3.5763656189,-0.9201920193,1.5578795042  O,0,-4.7498557286,-0.8349832688,1.788120415  C,0,-1.3101366679,-1.5617612435,1.8144162246  O,0,-0.942868992,-2.6456110442,0.9695028441  O,0,-0.3879473843,-1.3901281433,2.8253790909  H,0,-0.1344408199,-2.2611885403,3.163277021  O,0,-0.8521415158,-3.8111945383,1.801243256  H,0,-1.6528371447,-4.2972796079,1.5511720531  H,0,-0.8194810563,-1.9813104903,-1.3511449011 |
|  |  |
| C,0,-0.149202422,-1.148905925,1.8984161248  O,0,-0.3581492179,-1.2349042702,3.0549261731  N,0,-0.4822287086,-2.2224338278,0.9541421195  H,0,-0.8229567097,-3.1318535133,1.2583462607  C,0,-0.2143194125,-1.8914592863,-0.2774591691  O,0,-0.2909532747,-2.7044978924,-1.2593491871  H,0,0.0446633644,-2.262975026,-2.0773464556  O,0,1.371637214,-0.3213989886,-1.1638797638  O,0,0.913666712,-0.6668025459,-2.5005070152  H,0,1.6834581814,-0.4136550995,-3.034836752  C,0,0.2157414202,-0.4380455633,-0.3780501443  C,0,0.4922833457,-0.0535484909,1.0709655433  H,0,0.1034850615,0.9231492066,1.3533769075  H,0,1.568778601,-0.0640476512,1.2711827095  H,0,-0.607842255,0.1408087738,-0.8167696513 | C,0,0.1387463807,-1.235559242,1.8458083078  O,0,0.1900365411,-1.4439585324,3.0273359716  N,0,0.2533109063,-2.0875933113,0.7814395911  H,0,0.387299518,-3.0856433947,0.8958608024  C,0,0.1650370128,-1.4955338118,-0.5179288265  O,0,-0.9428551514,-1.9360428779,-1.1865017132  H,0,-0.821143844,-1.7683544982,-2.1345181655  O,0,1.3741452849,-1.8091279071,-1.1585362287  O,0,1.2630632093,-1.181003167,-2.4585156097  H,0,1.859313958,-1.7440168727,-2.9767807754  C,0,0.0746376166,0.039349543,-0.1798649947  H,0,1.0866452263,0.486711069,-0.3576719752  H,0,-0.5938205804,0.6730842121,-0.7768999172  C,0,-0.0973746138,0.1053935575,1.2178489748  H,0,-0.3730553485,0.9766565864,1.8111034964 |

Supplementary Table 2 B3PW91 computed energetic data [total electronic energy, E_tot_, in Hartree; Zero-point energy, ZPE, in kcal/mol; number of imaginary frequencies, NImag; as well as sum of electronic and thermal enthalpy, H_tot_, in Hartree, and sum of electronic and thermal free energy, G_tot_, in Hartree).

|  | E_tot_=-404,3178873  ZPE=114,09830  NImag=0 | H_tot_=-404,127140  G_tot_= -404,168524 |
| --- | --- | --- |
|  | E_tot_=-403,0919357  ZPE=99,11662  NImag=0 | H_tot_=-402,925675  G_tot_= -402,965857 |
|  | E_tot_=-401,8952482  ZPE=84,82509  NImag=0 | H_tot_=-401,752362  G_tot_= -401,791267 |
|  | E_tot_=-403,0881373  ZPE=99,18848  NImag=0 | H_tot_=-402,921520  G_tot_= -402,962158 |
|  | E_tot_=-403.0522151  ZPE=98.67151  NImag=0 | H_tot_=-402.886501  G_tot_=-402,926919 |
| O_2_-Triplet | E_tot_=-150,3222601  ZPE=2,37844  NImag=0 | H_tot_=-150,315163  G_tot_= -150,338427 |
| H_2_O | E_tot_=-76,4309618  ZPE=13,40154  NImag=0 | H_tot_=-76,405825  G_tot_= -76,427244 |
| H_2_O_2_ | E_tot_=-151.5512043  ZPE=16.69142  NImag=0 | H_tot_=-151.520418  G_tot_= -151,546254 |
|  | E_tot_=-359,4167242  ZPE=42,75284  NImag=0 | H_tot_=-359,342160  G_tot_= -359,377277 |
|  | E_tot_ =-763.7567474  ZPE= 159.58174  NImag=0 | H_tot_=-763.487511  G_tot_= -763,544773 |
|  | E_tot_=-360,6140646  ZPE=57,55050  NImag=0 | H_tot_=-360,515381  G_tot_= -360,552187 |
|  | E_tot_=-359.7311796  ZPE=50.54978  NImag=0 | H_tot_=-359.644022  G_tot_= -359,679991 |
|  | E_tot_=-764.095968  ZPE=168.58324  NImag=0 | H_tot_=-763.812478  G_tot_= -763,868321 |
|  | E_tot_=-764.0821811  ZPE=167.16690  NImag=0 | H_tot_=-763.800661  G_tot_= -763,857862 |
|  | Singlet state  E_tot_=-914.3997607  ZPE=173.10556  NImag=0 | H_tot_=-914.107889  G_tot_= -914,165620 |
|  | Triplet state  E_tot_=-914.4033131  ZPE=171.23303  NImag=0 | H_tot_=-914.113319  G_tot_= -914,175410 |
|  | Singlet state  E_tot_=-914.450505  ZPE=171.92192  NImag=0 | H_tot_= -914.158922  G_tot_= -914,221179 |
|  | Triplet state  E_tot_=-914.3785903  ZPE=169.68402  NImag=0 | H_tot_=-914.090235  G_tot_= -914,153696 |
|  | E_tot_=-511.3242959  ZPE= 70.59269  NImag=0 | H_tot_= -511.202657  G_tot_= -511,244788 |
|  | E_tot_=-511.2233489  ZPE= 66.73019  NImag=0 | H_tot_= -511.106915  G_tot_= -511.151498 |

**III. NMR data of products**

(**1, 95%** ) Substrate: 63.6 mg, Product: 60.4 mg.^1^H NMR (400.1 MHz, CDCl_3_) δ 8.96 – 8.89 (m, 1H), 8.13 (m, *J* = 11.0, 9.1 Hz, 2H), 7.81 (d, *J* = 8.1 Hz, 1H), 7.75 – 7.68 (m, 1H), 7.54 (t, *J* = 7.5 Hz, 1H), 7.39 (m, *J* = 8.2, 4.2 Hz, 1H). ^13^C NMR (101 MHz, CDCl_3_) δ 150.43, 148.33, 136.05, 129.50, 129.46, 128.30, 127.80, 126.55, 121.08.[^1^](#_ENREF_1)

(**2, 92%** ), Substrate: 72.4 mg, Product: 66.6 mg. ^1^H NMR (400.1 MHz, CDCl_3_) δ 8.79 (d, *J* = 4.0 Hz, 1H), 8.06 (d, *J* = 8.2 Hz, 1H), 8.01 (d, *J* = 8.5 Hz, 1H), 7.51 (d, *J* = 10.7 Hz, 2H), 7.34 (m, *J* = 8.2, 4.2 Hz, 1H), 2.47 (s, 3H). ^13^C NMR (101 MHz, CDCl_3_) δ 148.67, 145.76, 136.86, 136.36, 132.27, 128.35, 128.23, 126.63, 121.06, 21.58.[^2^](#_ENREF_2)

(**3, 91%**) Substrate: 70.8 mg, Product: 64.4 mg. ^1^H NMR (400.1 MHz, CDCl_3_) δ 8.29 (d, *J* = 8.5 Hz, 1H), 8.22 (d, *J* = 8.4 Hz, 1H), 7.82 (d, *J* = 8.1 Hz, 1H), 7.73 (t, *J* = 7.2 Hz, 1H), 7.54 (t, *J* = 7.4 Hz, 1H), 7.36 (d, *J* = 8.4 Hz, 1H), 2.85 (s, 3H). ^13^C NMR (101 MHz, CDCl_3_) δ 158.25, 144.43, 139.24, 131.15, 127.72, 127.00, 126.61, 126.06, 122.35, 23.64.[^3^](#_ENREF_3)

(**4, 76%**) Substrate: 81.3 mg, Product: 61.8 mg. ^1^H NMR (400.1 MHz, CDCl_3_) δ 8.74 (d, *J* = 3.1 Hz, 1H), 8.07 (m, *J* = 20.3, 8.7 Hz, 2H), 7.46 – 7.30 (m, 2H), 7.07 (d, *J* = 2.7 Hz, 1H), 3.91 (s, 3H). ^13^C NMR (101 MHz, CDCl_3_) δ 157.97, 147.08, 143.37, 135.70, 130.05, 129.45, 122.87, 121.38, 105.12, 55.60.[^2^](#_ENREF_2)^,^[^4^](#_ENREF_4)

(**5, 96%**) Substrate: 72.3 mg, Product: 69.4 mg. ^1^H NMR (400.1 MHz, CDCl_3_) δ 8.87 (m, *J* = 4.2, 1.3 Hz, 1H), 8.13 (m, *J* = 8.9, 5.3 Hz, 2H), 7.56 – 7.37 (m, 3H). ^13^C NMR (101 MHz, CDCl_3_) δ 161.70, 159.23, 149.28 (*J* = 3.03 MHz), 136.02 (*J* = 6.06 MHz), 131.54 (*J* = 9.09 MHz), 128.96 (*J* = 10.1 MHz), 121.83, 120.09 (*J* = 26.26 MHz), 110.79 (*J* = 21.21MHz).

(**6, 83%**) Substrate: 96.3 mg, Product: 79.9 mg. ^1^H NMR (400.1 MHz, CDCl_3_) δ 8.94 (d, *J* = 3.3 Hz, 1H), 8.13 (d, *J* = 8.2 Hz, 1H), 8.05 (d, *J* = 9.0 Hz, 1H), 7.98 (d, *J* = 2.0 Hz, 1H), 7.79 (m, *J* = 9.0, 2.1 Hz, 1H), 7.47 (m, *J* = 8.3, 4.2 Hz, 1H). ^13^C NMR (101 MHz, CDCl_3_) δ 149.86, 145.57, 136.17, 133.52, 130.25, 129.86, 129.36, 121.96, 120.95.[^1^](#_ENREF_1)

(**7, 93%**) Substrate: 70.0 mg, Product: 65.1 mg. ^1^H NMR (400.1 MHz, CD_3_OD) δ 8.61 (m, *J* = 4.4, 1.2 Hz, 1H), 8.19 (d, *J* = 8.3 Hz, 1H), 7.87 (d, *J* = 9.1 Hz, 1H), 7.44 (m, *J* = 8.4, 4.4 Hz, 1H), 7.37 (m, *J* = 9.1, 2.6 Hz, 1H), 7.15 (d, *J* = 2.6 Hz, 1H). ^13^C NMR (101 MHz, CD_3_OD) δ 157.74, 147.06, 142.59, 137.92, 131.55, 129.35, 124.29, 122.59, 109.74.[^1^](#_ENREF_1)

(**8, 89%**) Substrate: 74.6 mg, Product: 66.4 mg. ^1^H NMR (400.1 MHz, CD_3_OD) δ 8.58 (m, *J* = 4.8, 1.6 Hz, 1H), 8.27 (d, *J* = 8.2 Hz, 1H), 7.73 (d, *J* = 8.9 Hz, 1H), 7.27 (m, *J* = 8.2, 4.8 Hz, 1H), 7.19 – 7.04 (m, 2H). ^13^C NMR (101 MHz, CD_3_OD) δ 162.19, 149.06, 148.03, 140.92, 131.17, 124.88, 121.83, 119.47, 107.71.

(**9, 85%**) Substrate: 75.0 mg, Product: 63.8 mg. ^1^H NMR (400.1 MHz, CDCl_3_) δ 8.79 – 8.70 (m, 1H), 8.14 (d, *J* = 8.3 Hz, 1H), 7.45 – 7.38 (m, 2H), 7.31 (d, *J* = 8.1 Hz, 1H), 7.19 (d, *J* = 7.6 Hz, 1H). ^13^C NMR (101 MHz, CDCl_3_) δ 152.16, 147.80, 138.09, 136.37, 128.57, 127.82, 121.81, 117.92, 110.29.[^1^](#_ENREF_1)

(**10, 90%**) Substrate: 77.0 mg, Product: 69.3 mg. ^1^H NMR (400.1 MHz, CDCl_3_) δ 8.11 (m, *J* = 9.2, 5.2 Hz, 1H), 8.04 (d, *J* = 8.5 Hz, 1H), 7.48 – 7.41 (m, 1H), 7.38 (m, *J* = 8.7, 2.8 Hz, 1H), 7.31 (d, *J* = 8.5 Hz, 1H), 2.75 (s, 3H). ^13^C NMR (101 MHz, CDCl_3_) δ 161.39, 158.92, 158.03 (*J* = 2.02 MHz), 136.77 (*J* = 5.05 MHz), 129.98 (*J* = 9.09 MHz), 127.57 (*J* = 10.1 MHz), 122.95, 120.18 (*J* = 25.25 MHz), 110.70 (*J* = 22.22 MHz), 24.45.

(**11, 79%**) Substrate: 58.0 mg, Product: 45.8 mg. ^1^H NMR (400.1 MHz, CDCl_3_) δ 8.35 (s, 1H), 7.36 (m, *J* = 7.0, 2.2 Hz, 1H), 7.31 – 7.27 (m, 2H), 7.17 (d, *J* = 7.2 Hz, 1H), 3.78 (t, *J* = 7.0 Hz, 2H), 2.85 – 2.67 (m, 2H). ^13^C NMR (101 MHz, CDCl_3_) δ 160.49, 136.36, 131.20, 127.46, 127.34, 127.13, 47.25, 25.03.

(**12, 87%**) Substrate: 72.7 mg, Product: 63.2 mg. ^1^H NMR (400.1 MHz, CD_3_OD) δ 8.06 (s, 1H), 7.19 (d, *J* = 8.5 Hz, 1H), 6.43 (m, *J* = 8.5, 2.2 Hz, 1H), 6.35 (s, 1H), 3.50 (t, *J* = 7.7 Hz, 2H), 2.70 (t, *J* = 7.8 Hz, 2H). ^13^C NMR (101 MHz, CD_3_OD) δ 161.44, 141.58, 135.71, 118.09, 117.96, 44.25, 26.73.

(**13, 81%**) Substrate: 84.0 mg, Product: 68.0 mg. ^1^H NMR (400.1 MHz, CDCl_3_) δ 8.29 (s, 1H), 7.48 (m, *J* = 8.0, 2.0 Hz, 1H), 7.41 (d, *J* = 1.9 Hz, 1H), 7.05 (d, *J* = 8.0 Hz, 1H), 3.78 (m, *J* = 11.1, 4.2 Hz, 2H), 2.74 – 2.66 (m, 2H). ^13^C NMR (101 MHz, CDCl_3_) δ 158.97, 143.47, 135.07, 133.90, 130.09, 129.15, 120.45, 47.20, 24.46.

(**14, 62%**) Substrate: 84.9 mg, Product: 52.6 mg. ^1^H NMR (400.1 MHz, CDCl_3_) δ 7.48 (d, *J* = 7.5 Hz, 1H), 7.37 – 7.26 (m, 2H), 7.18 (d, *J* = 7.7 Hz, 1H), 3.64 (t, *J* = 7.4 Hz, 2H), 2.70 (t, *J* = 7.3 Hz, 2H), 2.39 (s, 3H). ^13^C NMR (101 MHz, CDCl_3_) δ 164.74, 137.33, 130.79, 129.40, 127.44, 126.93, 125.44, 46.60, 25.96, 23.09.

(**15, 76%**) Substrate: 93.8 mg, Product: 71.3 mg. ^1^H NMR (400.1 MHz, CDCl_3_) δ 7.62 – 7.56 (m, 2H), 7.45 – 7.34 (m, 4H), 7.27 – 7.21 (m, 3H), 3.87 – 3.77 (t, 2H), 2.83 – 2.72 (t, 2H). ^13^C NMR (101 MHz, CDCl_3_) δ 167.39, 138.85, 130.77, 129.38, 128.84, 128.18, 128.01, 127.44, 126.61, 47.56, 26.33.

(**16, 92%**) Substrate: 51.8 mg, Product: 47.7 mg. ^1^H NMR (400.1 MHz, CDCl_3_) δ 8.06 (s, 1H), 7.65 (d, *J* = 7.8 Hz, 1H), 7.37 (m, *J* = 8.1, 0.8 Hz, 1H), 7.22 – 7.16 (m, 2H), 7.13 (m, *J* = 10.5, 4.0 Hz, 1H), 6.63 – 6.46 (m, 1H).^13^C NMR (101 MHz, CDCl_3_) δ 135.79, 127.87, 124.18, 122.02, 120.77, 119.85, 111.06, 102.64. [^5^](#_ENREF_5)

(**17, 89%**) Substrate: 56.0 mg, Product: 49.8 mg. ^1^H NMR (400.1 MHz, CD_3_OD) δ 7.41 (d, *J* = 7.7 Hz, 1H), 7.26 (d, *J* = 8.0 Hz, 1H), 7.05 – 6.91 (m, 3H), 2.38 (s, 3H). ^13^C NMR (101 MHz, CD_3_OD) δ 137.96, 128.93, 121.24, 121.06, 120.15, 119.86, 111.40, 111.21, 13.57.[^3^](#_ENREF_3)

(**18, 83%**) Substrate: 53.2 mg, Product: 44.2 mg. ^1^H NMR (400.1 MHz, CD_3_OD) δ 7.53 – 7.44 (m, 1H), 7.36 – 7.23 (m, 1H), 7.11 – 7.06 (m, 1H), 7.03 – 6.94 (m, 2H), 2.31 (d, *J* = 1.1 Hz, 3H). ^13^C NMR (101 MHz, CD_3_OD) δ 138.12, 129.58, 123.07, 122.10, 119.30, 119.19, 111.98, 111.24, 9.76.

(**19, 94%**) Substrate: 57.6 mg, Product: 54.1 mg. ^1^H NMR (400.1 MHz, CDCl_3_) δ 8.22 (s, 1H), 7.24 (d, *J* = 8.2 Hz, 1H), 7.21 – 7.17 (m, 1H), 7.14 – 7.09 (m, 1H), 6.93 (d, *J* = 7.1 Hz, 1H), 6.58 (s, 1H), 2.58 (s, 3H). ^13^C NMR (101 MHz, CDCl_3_) δ 135.47, 130.23, 127.78, 123.55, 122.08, 119.90, 108.66, 101.06, 18.86.

(**20, 63%**) Substrate: 70.0 mg, Product: 44.1 mg. ^1^H NMR (400.1 MHz, CD_3_OD) δ 7.54 – 7.42 (m, 1H), 7.32 (dt, *J* = 8.1, 0.8 Hz, 1H), 7.11 – 7.06 (m, 1H), 7.03 – 6.95 (m, 2H), 2.31 (d, *J* = 1.1 Hz, 3H). ^13^C NMR (101 MHz, CD_3_OD) δ 155.14, 132.95, 129.82, 126.16, 112.73, 112.56, 103.08, 102.19, 56.26.

(**21, 88%**) Substrate: 99.7 mg, Product: 87.7 mg. ^1^H NMR (400.1 MHz, CDCl_3_) δ 8.36 (s, 1H), 7.32 (m, *J* = 10.5, 7.9 Hz, 2H), 7.25 – 7.21 (m, 1H), 7.09 – 7.02 (m, 1H), 6.61 (t, *J* = 2.3 Hz, 1H). ^13^C NMR (101 MHz, CDCl_3_) δ 136.03, 128.68, 124.78, 122.92, 122.76, 114.74, 110.31, 103.00.[^6^](#_ENREF_6)

(**22, 68%**) Substrate: 76.3 mg, Product: 51.9 mg. ^1^H NMR (400.1 MHz, CDCl_3_) δ 8.99 (s, 1H), 8.61 (d, *J* = 2.1 Hz, 1H), 8.10 (m, *J* = 9.0, 2.2 Hz, 1H), 7.46 (d, *J* = 9.0 Hz, 1H), 7.42 – 7.35 (m, 1H), 6.73 (d, *J* = 2.2 Hz, 1H). ^13^C NMR (101 MHz, CDCl_3_) δ 141.82, 138.93, 129.16, 127.26, 117.98, 117.58, 111.19, 104.92.[^6^](#_ENREF_6)

(**23, 73%**) Substrate: 66.4 mg, Product: 48.5 mg. ^1^H NMR (400.1 MHz, CDCl_3_) δ 8.86 (s, 2H), 8.12 (m, *J* = 6.4, 3.5 Hz, 2H), 7.79 (m, *J* = 6.4, 3.4 Hz, 2H). ^13^C NMR (101 MHz, CDCl_3_) δ 144.99, 143.04, 130.13, 129.52.[^1^](#_ENREF_1)

**IV. NMR spectra of the products**
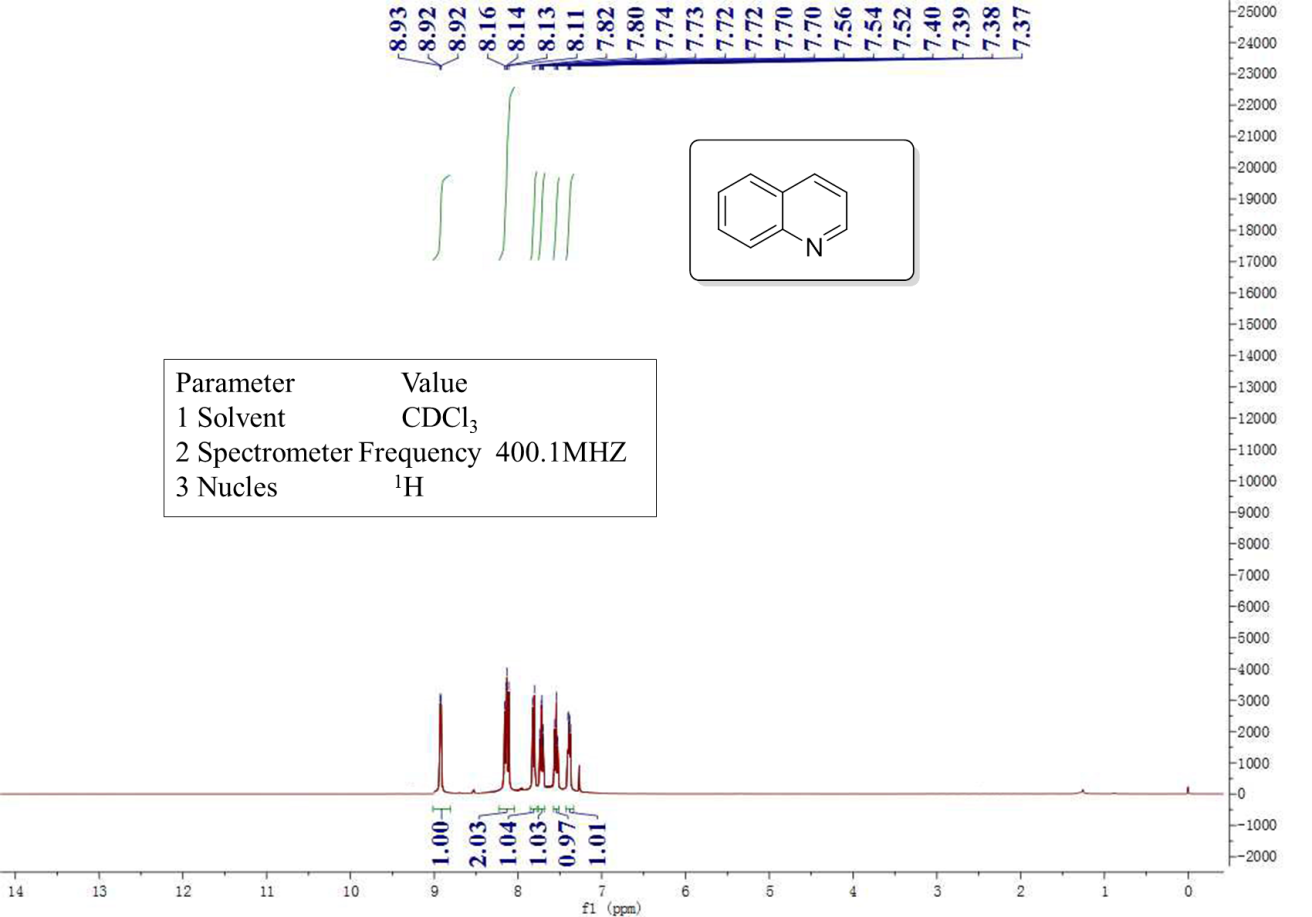


**Supplementary Figure 10 ^1^H NMR spectra of the quinoline**
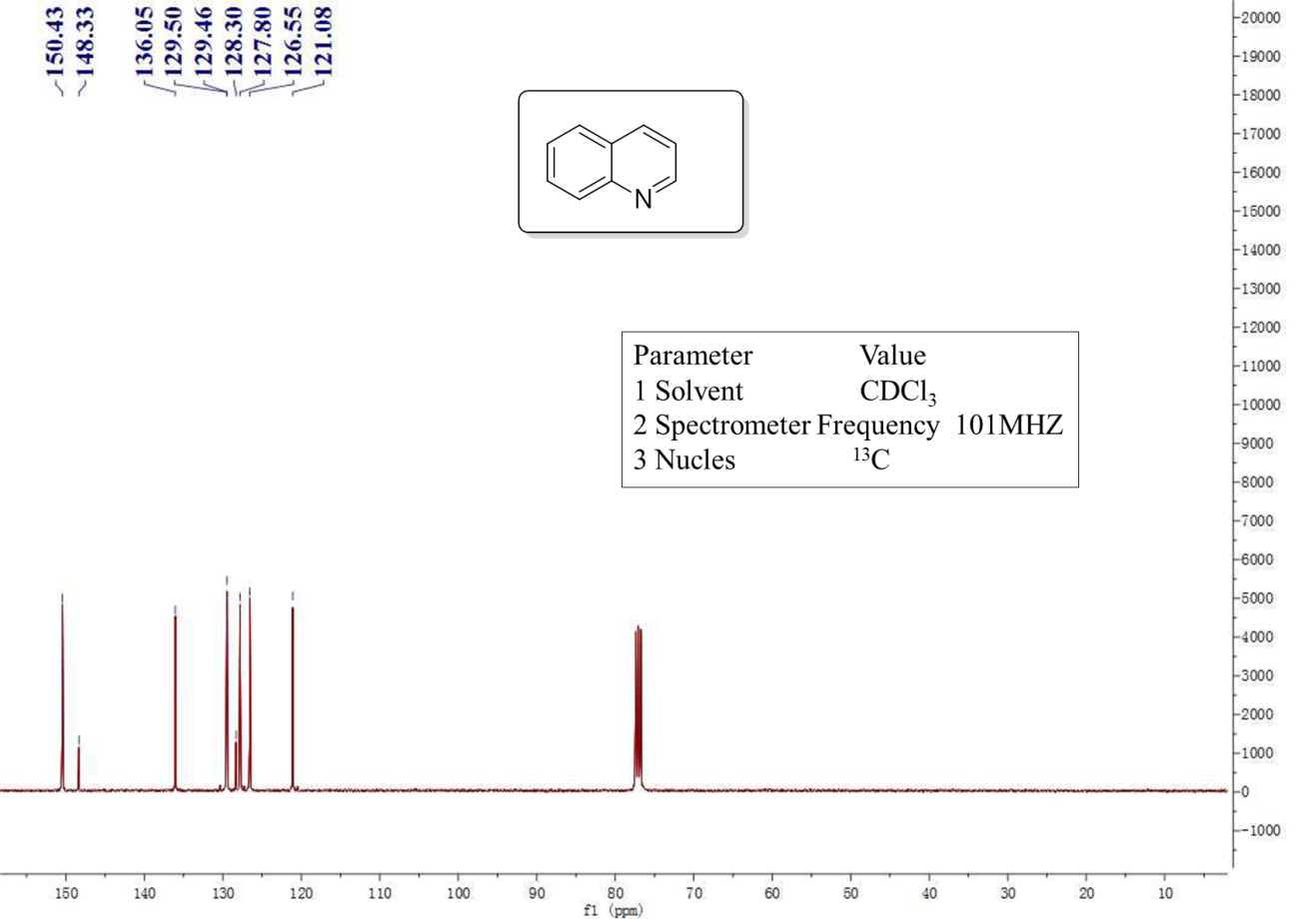


**Supplementary Figure 11 ^13^C NMR spectra of the quinoline**


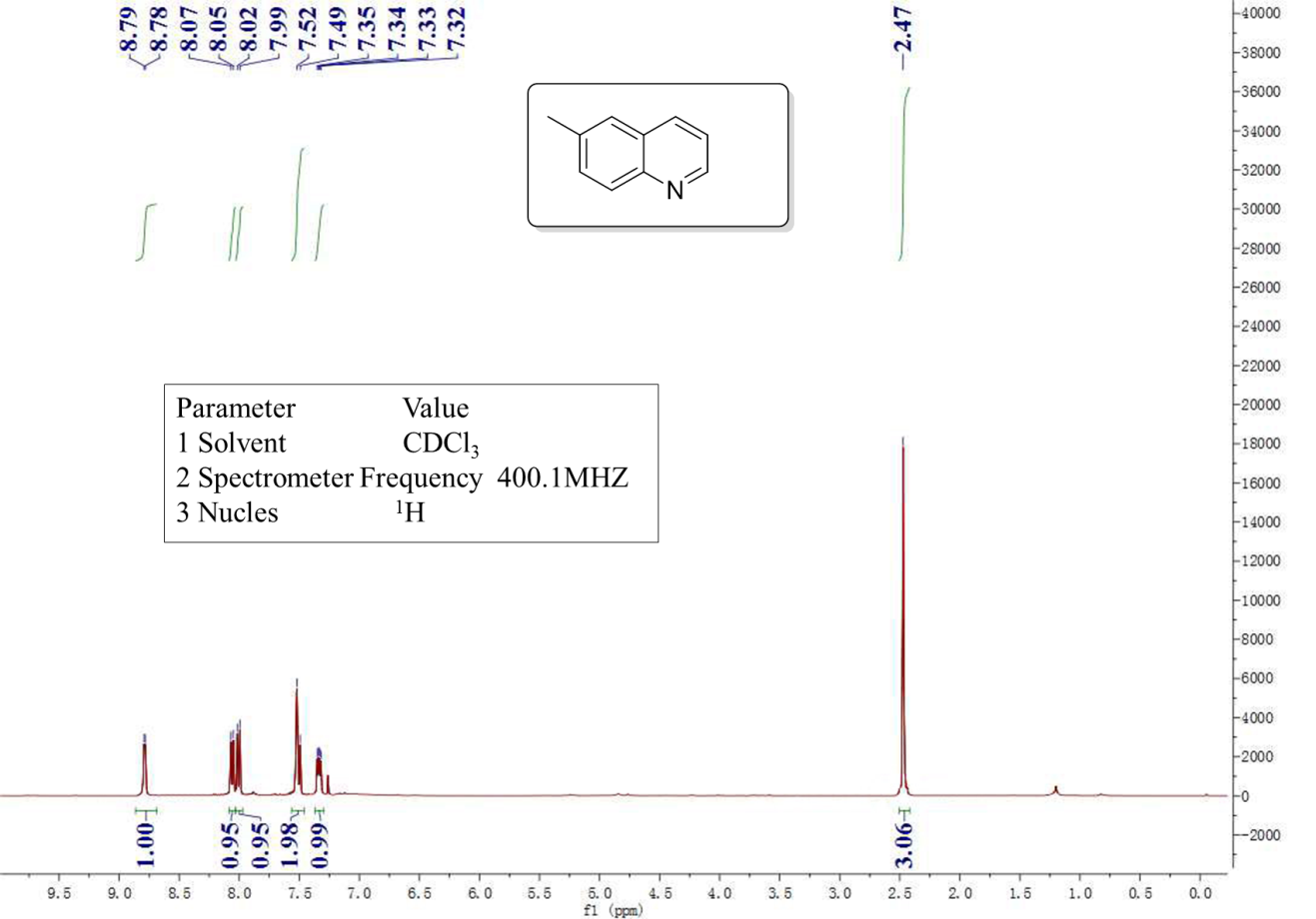


**Supplementary Figure 12 ^1^H NMR spectra of the 6-methylquinoline**


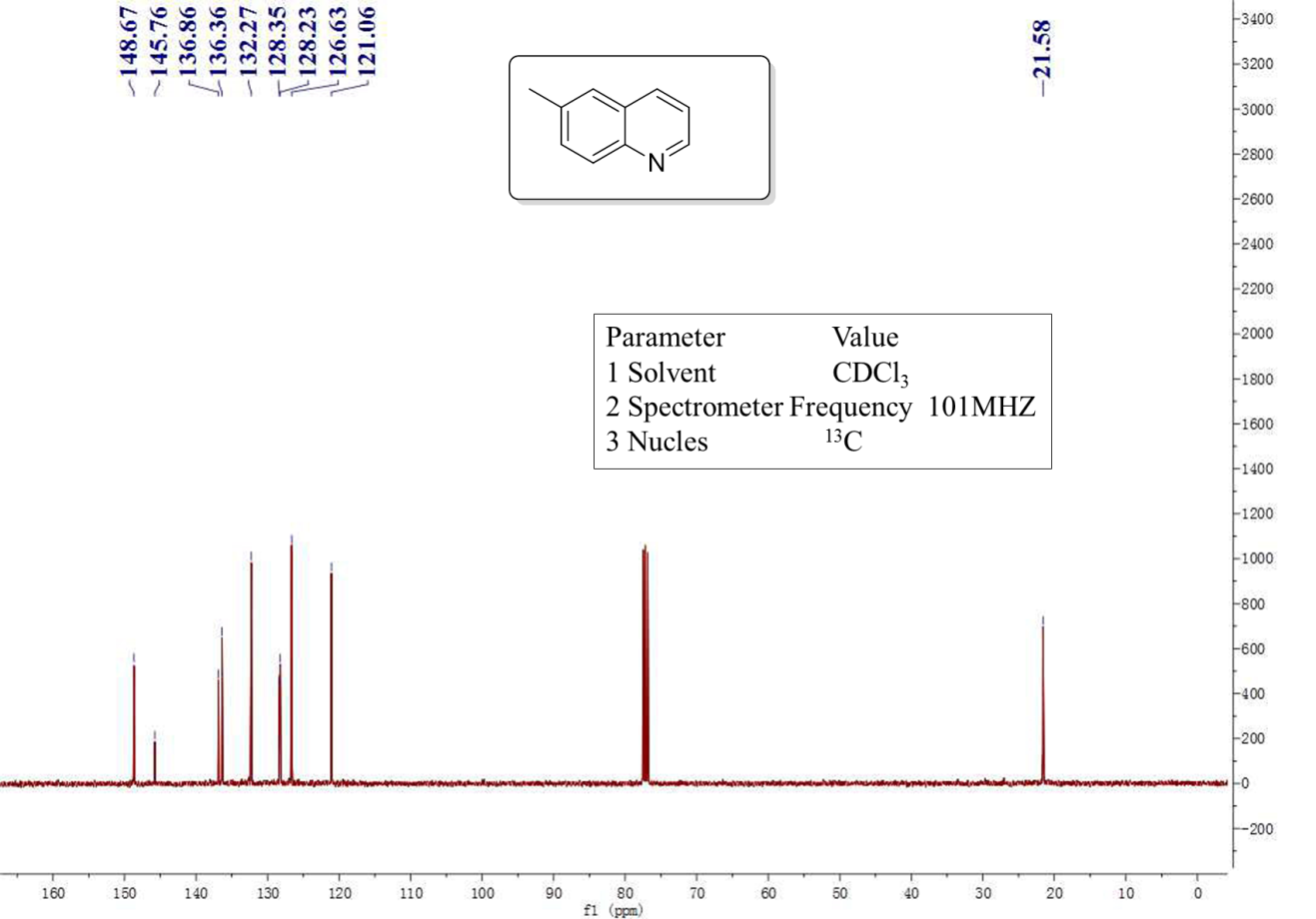


**Supplementary Figure 13 ^13^C NMR spectra of the 6-methylquinoline**


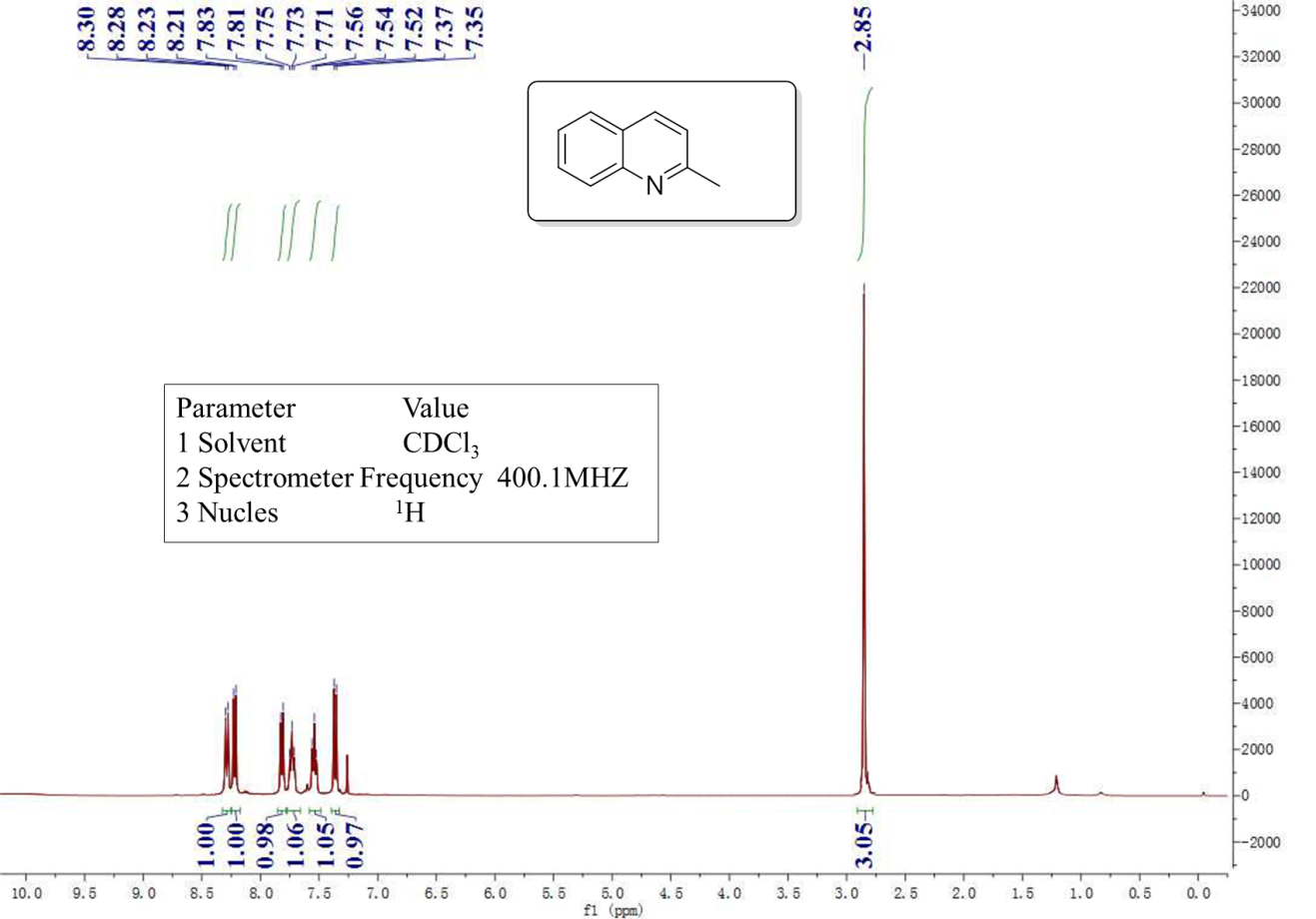


**Supplementary Figure 14 ^1^H NMR spectra of the 2-methylquinoline**


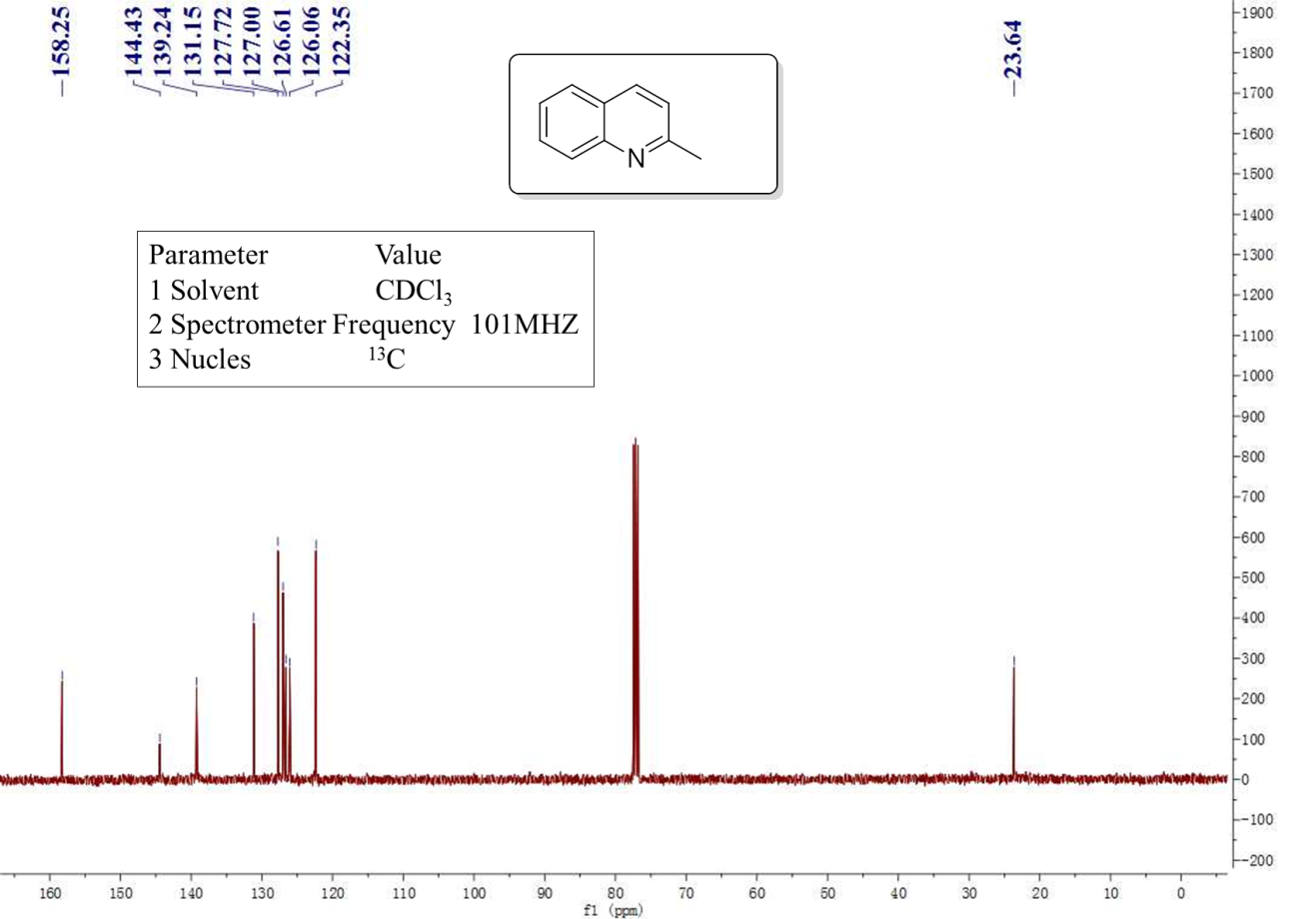


**Supplementary Figure 15 ^13^C NMR spectra of the 2-methylquinoline**


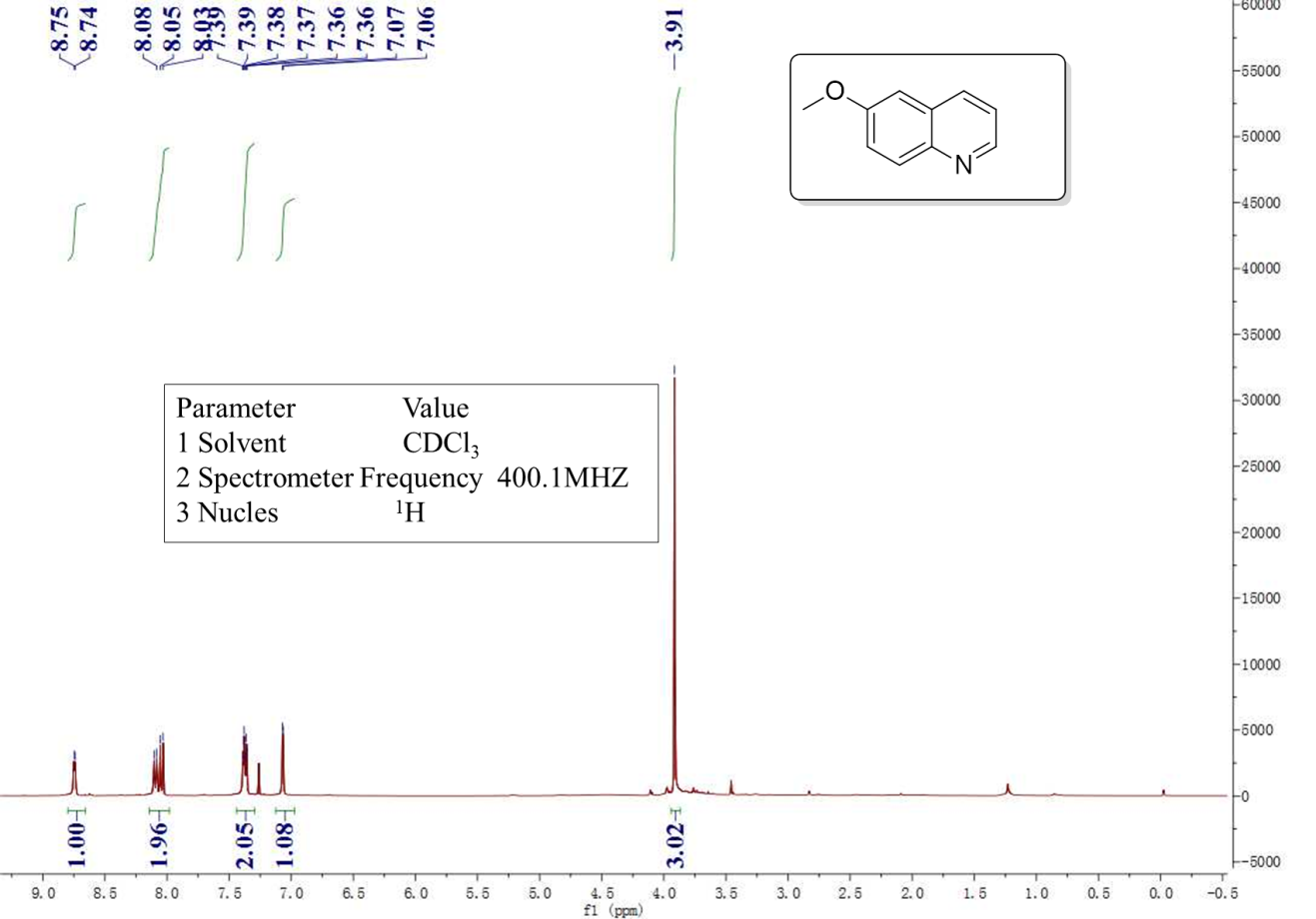


**Supplementary Figure 16 ^1^H NMR spectra of the 6-methoxyquinoline**


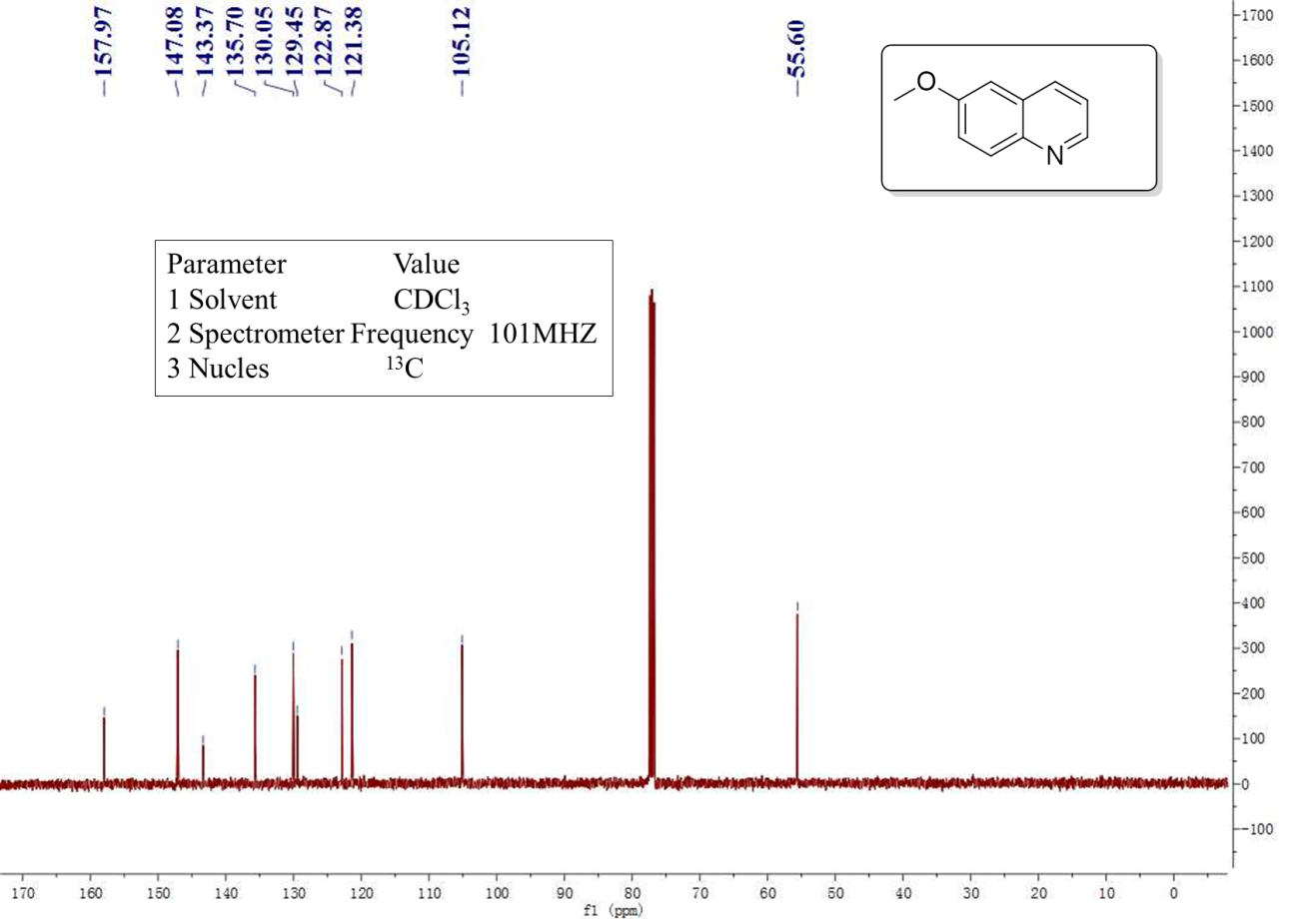


**Supplementary Figure 17 ^13^C NMR spectra of the 6-methoxyquinoline**


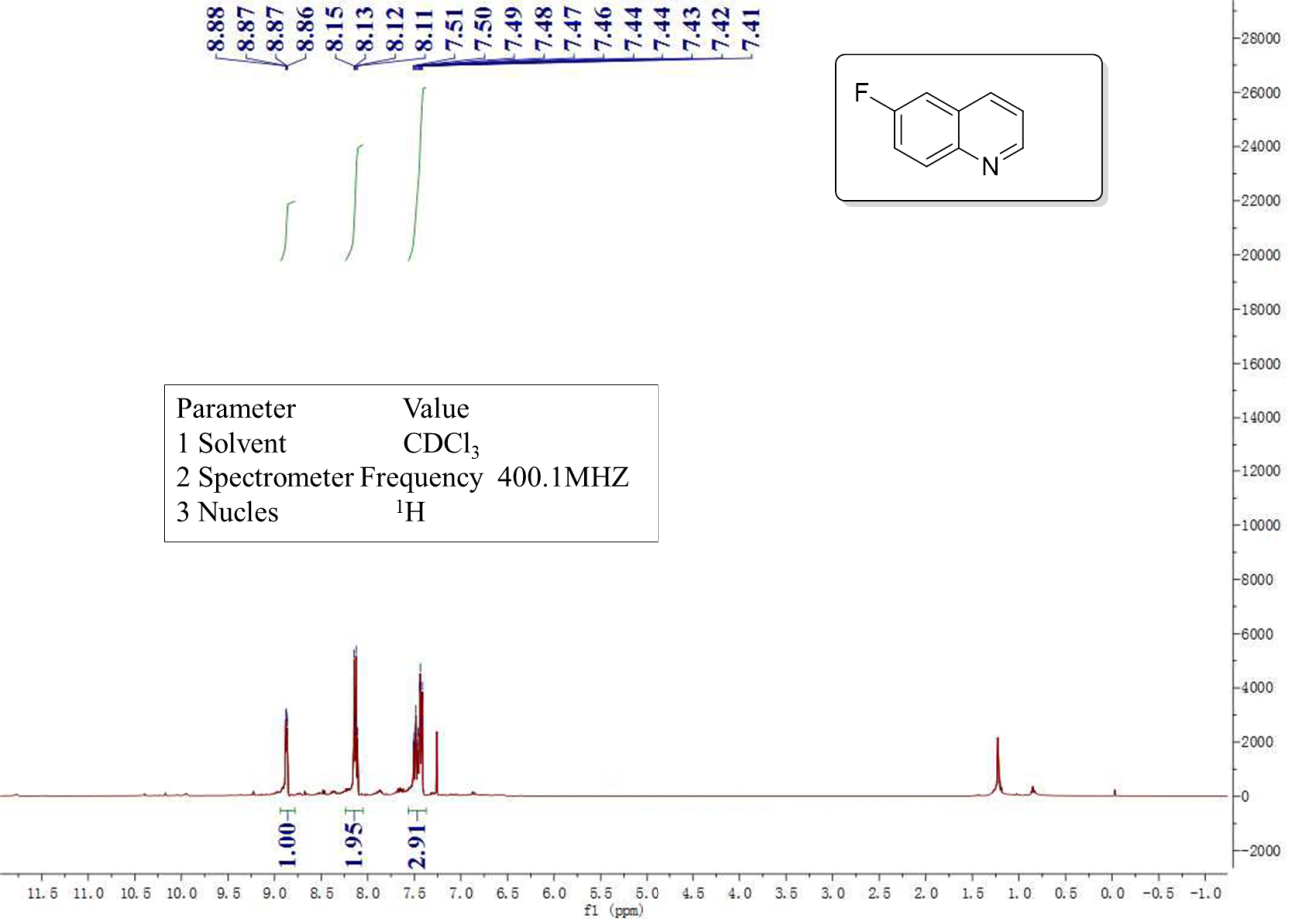


**Supplementary Figure 18 ^1^H NMR spectra of the 6-fluoroquinoline**


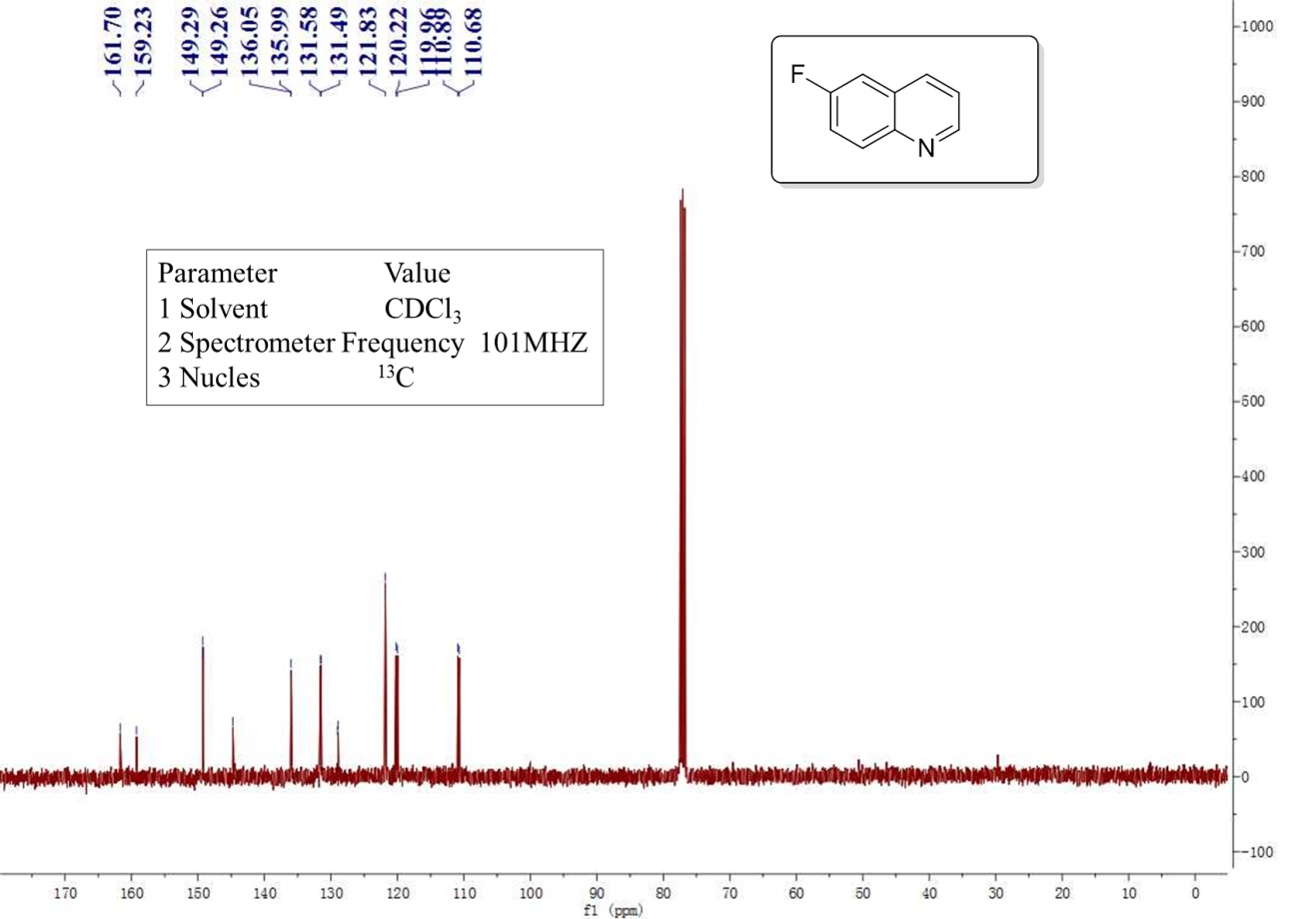


**Supplementary Figure 19 ^13^C NMR spectra of the 6-fluoroquinoline**


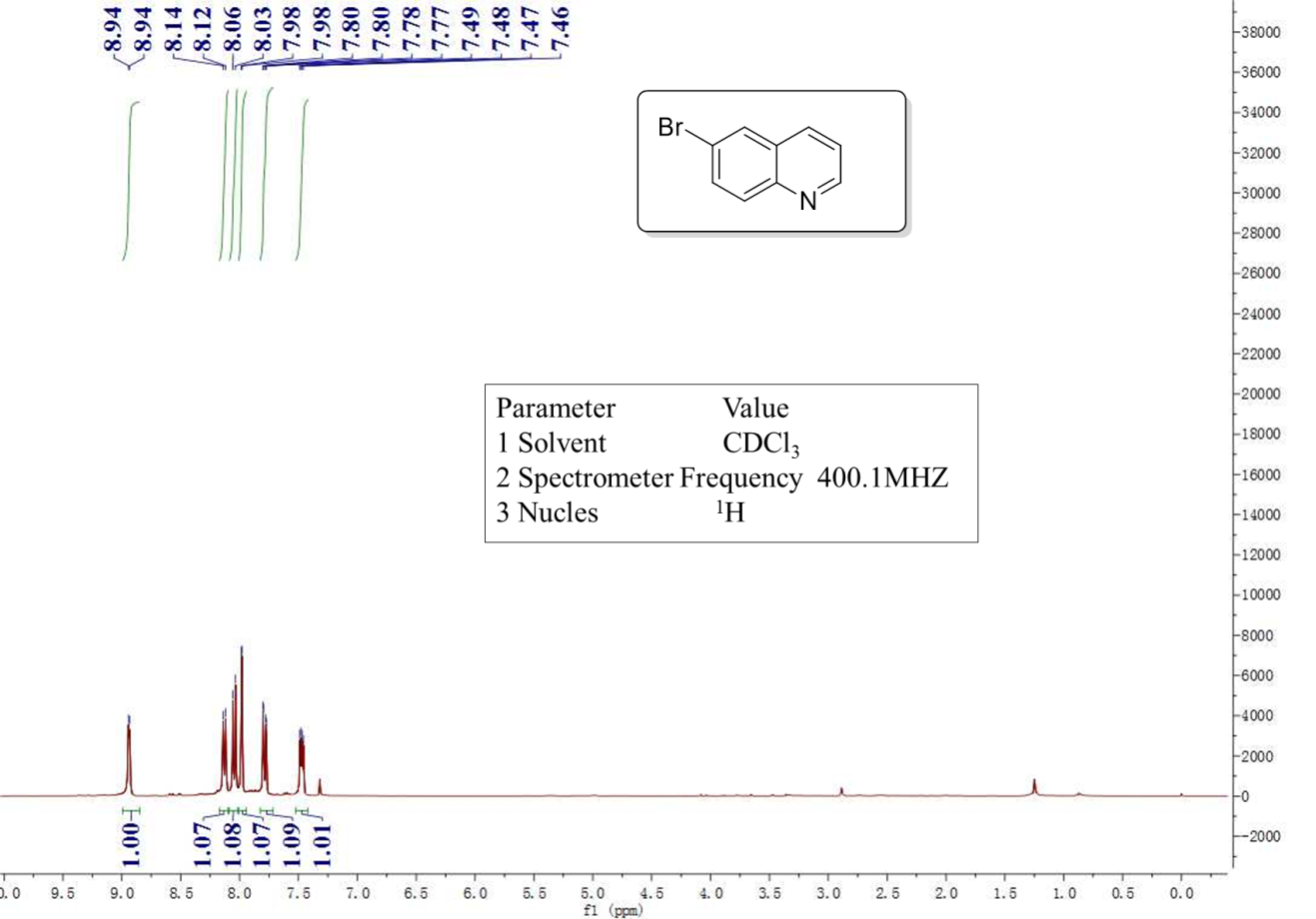


**Supplementary Figure 20 ^1^H NMR spectra of the 6-bromoquinoline**


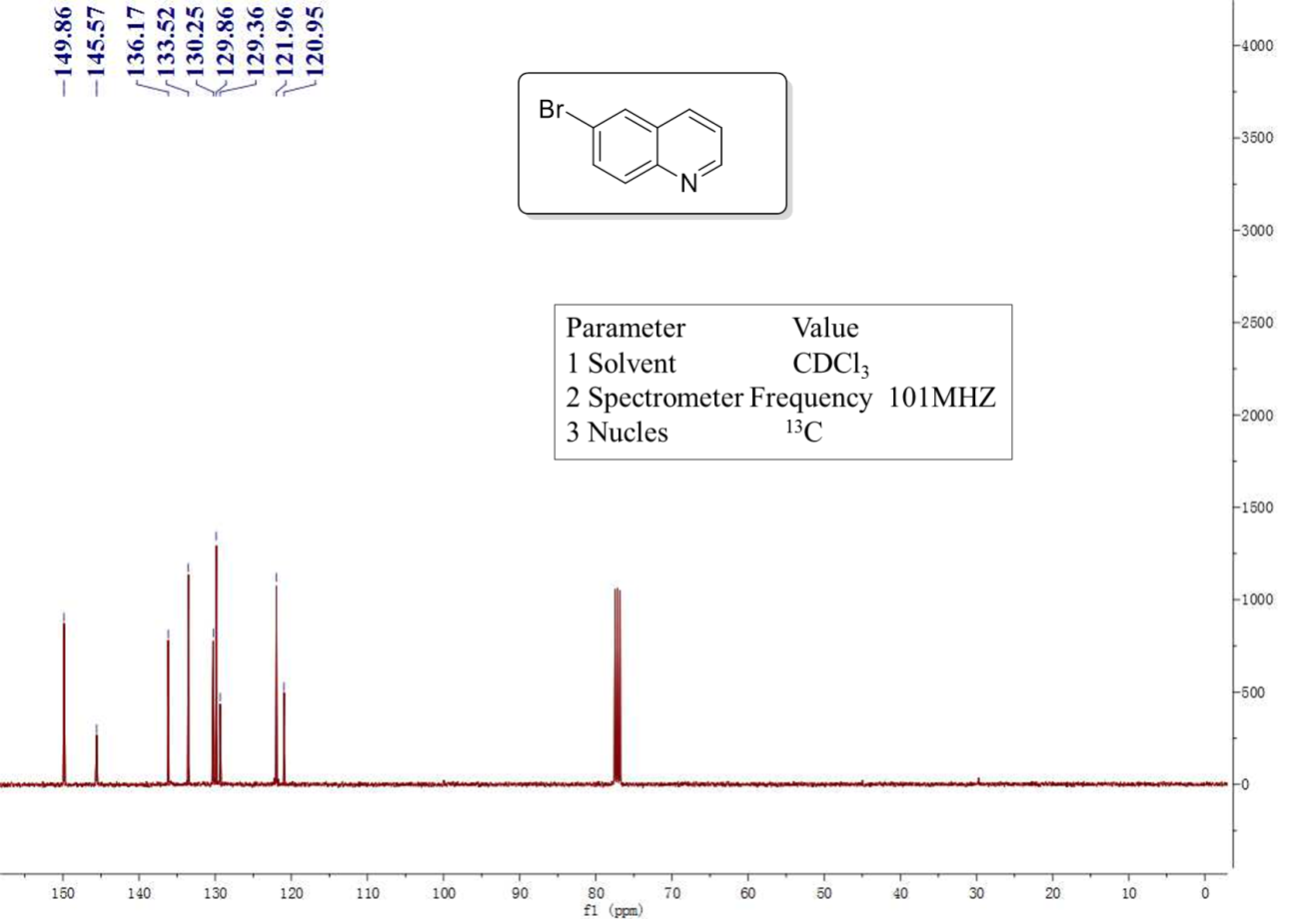


**Supplementary Figure 21^13^C NMR spectra of the 6-bromoquinoline**


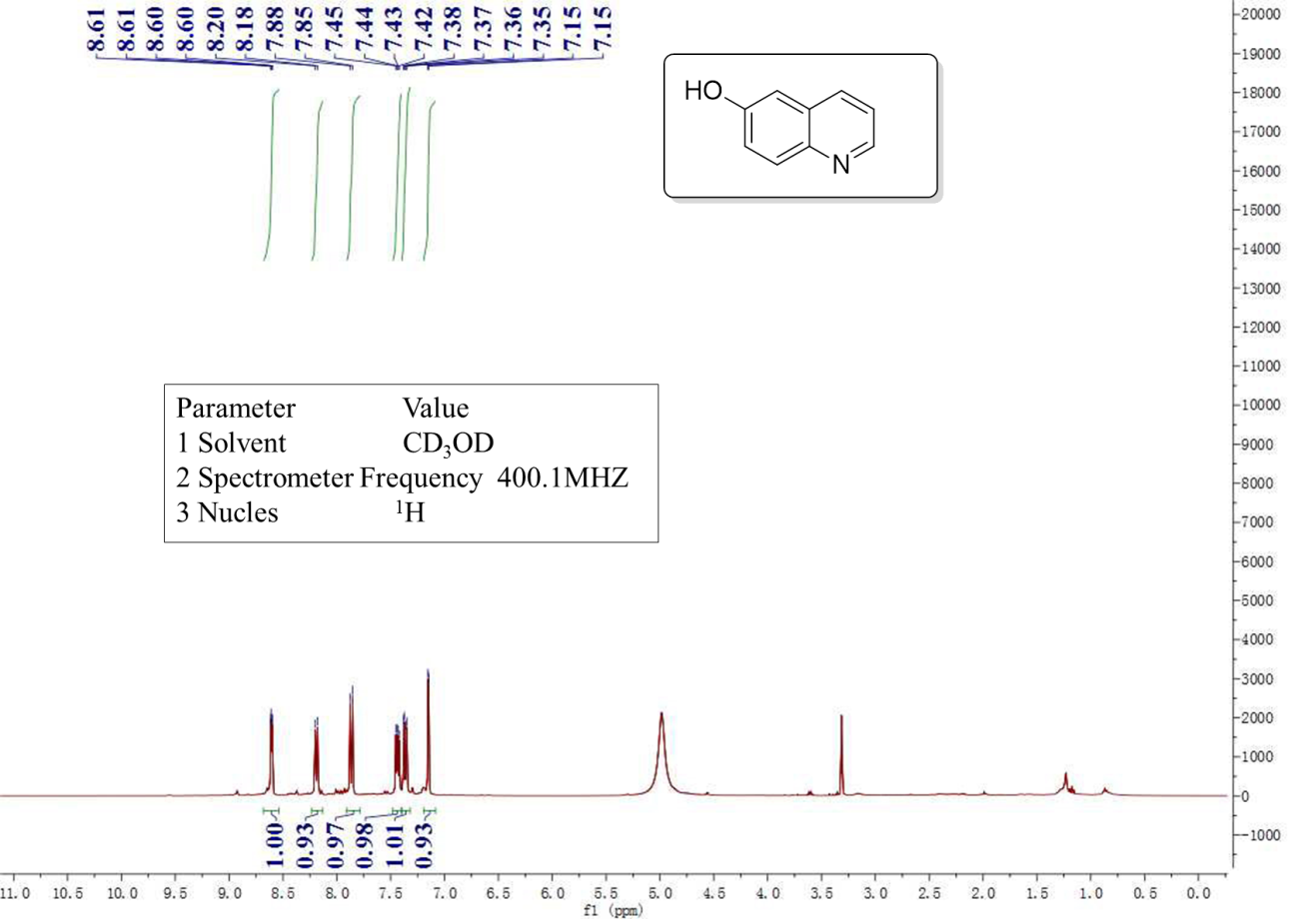


**Supplementary Figure 22 ^1^H NMR spectra of the quinolin-7-ol**


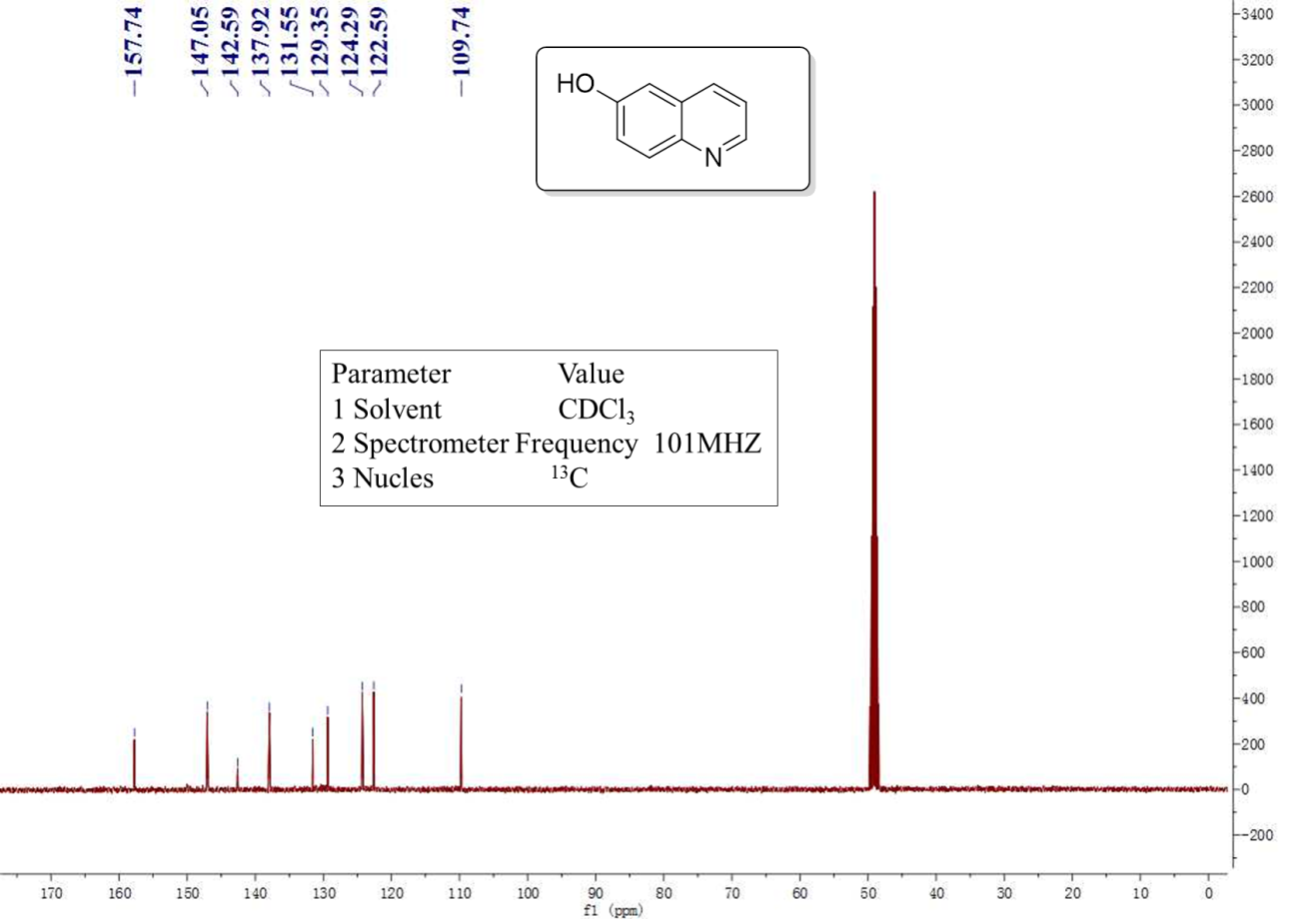


**Supplementary Figure 23 ^13^C NMR spectra of the quinolin-7-ol**


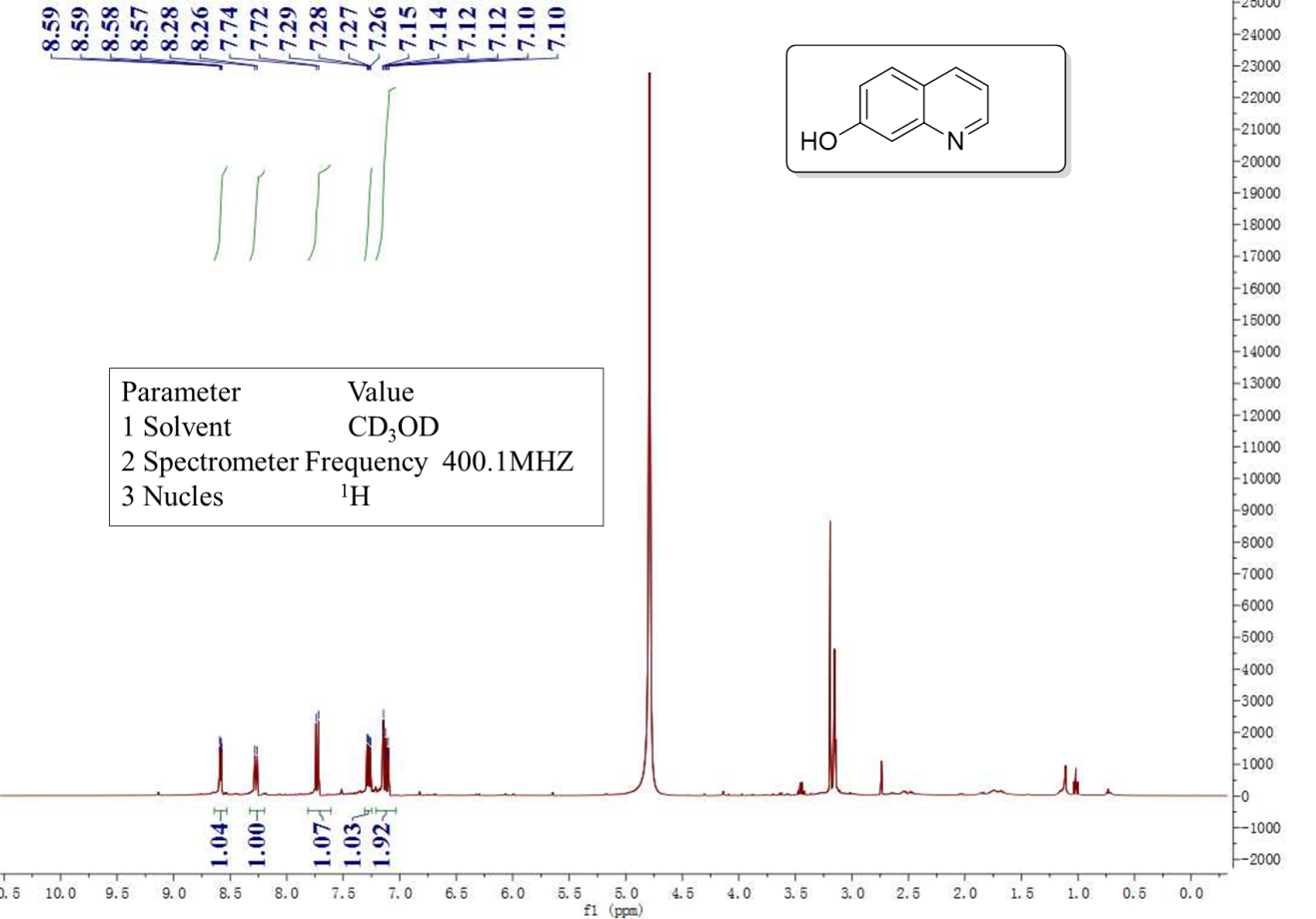


**Supplementary Figure 24 ^1^H NMR spectra of the quinolin-7-ol**


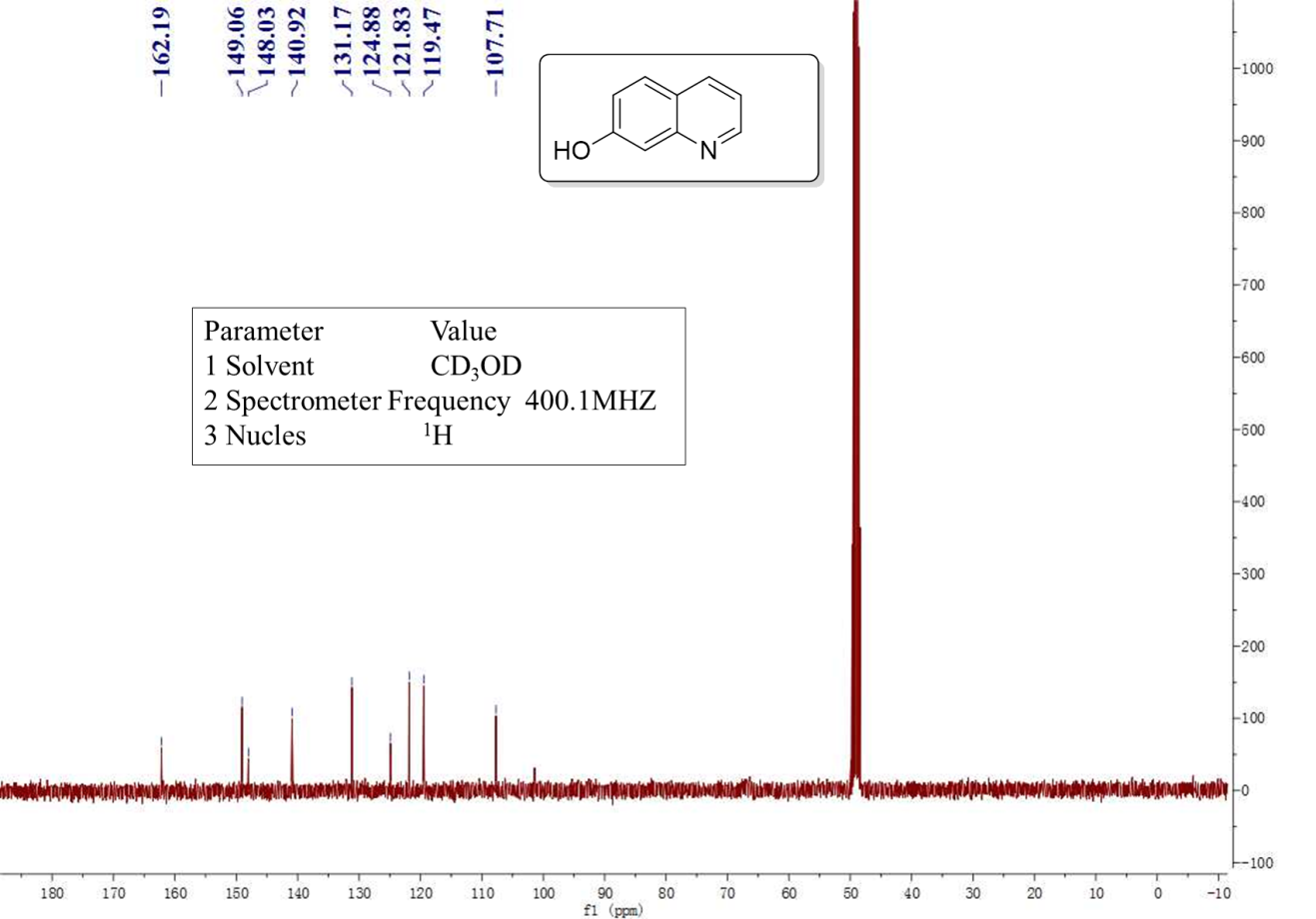


**Supplementary Figure 25 ^13^C NMR spectra of the quinolin-8-ol**


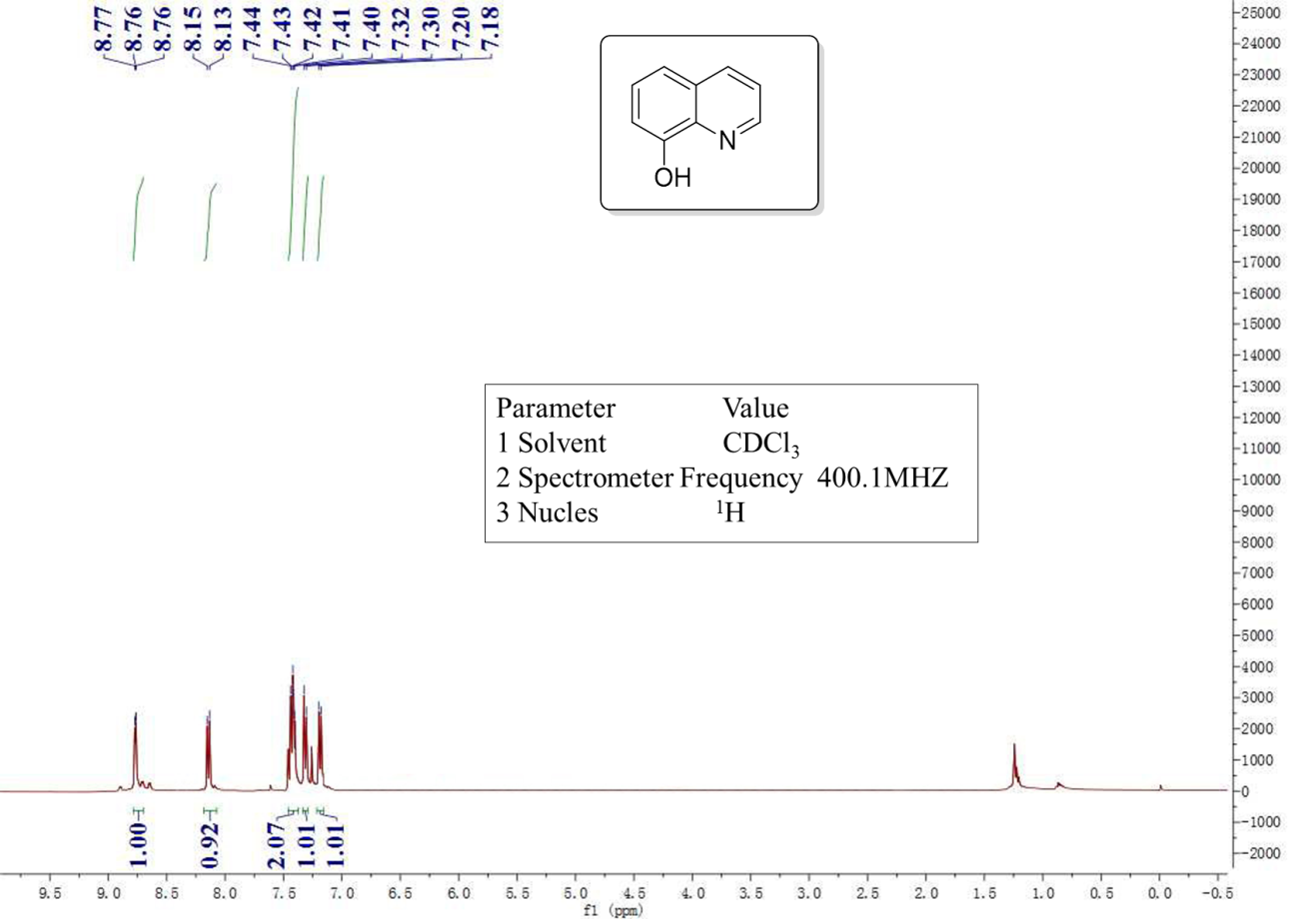


**Supplementary Figure 26 ^1^H NMR spectra of the quinolin-8-ol**


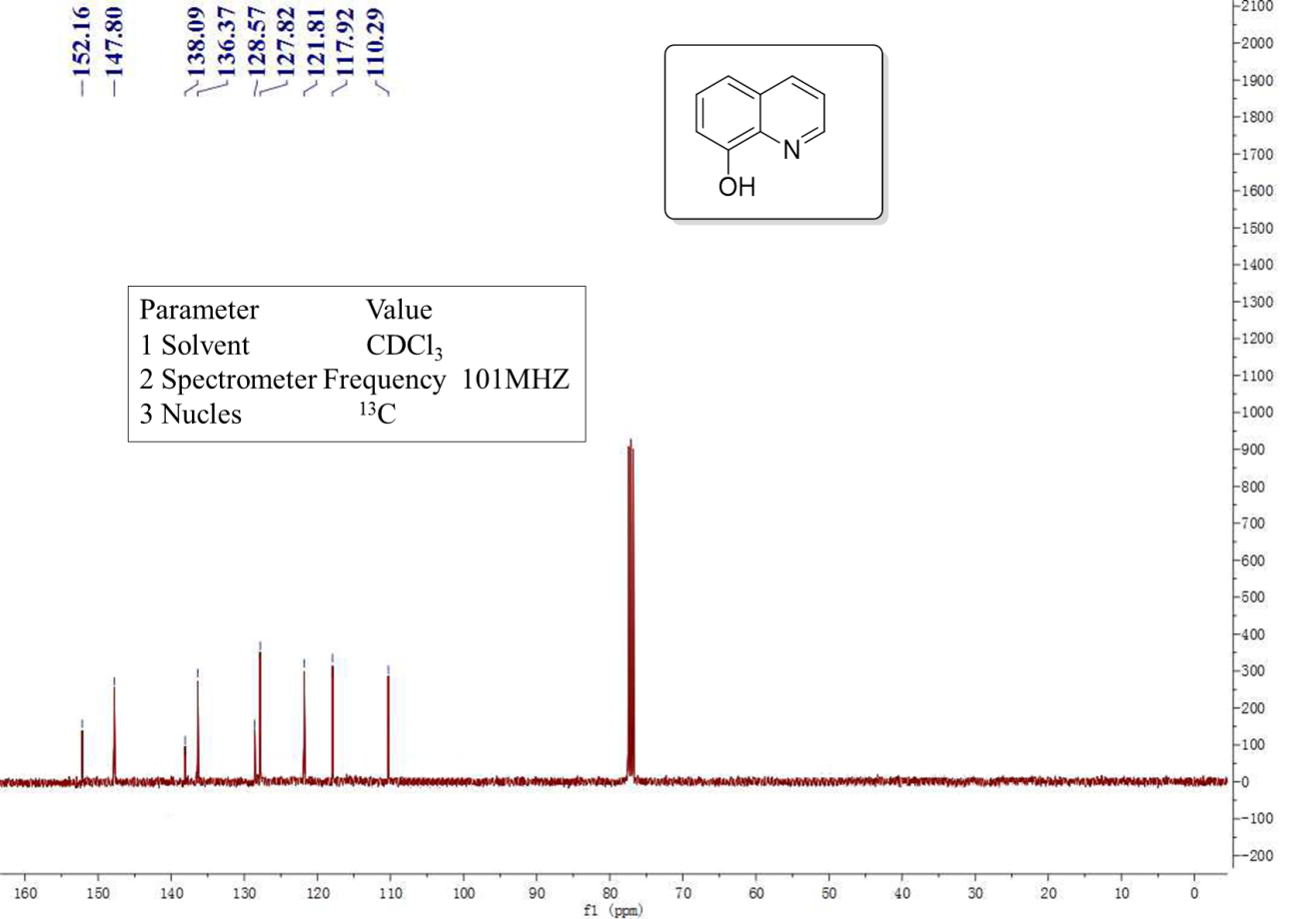


**Supplementary Figure 27 ^13^C NMR spectra of the 6-fluoro-2-methylquinoline**


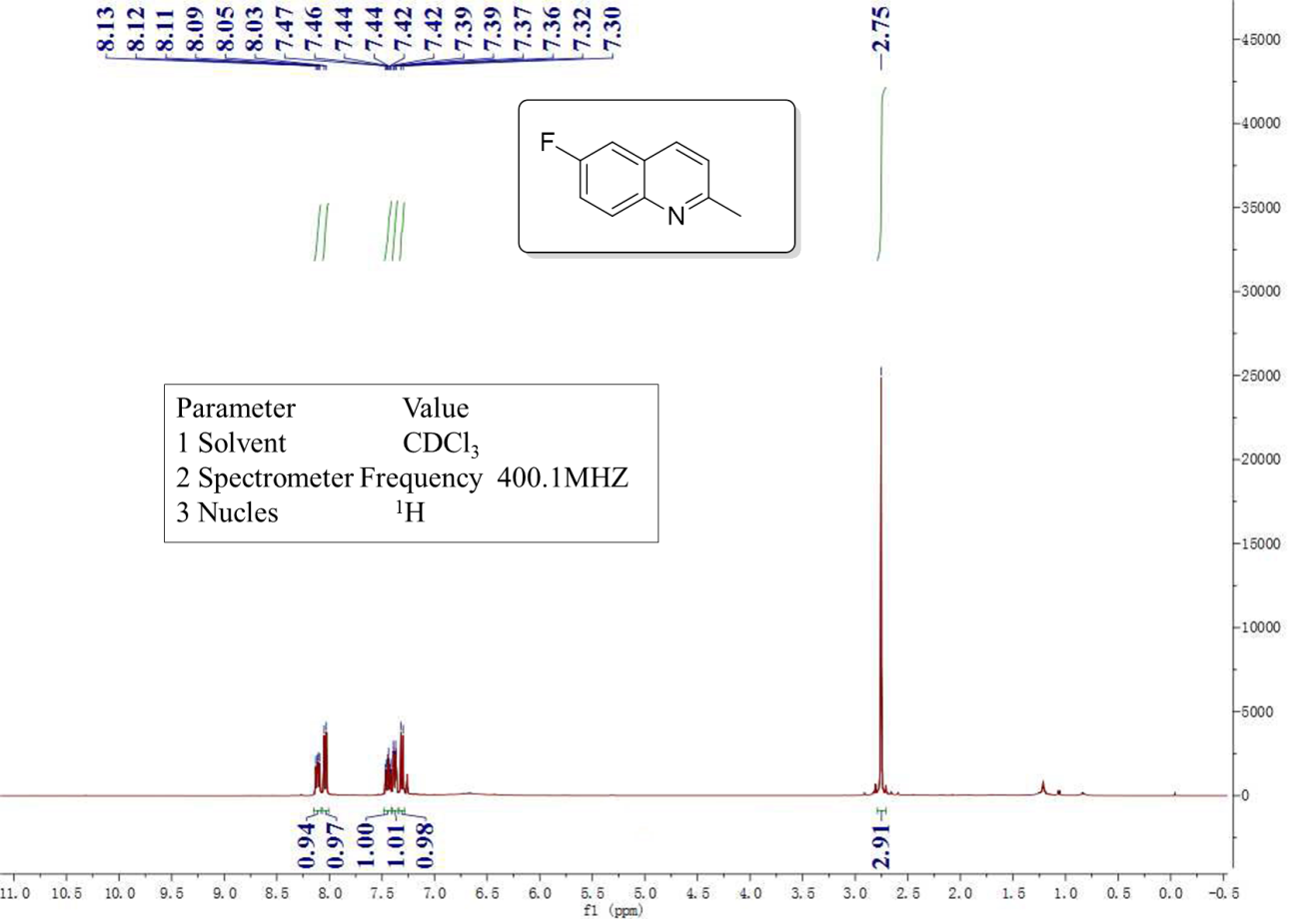


**Supplementary Figure 28 ^1^H NMR spectra of the 6-fluoro-2-methylquinoline**


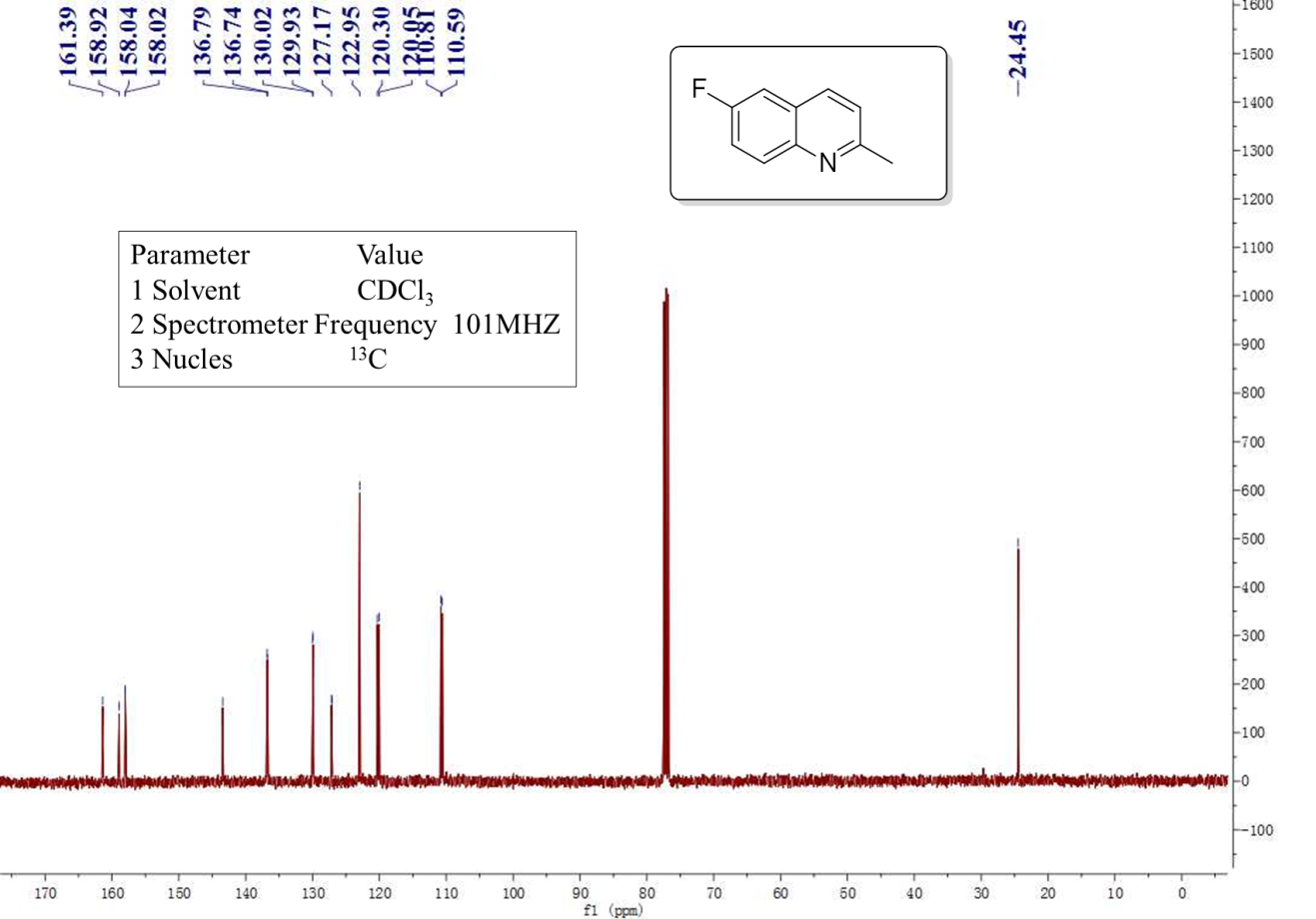


**Supplementary Figure 29 ^13^C NMR spectra of the 6-fluoro-2-methylquinoline**


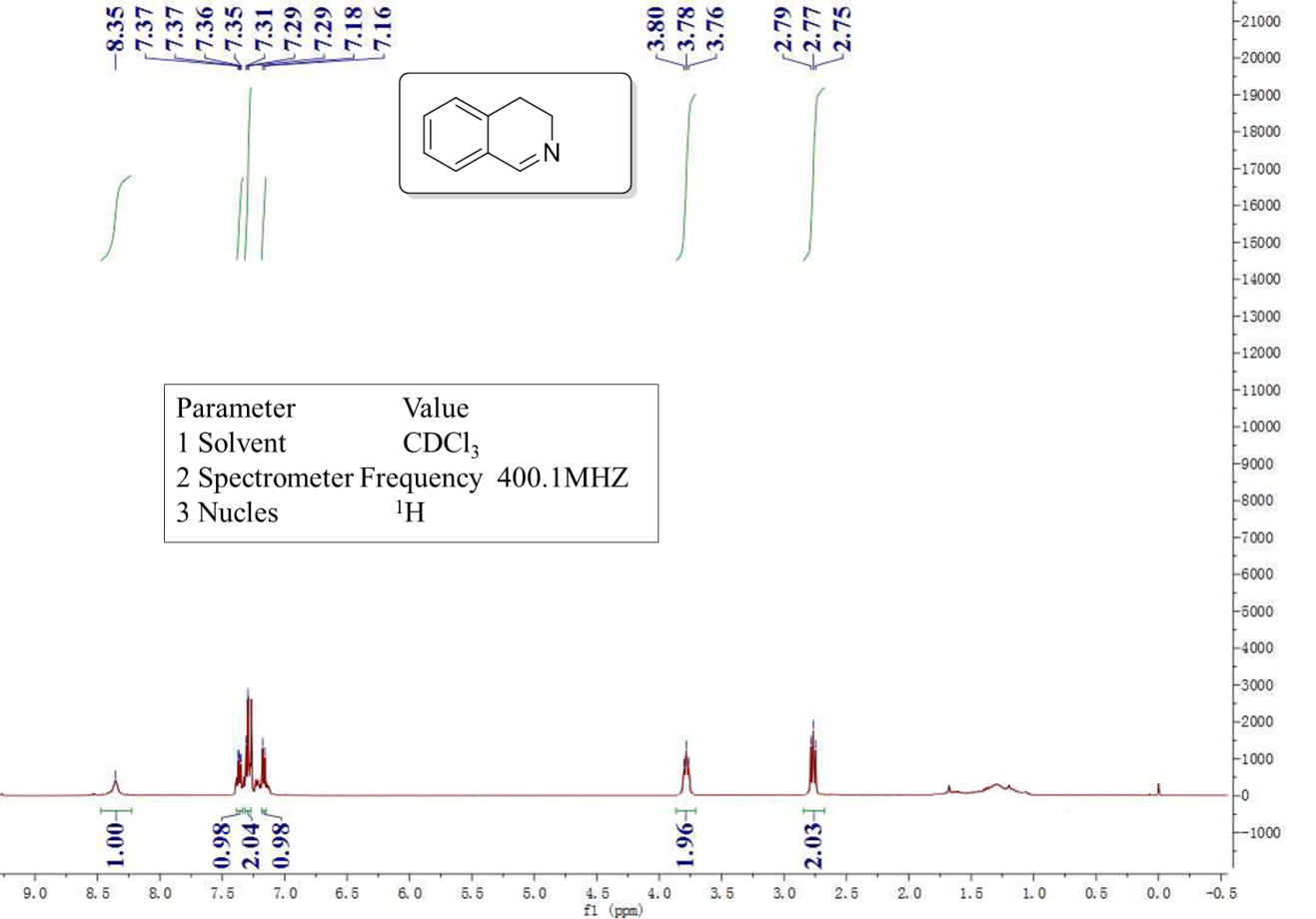


**Supplementary Figure 30 ^1^H NMR spectra of the 3,4-dihydroisoquinoline**


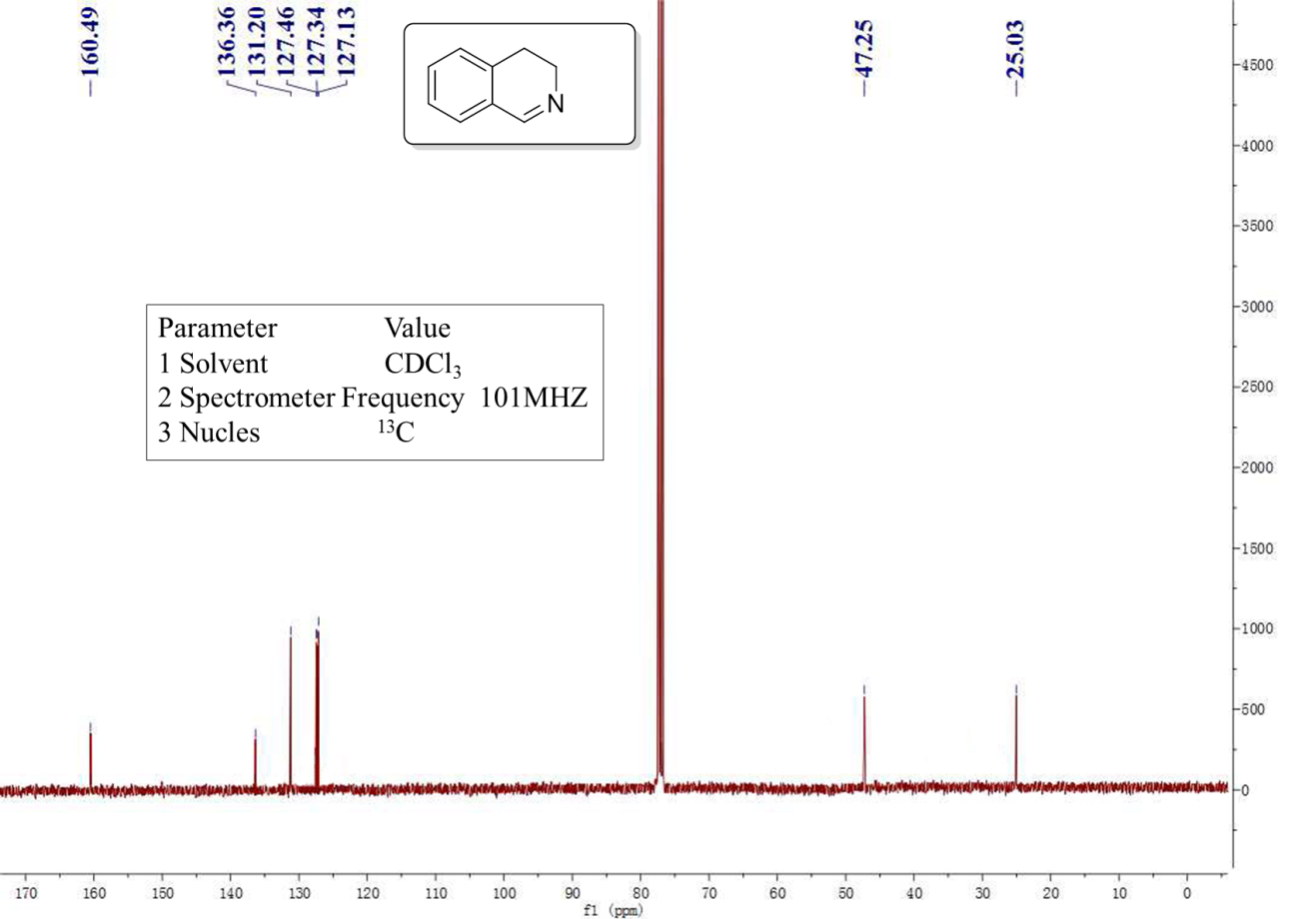


**Supplementary Figure 31 ^13^C NMR spectra of the 3,4-dihydroisoquinoline**


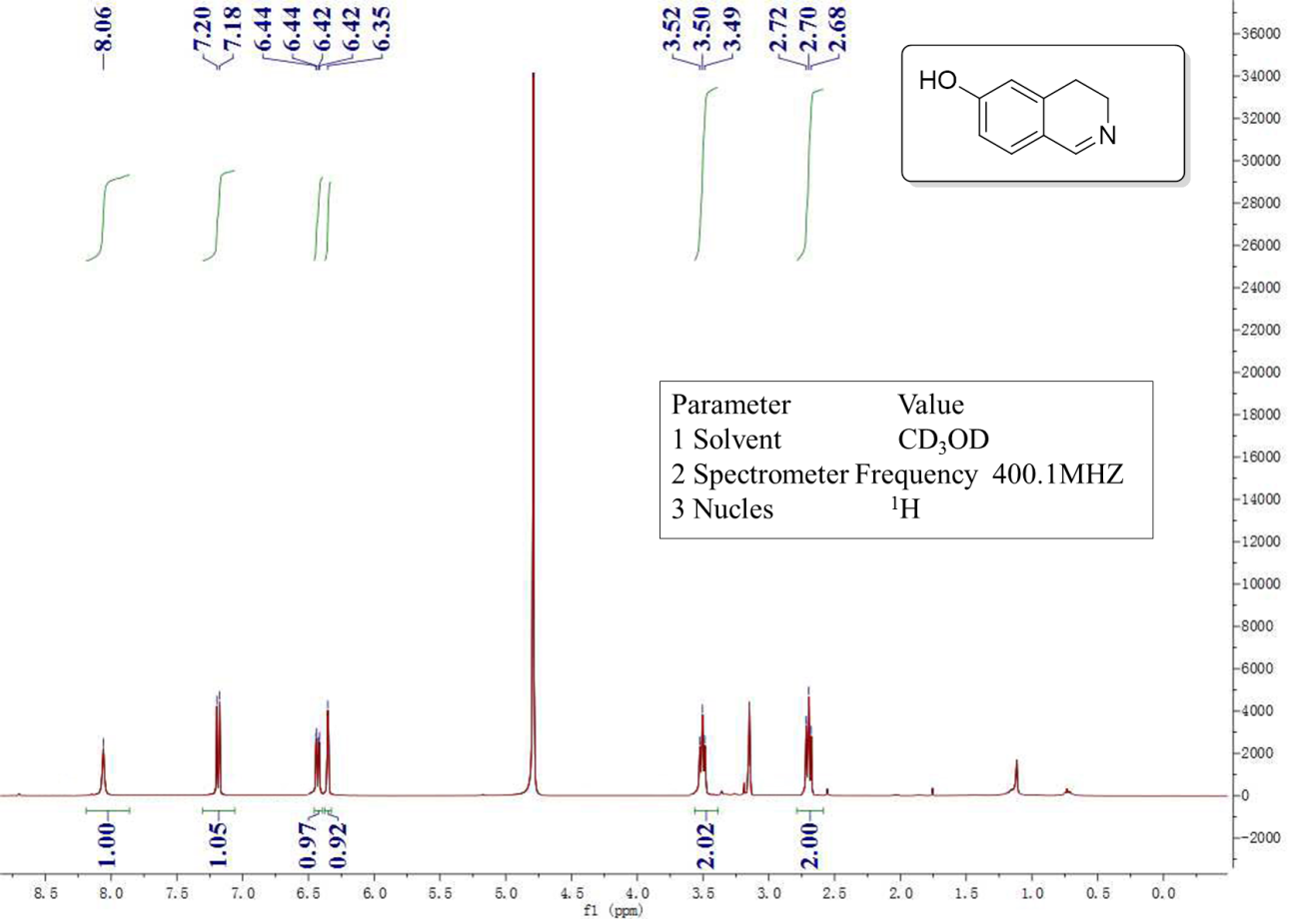


**Supplementary Figure 32 ^1^H NMR spectra of the 3,4-dihydroisoquinolin-6-ol**


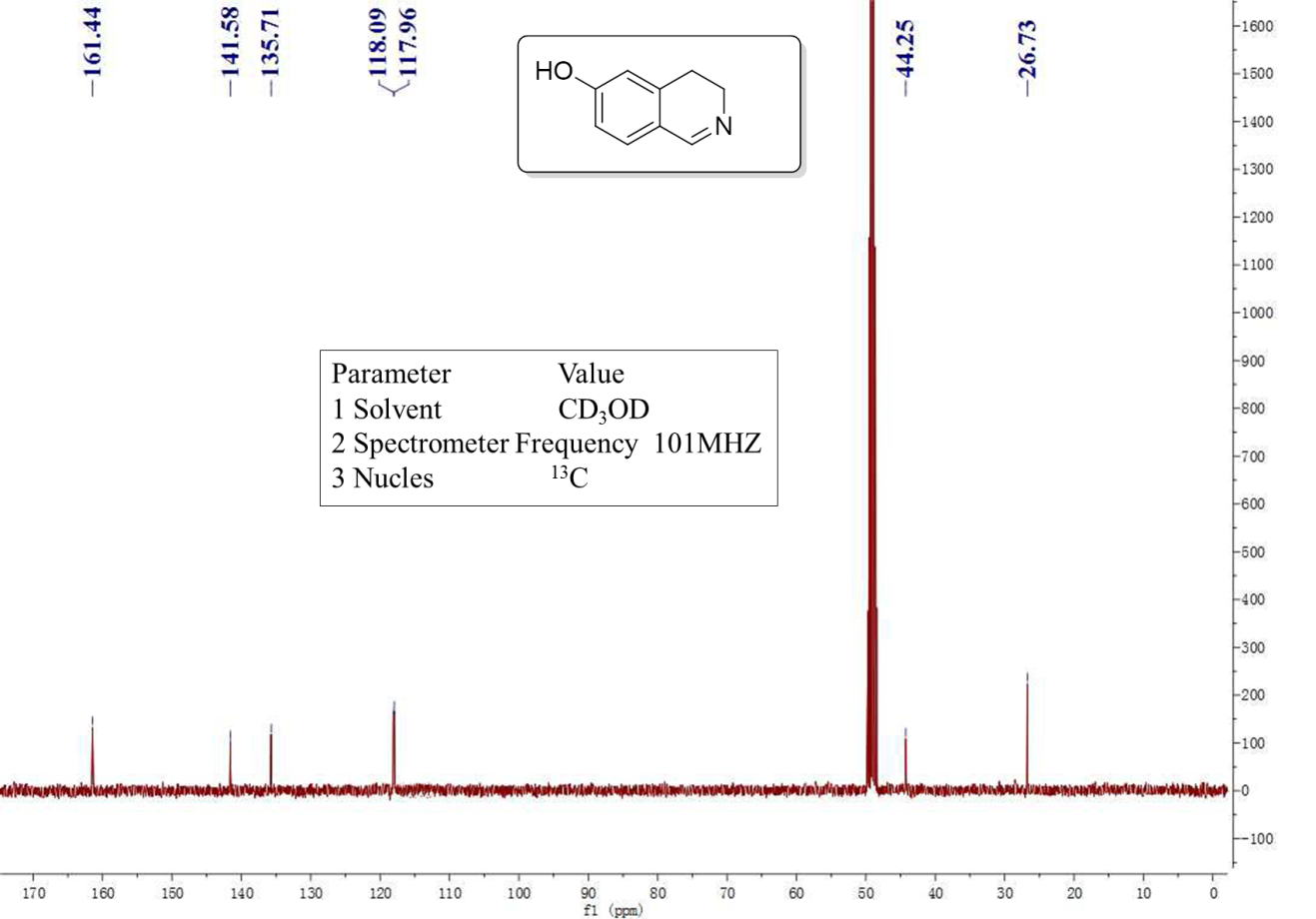


**Supplementary Figure 33 ^13^C NMR spectra of the 3,4-dihydroisoquinolin-6-ol**


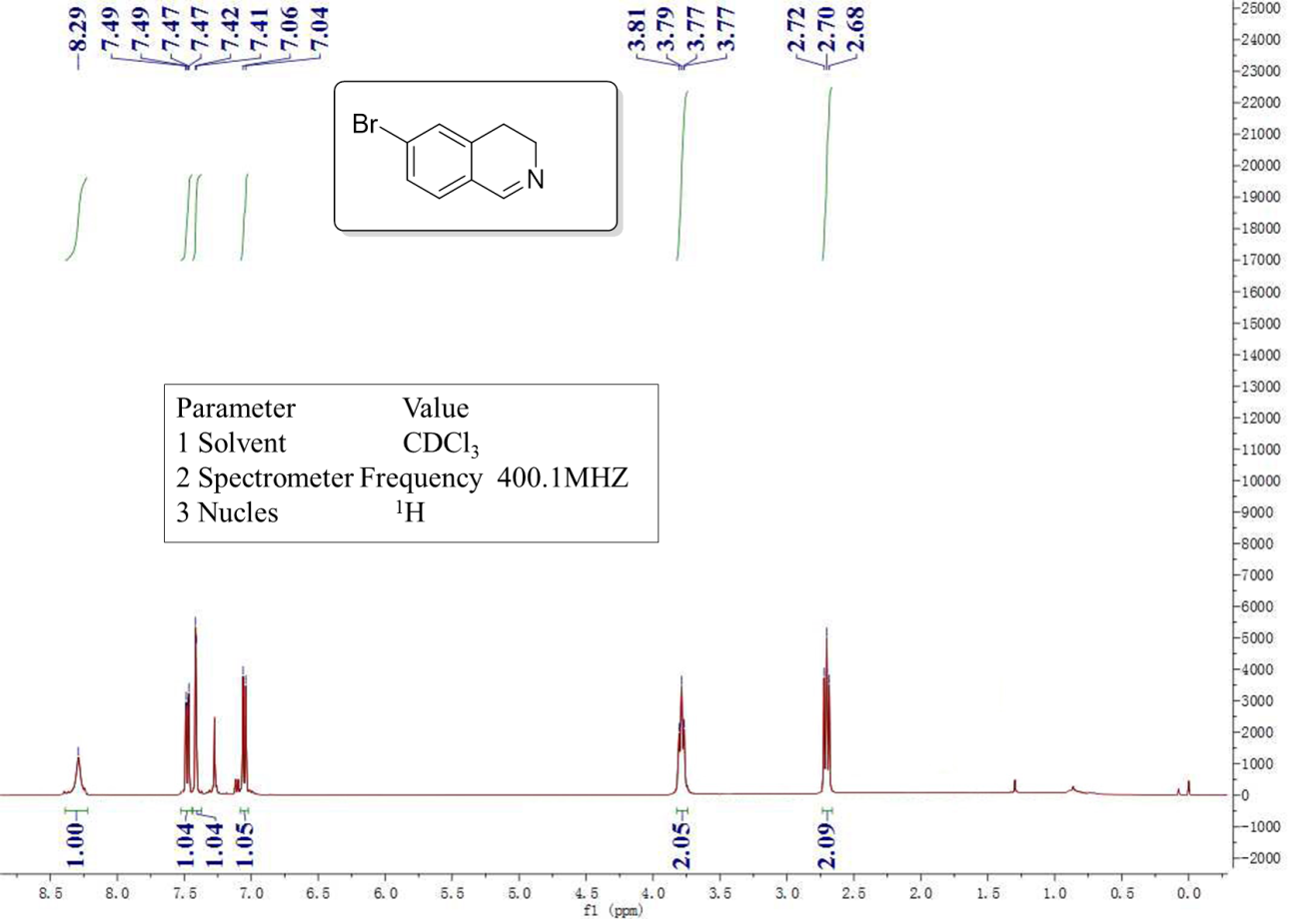


**Supplementary Figure 34 ^1^H NMR spectra of the 6-bromo-3,4-dihydroisoquinoline**


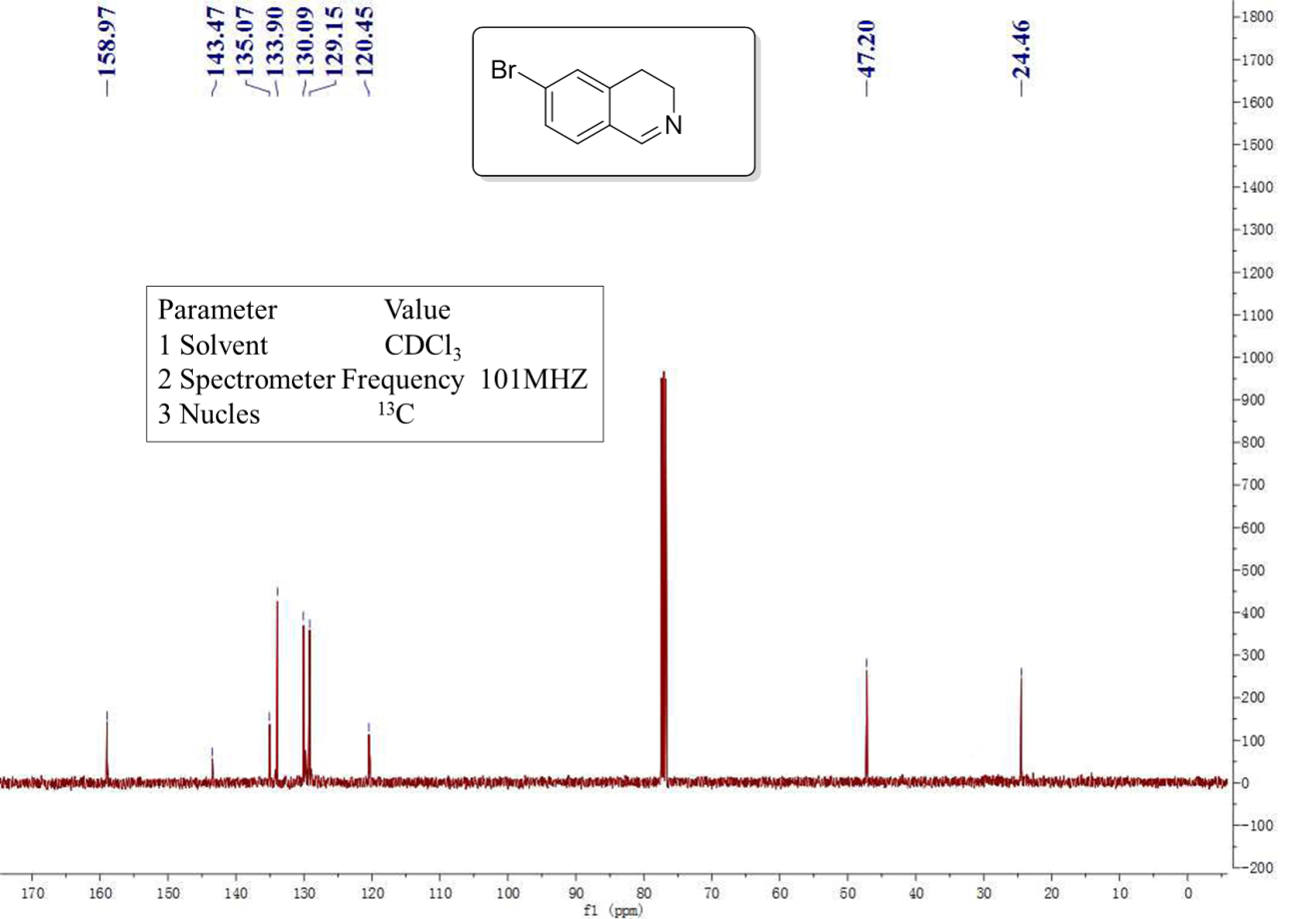


**Supplementary Figure 35 ^13^C NMR spectra of the 6-bromo-3,4-dihydroisoquinoline**


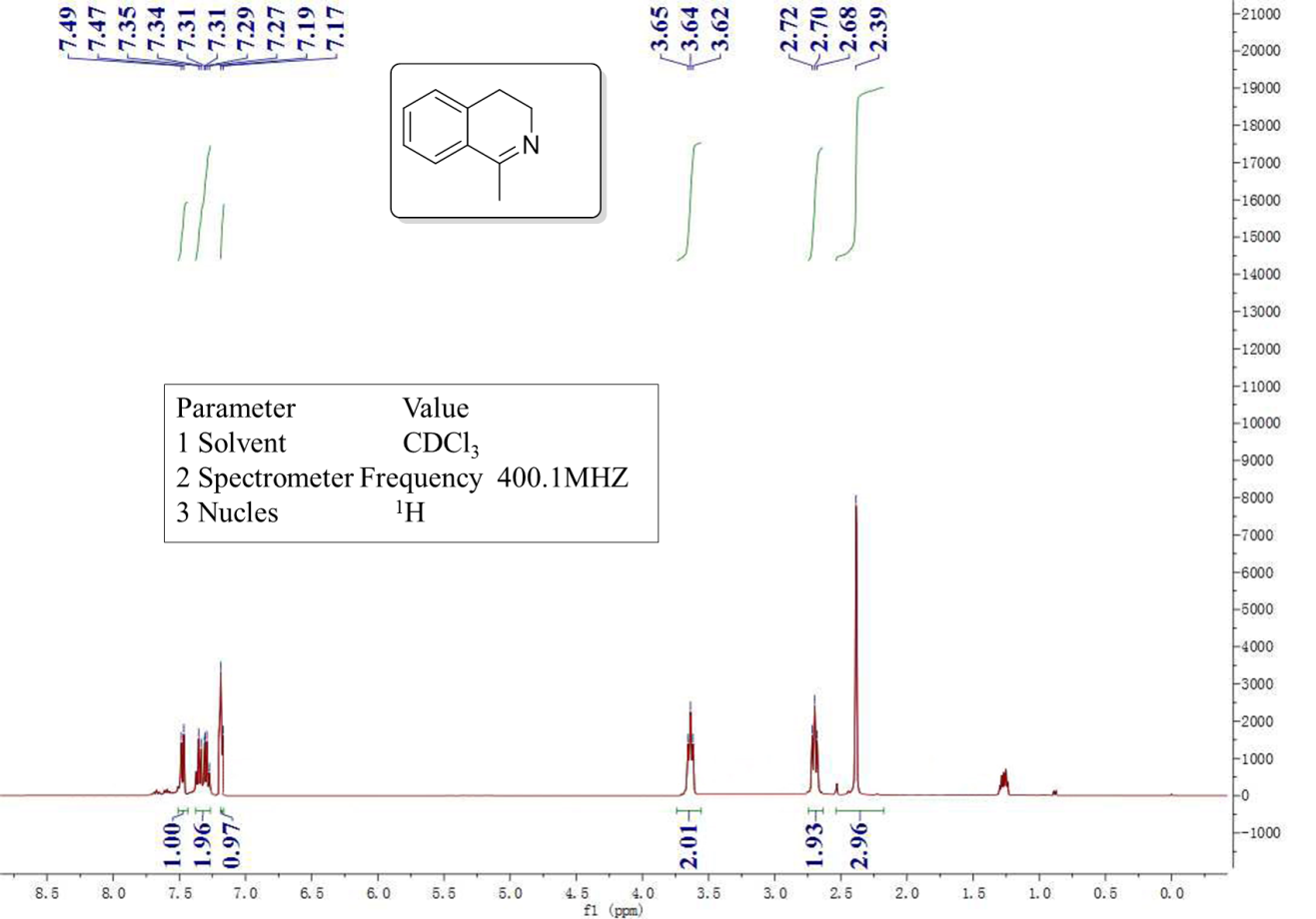


**Supplementary Figure 36 ^1^H NMR spectra of the 1-methyl-3,4-dihydroisoquinoline**


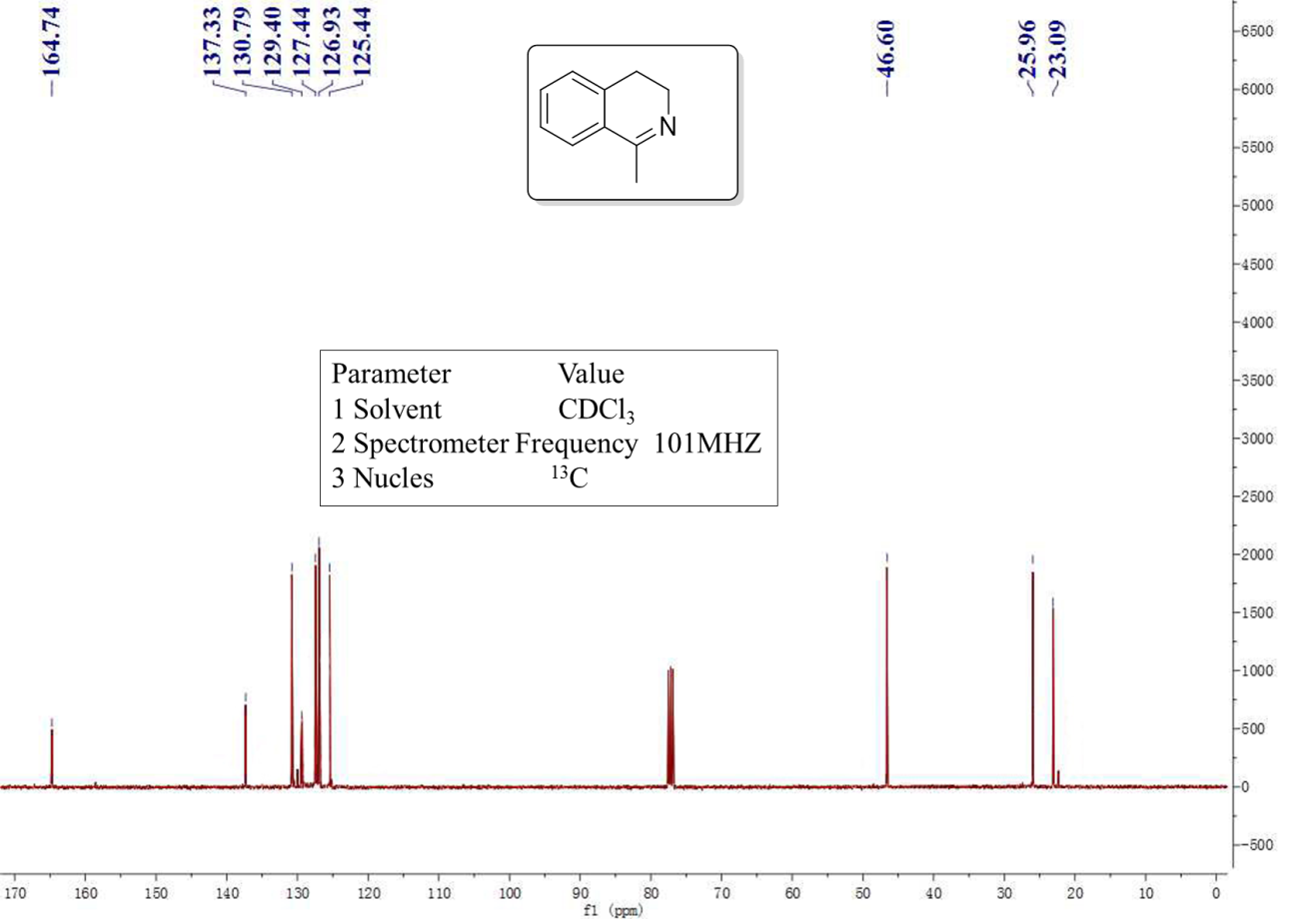


**Supplementary Figure 37 ^13^C NMR spectra of the 1-methyl-3,4-dihydroisoquinoline**


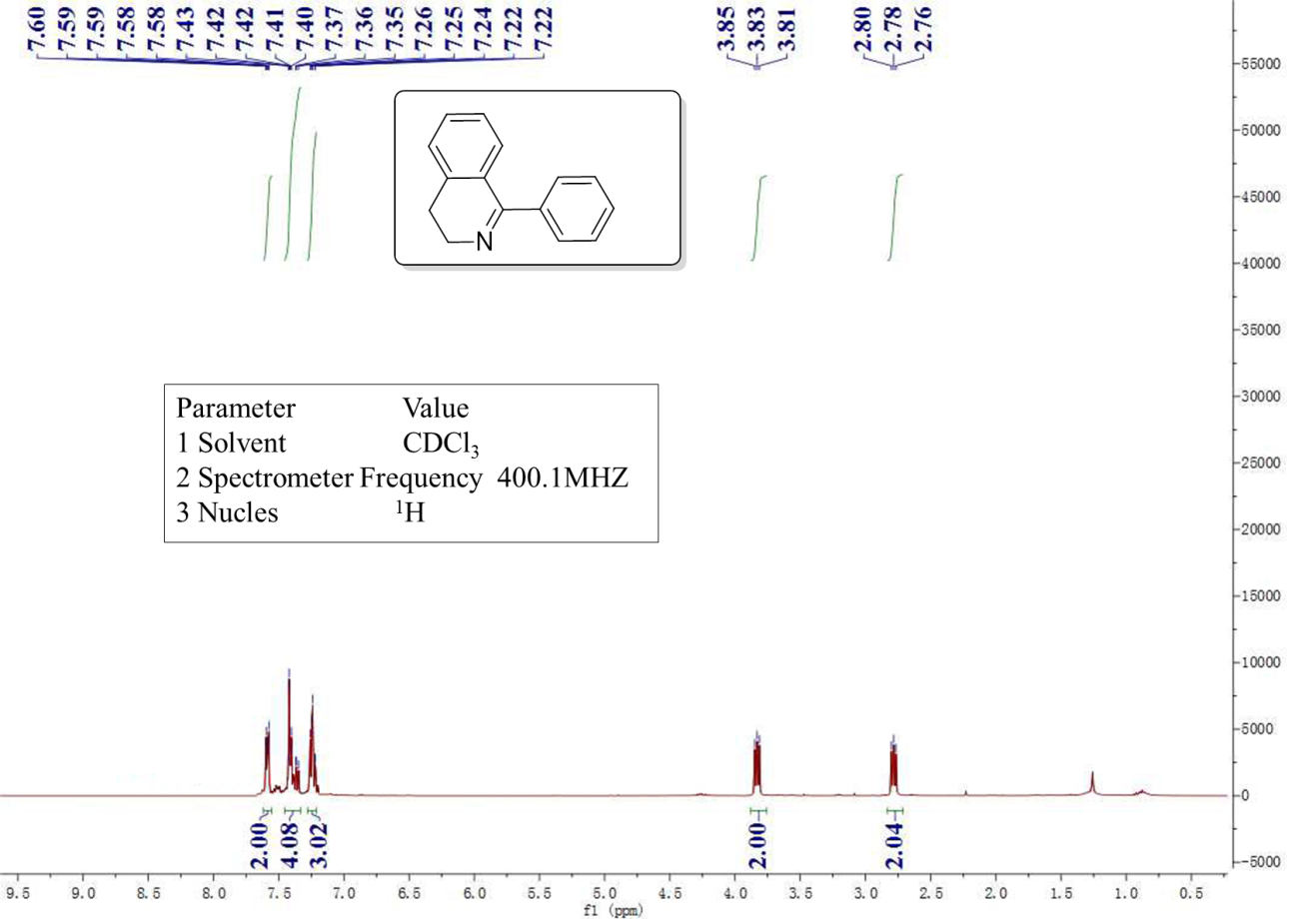


**Supplementary Figure 38 ^1^H NMR spectra of the 1-phenyl-3,4-dihydroisoquinoline**


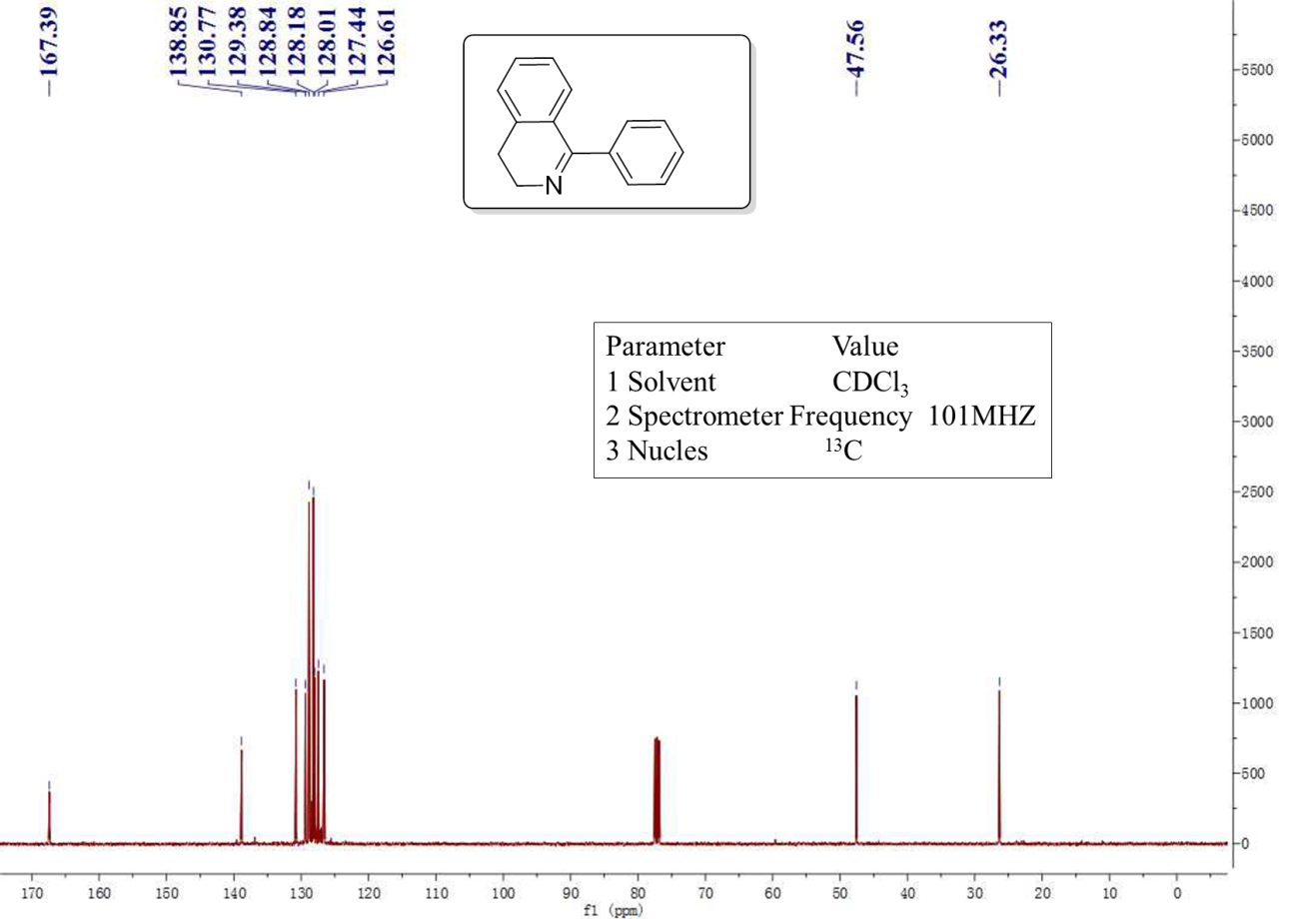


**Supplementary Figure 39 ^13^C NMR spectra of the 1-phenyl-3,4-dihydroisoquinoline**


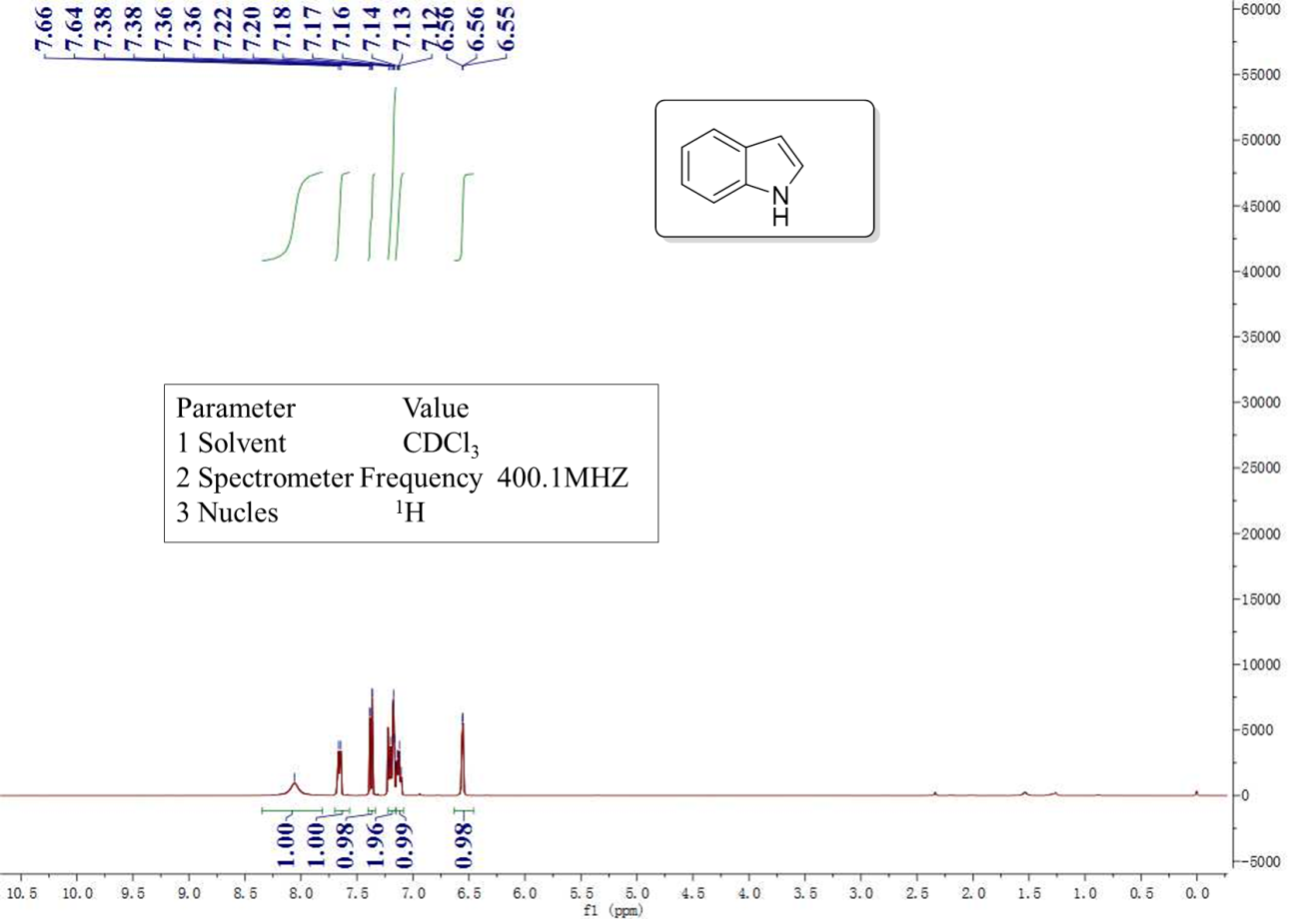


**Supplementary Figure 40 ^1^H NMR spectra of the indole**


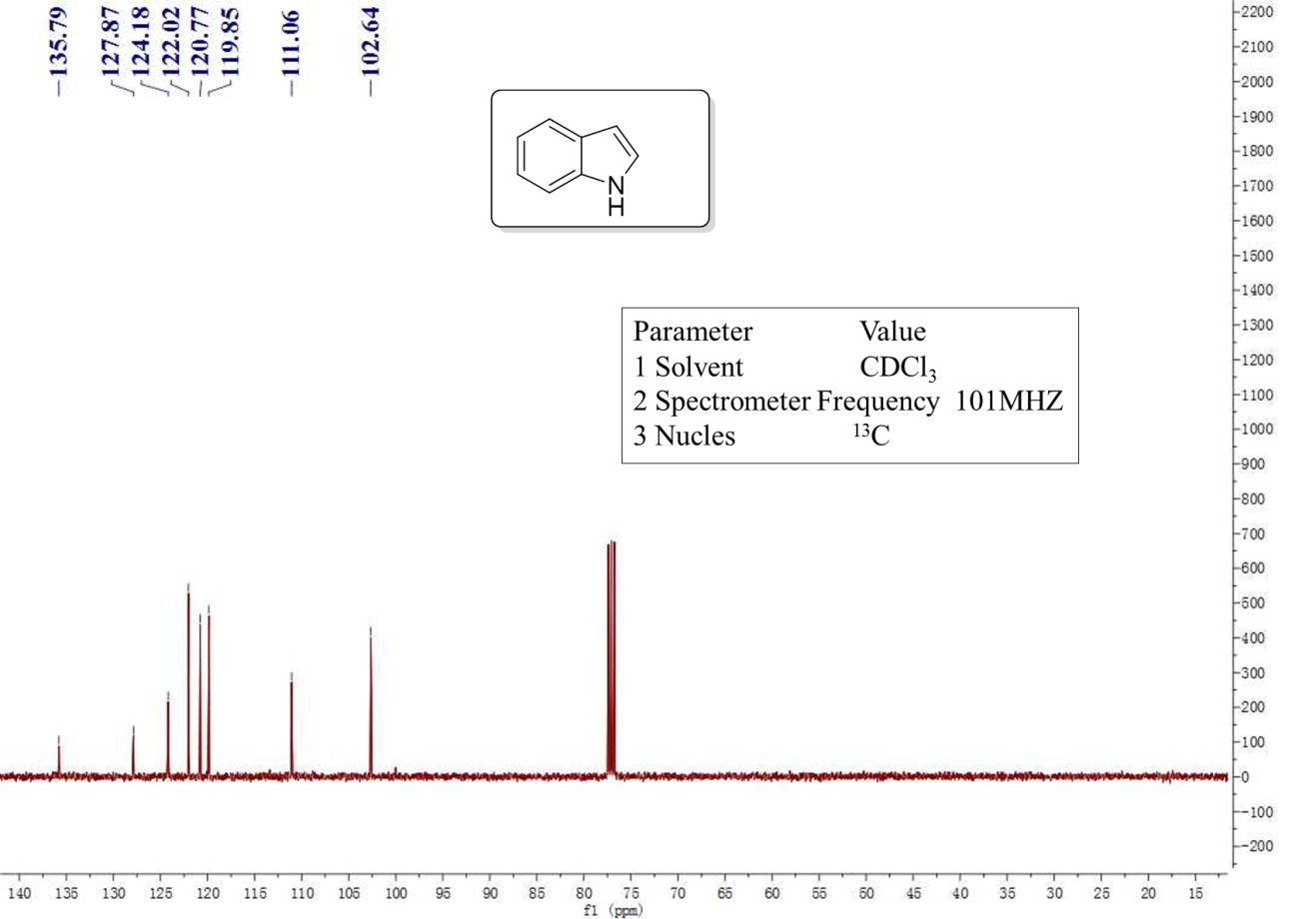


**Supplementary Figure 41 ^13^C NMR spectra of the indole**


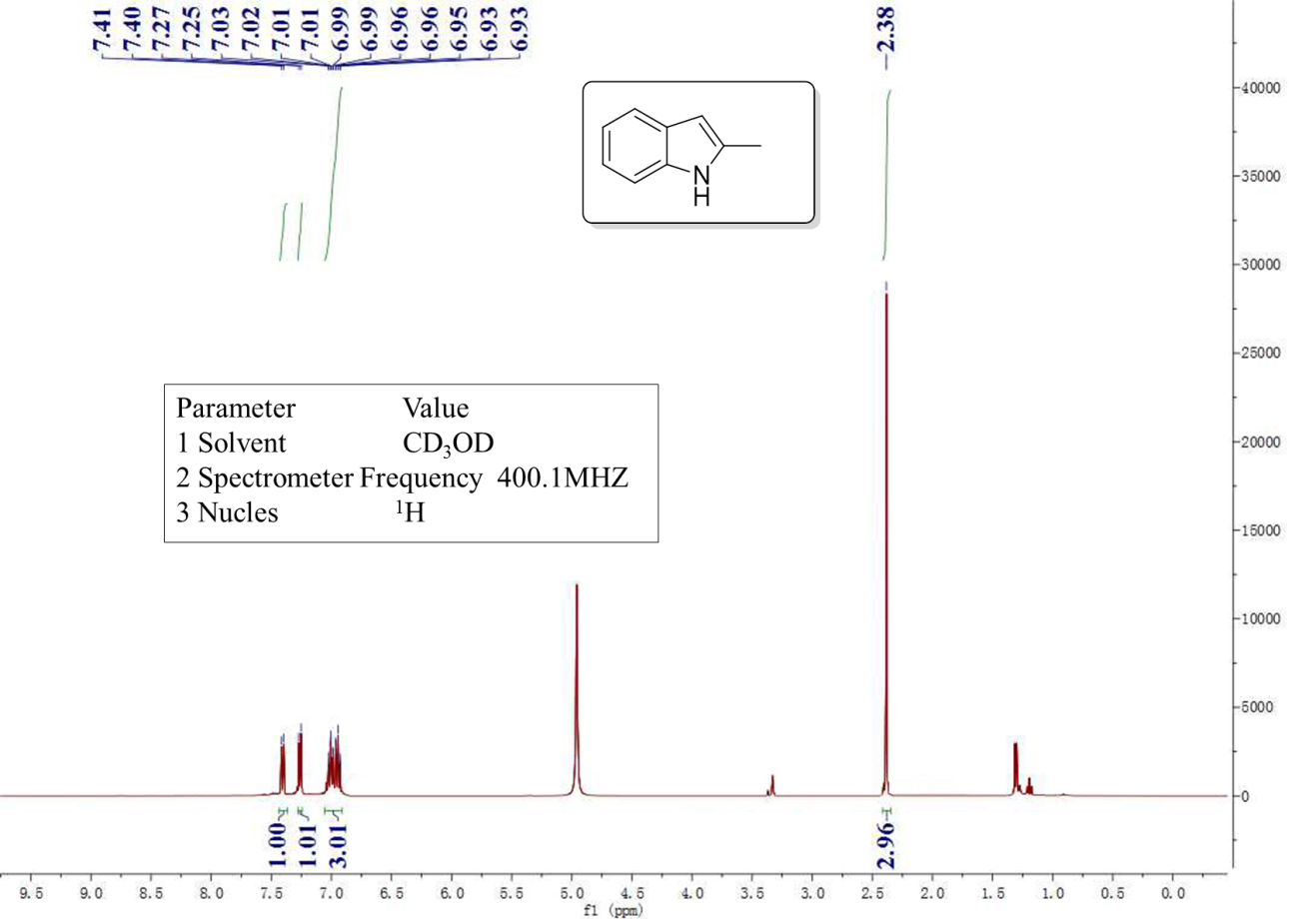


**Supplementary Figure 42 ^1^H NMR spectra of the 2-methyl-1H-indole**


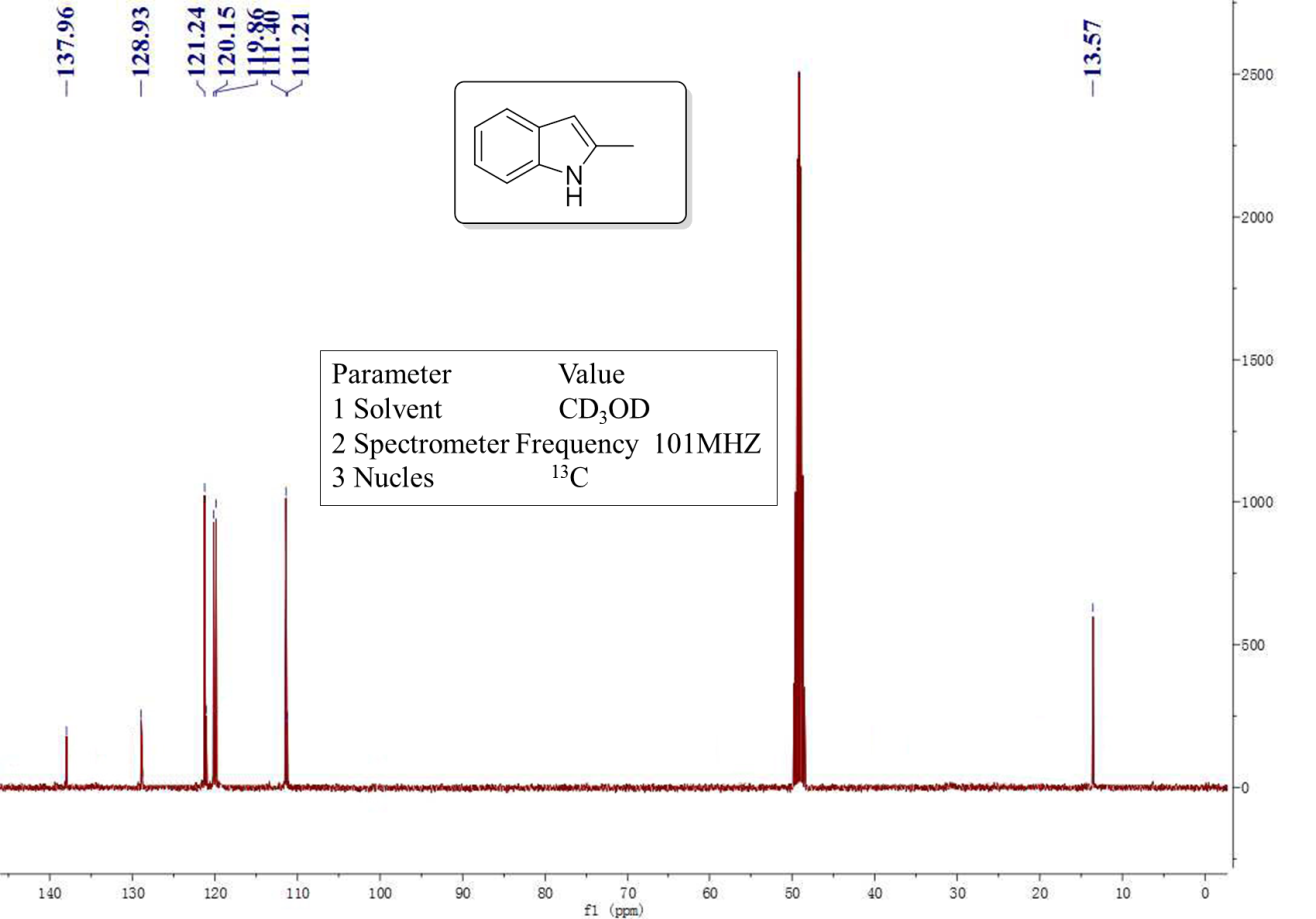


**Supplementary Figure 43 ^13^C NMR spectra of the 2-methyl-1H-indole**


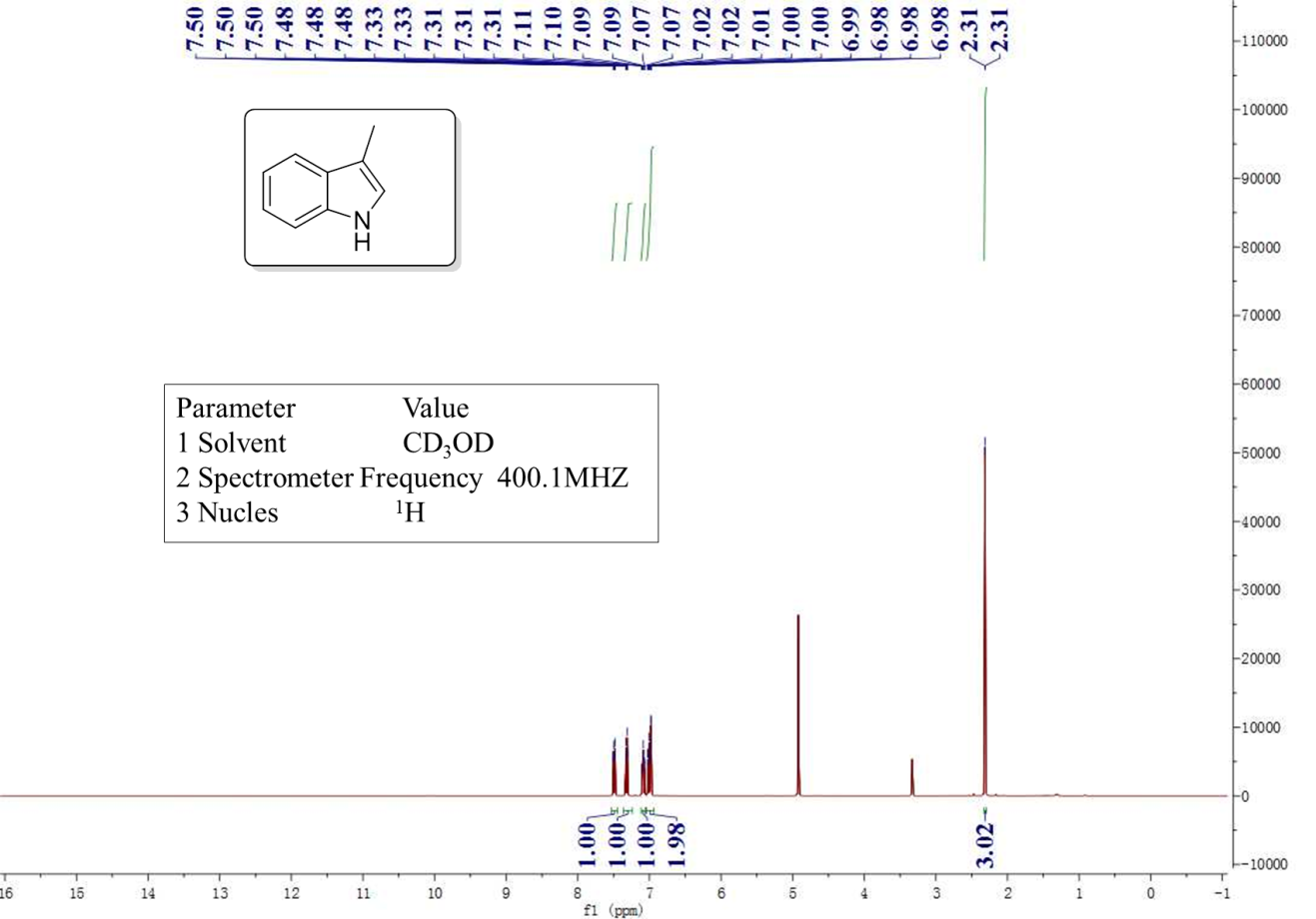


**Supplementary Figure 44 ^1^H NMR spectra of the 3-methyl-1H-indole**


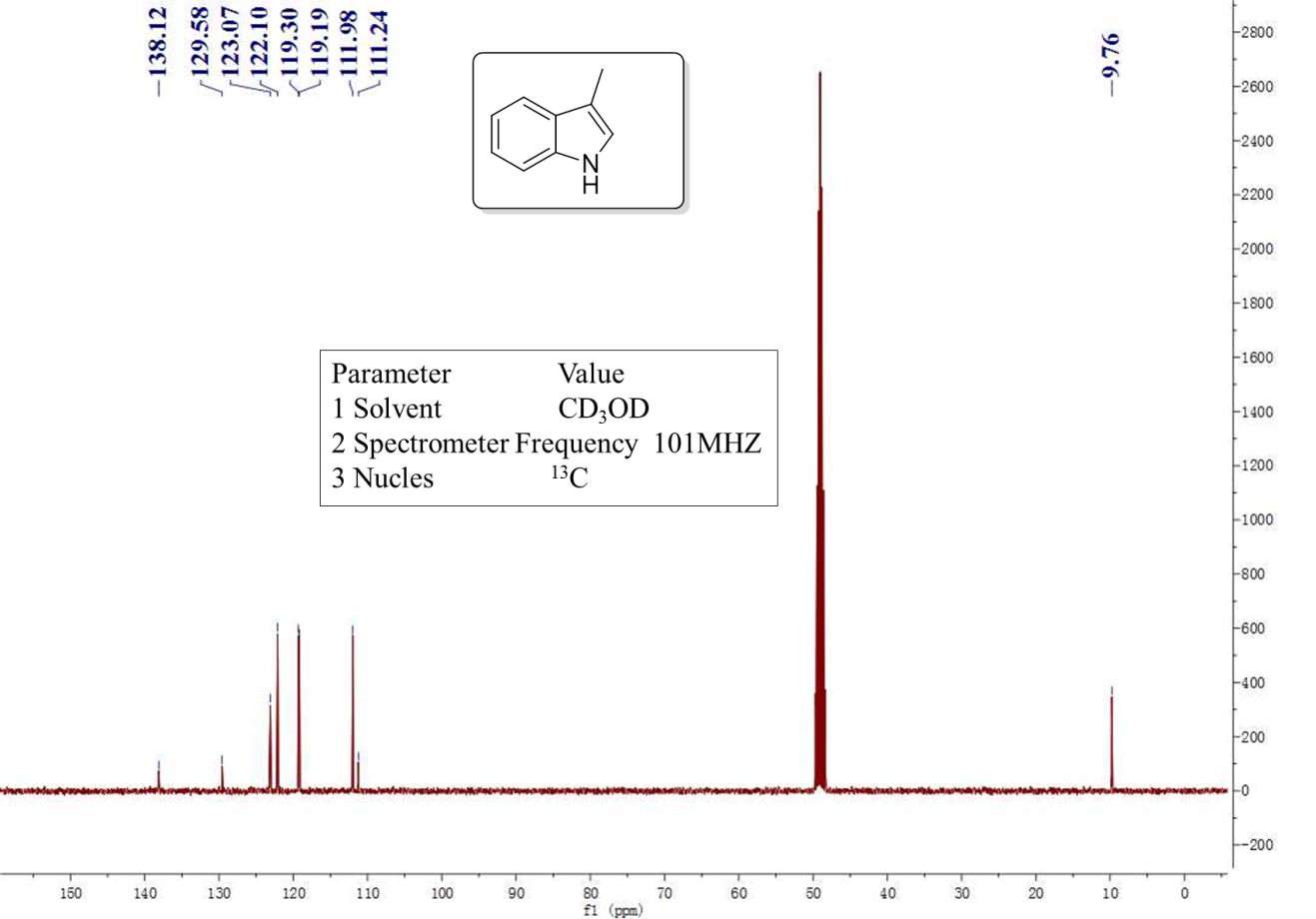


**Supplementary Figure 45 ^13^C NMR spectra of the 3-methyl-1H-indole**


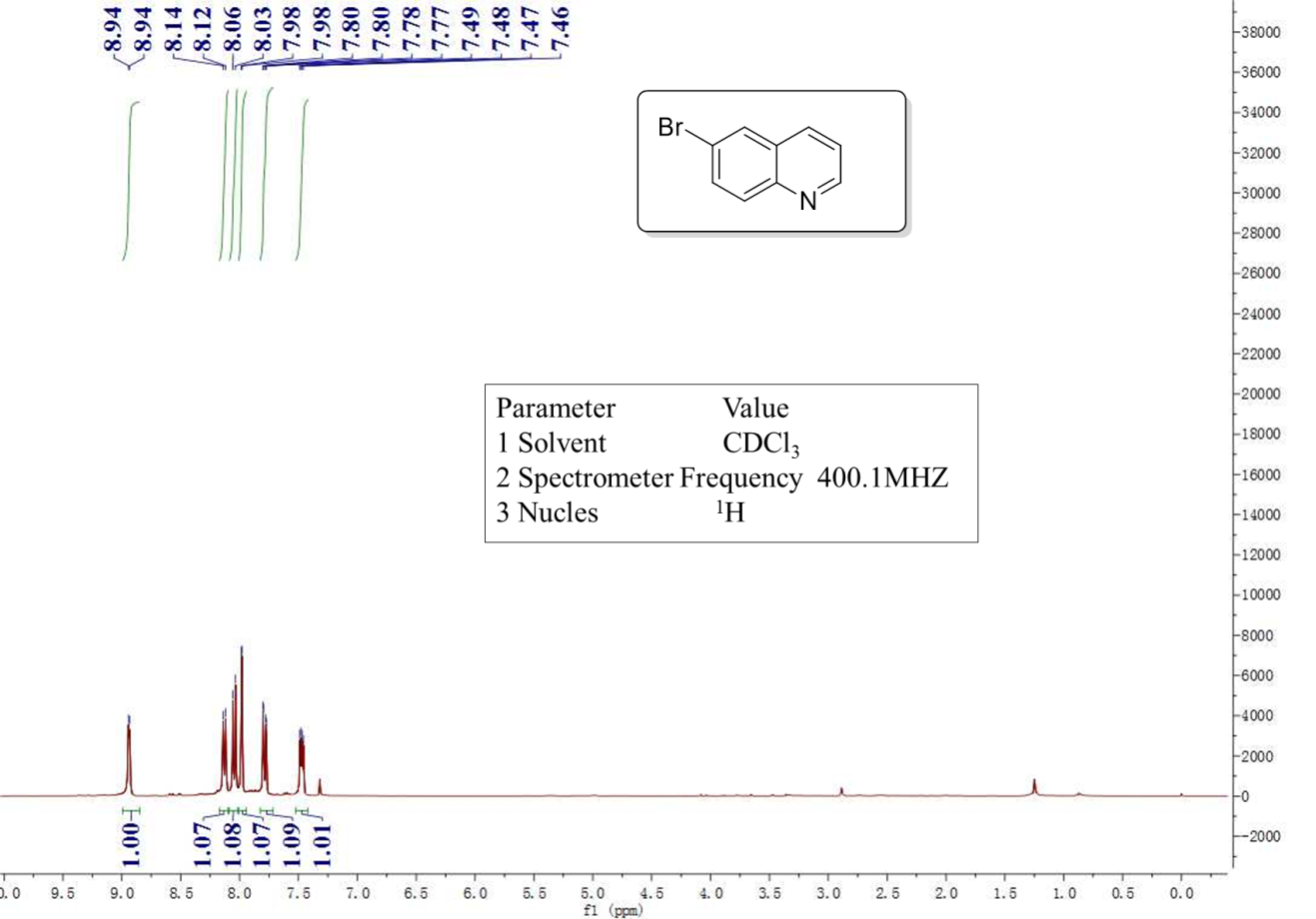


**Supplementary Figure 46 ^1^H NMR spectra of the 5-bromo-1H-indole**


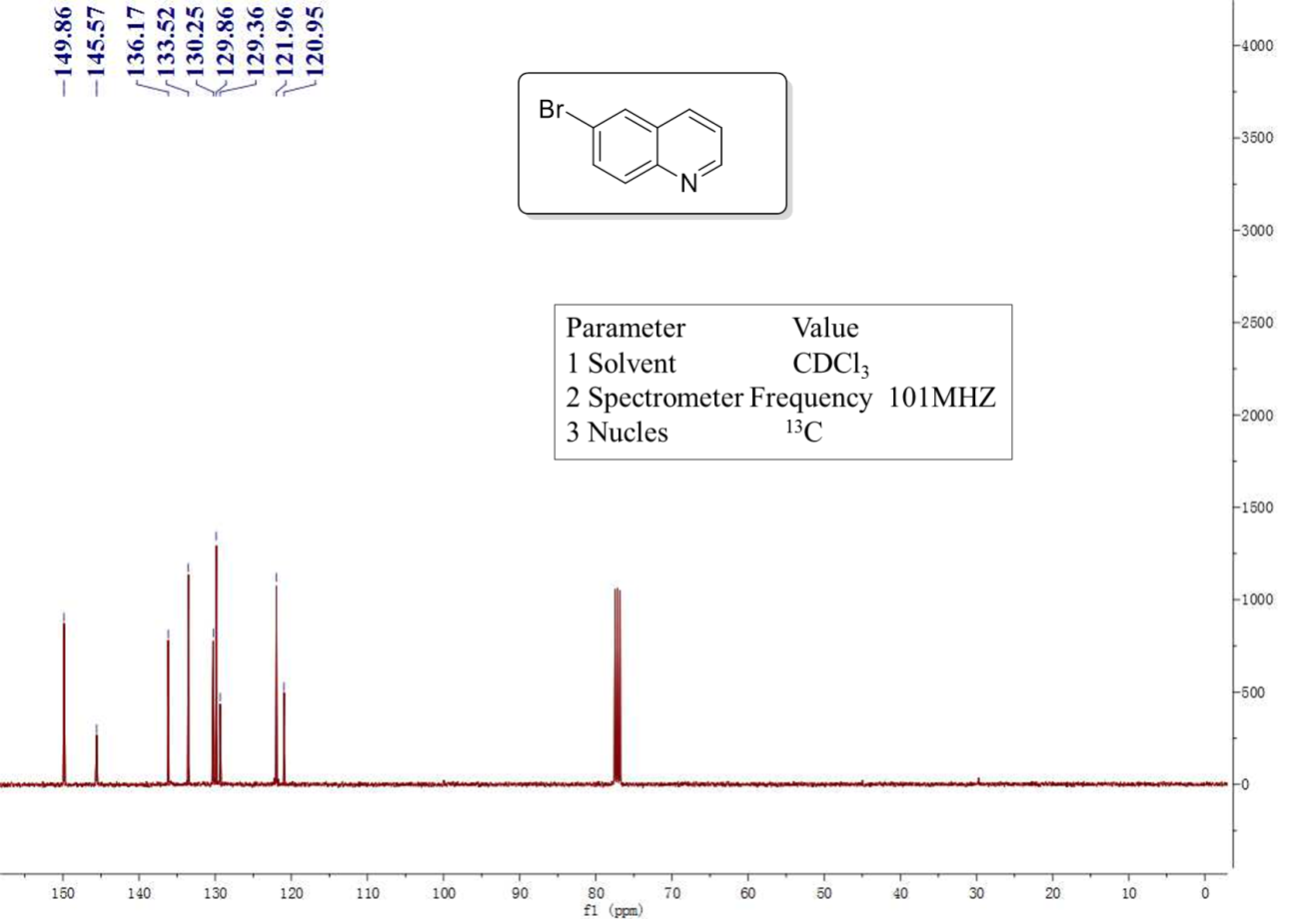


**Supplementary Figure 47 ^13^C NMR spectra of the 5-bromo-1H-indole**


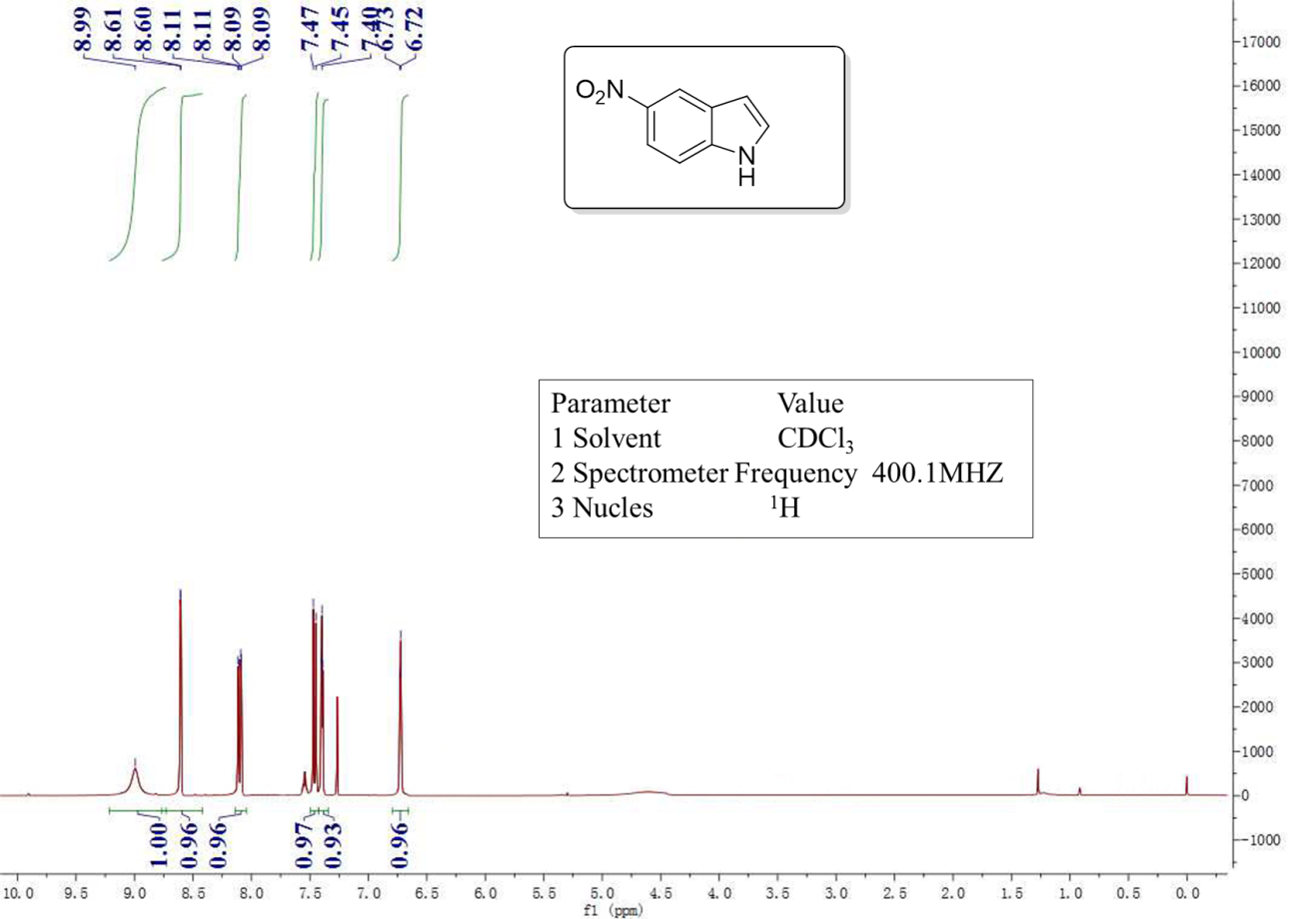


**Supplementary Figure 48 ^1^H NMR spectra of the 5-nitro-1H-indole**


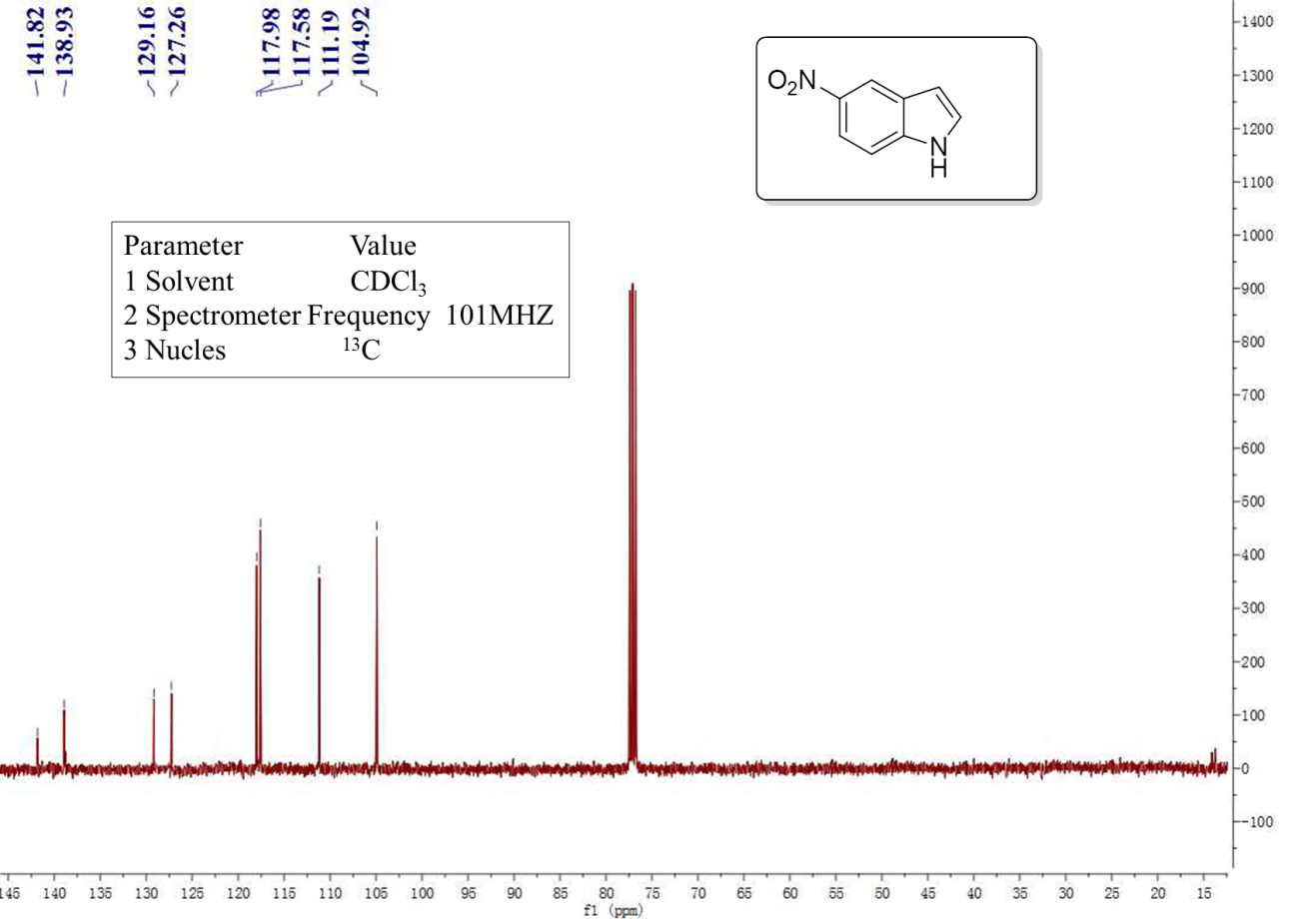


**Supplementary Figure 49 ^13^C NMR spectra of the 5-nitro-1H-indole**


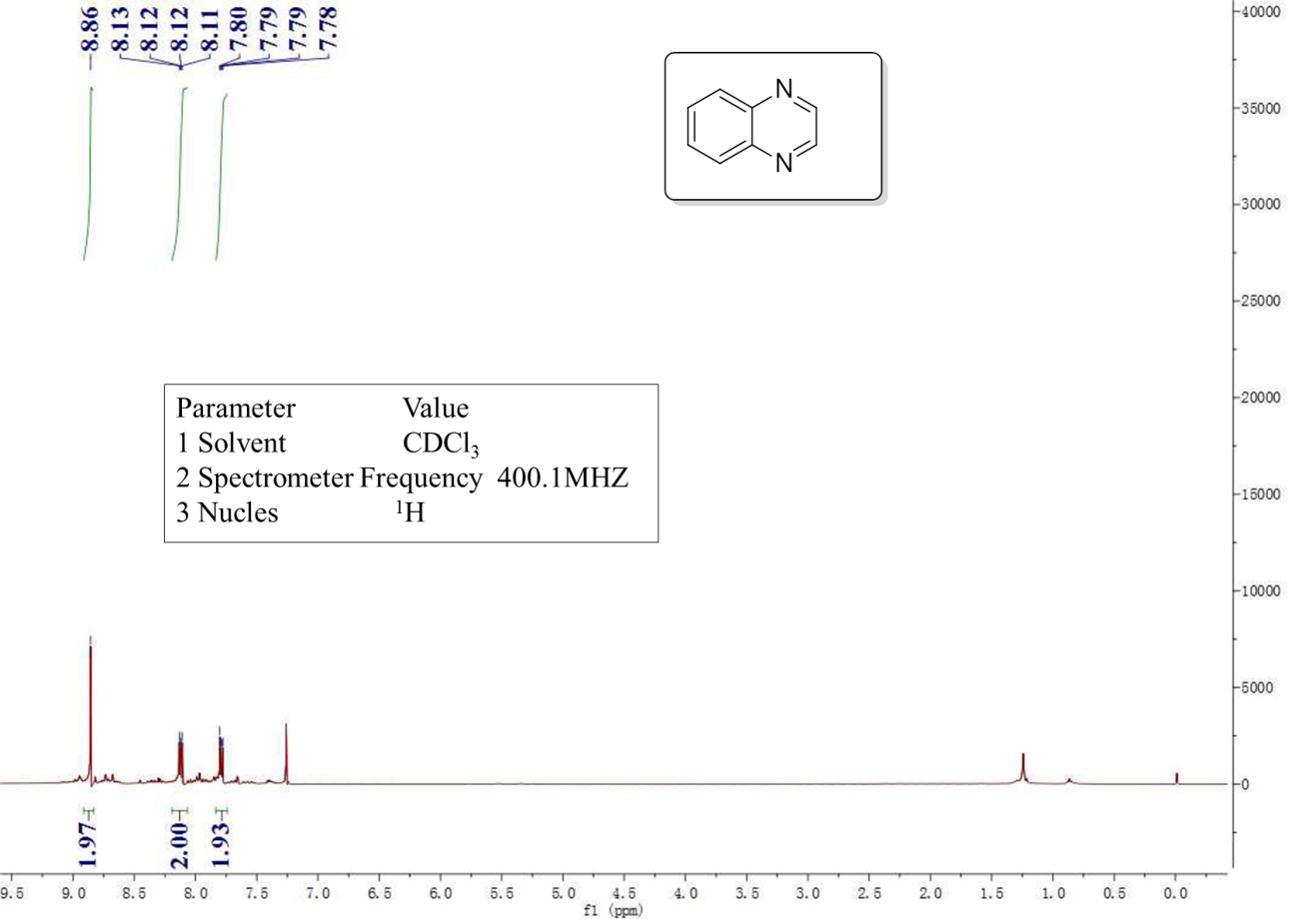


**Supplementary Figure 50 ^1^H NMR spectra of the quinoxaline**


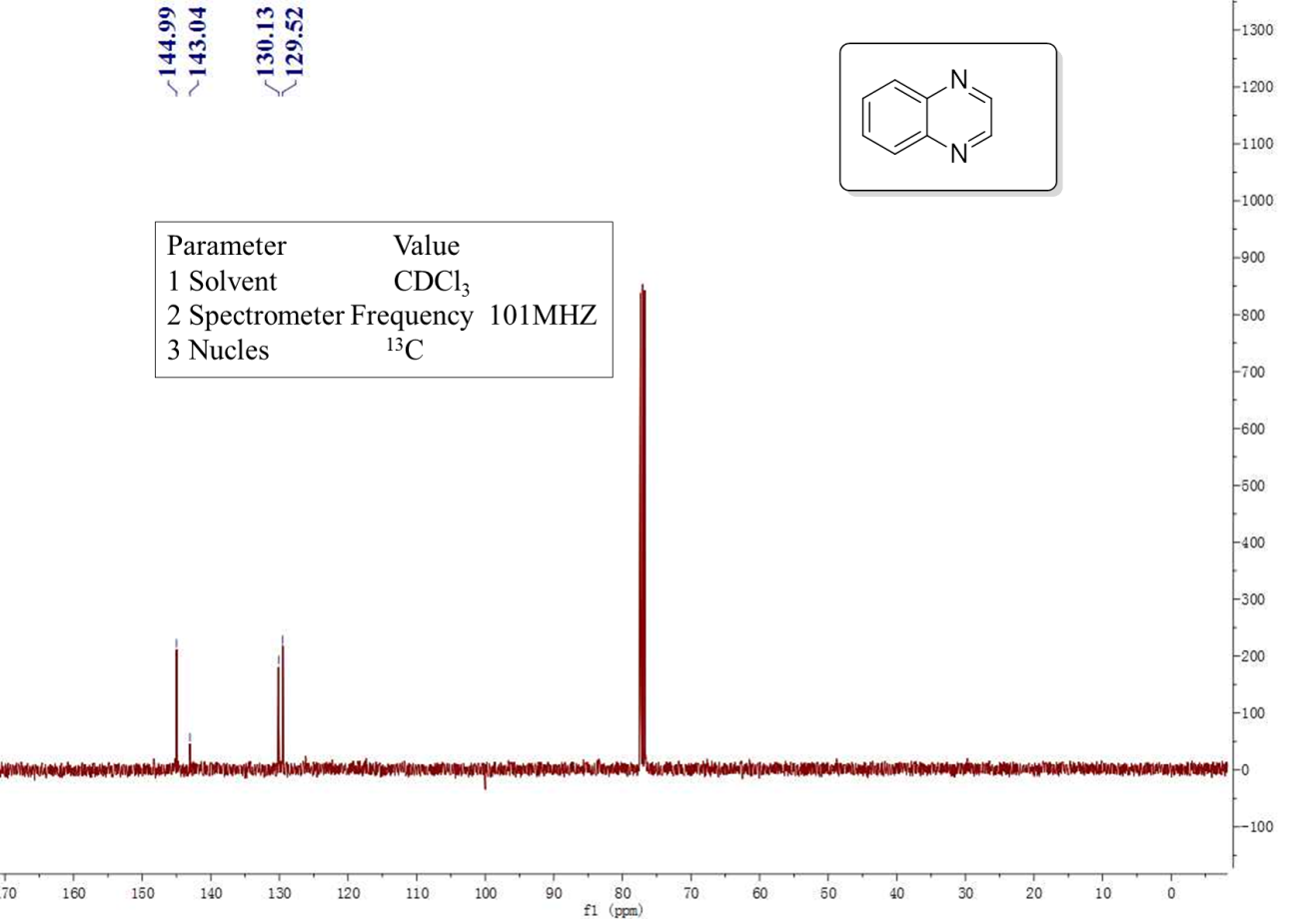


**Supplementary Figure 51 ^13^C NMR spectra of the quinoxaline**

**Supplementary References**

[1] Cui, X. *et al.* *Synthesis and characterization of iron–nitrogen-doped graphene/core–shell catalysts: Efficient oxidative dehydrogenation of N-heterocycles.* *J. Am. Chem. Soc.* **137**, 10652–10658 (2015).

[2] Tanaka, T.; Okunaga, K.-i.; Hayashi, M. *Dehydrogenation of 1,2,3,4-tetrahydroquinoline and its related compounds: comparison of Pd/C–ethylene system and activated carbon–O_2_ system.* *Tetrahedron Lett.* **2010**, **51**, 4633-4635 (2010).

[3] Chakraborty, S.; Brennessel, W. W.; Jones, W. D. *A Molecular iron ctalyst for the acceptorless dehydrogenation and hydrogenation of N-heterocycles.* *J. Am. Chem. Soc.* **136**, 8564-8567 (2014).

[4] Mullick, K.; Biswas, S.; Angeles-Boza, A. M.; Suib, S. L. *Heterogeneous mesoporous manganese oxide catalyst for aerobic and additive-free oxidative aromatization of N-heterocycles.* *Chem. Commun.* **53**, 2256-2259 (2017).

[5] Muthaiah, S.; Hong, S. H. *Acceptorless and base-free dehydrogenation of alcohols and amines using tuthenium-hydride complexes.* *Adv. Synth. Catal.* **354,** 3045–3053 (2012).

[6] Sahoo, M. K.; Jaiswal, G.; Rana, J.; Balaraman, E. *Organo-photoredox catalyzed oxidative dehydrogenation of N-heterocycles.* *Chem. Eur. J.* **23**, 14167–14172 (2017).
